# Supplementary material for: Ubiquitous atmospheric production of organic acids mediated by cloud droplets
Source: Nature. 2021 May 12;593(7858):233–7. doi: 10.1038/s41586-021-03462-x (PMC8116209; doi:10.1038/s41586-021-03462-x)
Supplement: Supplementary file 1 — The Supplementary Information describes the SAPHIR chamber experiments (Sect. 1), the theoretical calculations of the methanediol + OH reaction (Sect. 2), and the EMAC simulations (Sect. 3). Uncertainty sources in the model and methanediol measurements are discussed in Sect. 4 and 5, respectively. The retrievals of formic acid from ground-based FTIR observations and formaldehyde from OMI satellite measurements are presented in Sect. 6 and 7, respectively. Sect. 8 contains supplementary figures. The Supplementary References are listed in Sect. 9. Sect. 10 contains the raw quantum chemical data. [file 41586_2021_3462_MOESM1_ESM.pdf]

nature portfolio

https://doi.org/10.1038/s41586-021-03462-x

Supplementary information

Ubiquitous atmospheric production of organic acids mediated by cloud droplets

In the format provided by the authors and unedited

Ubiquitous atmospheric production of organic acids mediated by warm clouds

By Franco *et al.* (2021)

Supplementary Information

Table of contents

1. Simulation chamber experiments p. 2
2. Theoretical calculations p. 7
3. Model simulations p. 15
4. Methanediol measurements p. 20
5. Remaining sources of uncertainties in model simulations p. 21
6. Model comparison with ground-based FTIR measurements p. 22
7. Model comparison with OMI column measurements p. 24
8. Supplementary figures p. 25
9. References p. 28
10. Raw quantum chemical data p. 34

1. Simulation chamber experiments

The experiments for the detection and chemical transformation of methanediol were performed in the atmosphere simulation chamber SAPHIR (Simulation of Atmospheric PHotochemistry In a large Reaction Chamber; Forschungszentrum Jülich, Germany). The chamber has a volume of 270 m^3 and consists of a double wall Teflon (FEP) foil being highly transparent to solar radiation. A shutter system allows fast switching between illumination and dark conditions. The air provided is mixed from ultra-pure nitrogen and oxygen (Linde, 6.0). A steady flow of air is used to replenish the air consumed by instruments and due to small leakages, while the pressure of the chamber is constantly kept slightly higher than ambient (~30 Pa). The chamber air can be humidified by evaporating ultra-pure water (Milli-Q-Water) into the air stream of the chamber. Fans inside the chamber ensure rapid mixing within 2 minutes. Further details can be found in Ref. [1].

Formaldehyde (HCHO) and methanediol ($HOCH_2OH$) were introduced into the humidified chamber by the injection of formalin solution (>36.5%, Riedel-de Haen) with a microliter syringe. Upon injection, a fine mist of the solution is produced which is evaporated in a heated flow of dry, synthetic air and subsequently flushed into the chamber. Alternatively, an aqueous solution of formaldehyde has been nebulized in an external chamber at ambient temperature and the fine fraction of the formed aerosol has been flushed into the humidified SAPHIR chamber. Both injection methods provided a mix of formaldehyde and methanediol with a maximum of ca. 50% methanediol. As a reference, only formaldehyde was injected into the chamber by pyrolysis of para-formaldehyde (puriss, Riedel-de Haen) from a closed vessel flushed by a permanent nitrogen flow (SI Tab. 1).

Experimental procedures were similar for all experiments. After humidification the flushed chamber was illuminated for approximately 2 hours to determine chamber sources of formaldehyde, formic acid (HCOOH) and other volatile organic compounds (VOCs). The chamber roof was then closed for injection of formaldehyde and methanediol followed by a dark phase for the determination of possible wall loss or conversion processes. Subsequently, a second illumination phase of several hours duration allowed the study of photochemical processes in addition to the previously determined chamber sinks and sources. Complementary, 250 ppm carbon monoxide (CO) was used as an OH scavenger during one experiment to identify photolytical loss processes.

a. Instrumental

The sum of formaldehyde and methanediol was detected by means of a Hantzsch-Monitor (AL4001, AeroLaser, Germany). The air sample is pumped through a temperature controlled stripping coil where formaldehyde and methanediol are dissolved in 0.05 mol/L sulfuric acid solution. Within the aqueous solution formaldehyde is hydrated and instates an equilibrium with methanediol. The aqueous solution is then mixed with the Hantzsch reagent to form a dye from both compounds which is detected fluorimetrically. The instrument was calibrated weekly from aqueous formaldehyde standards providing an accuracy of 6% and a limit of detection (1σ) of 25 ppt. Details of the instrument setup can be found in Ref. [2].

Additionally formaldehyde and OH radicals were measured in-situ by differential optical absorption spectroscopy (DOAS) at 308 nm (see Ref. [3]). The limit of detection was 0.23 ppb for formaldehyde and 8×10^5 molecules cm^{-3} for OH.

The methanediol concentration was derived from the difference of the Hantzsch and DOAS measurements. Methanediol was only detected during injections from aqueous formaldehyde solutions whereas DOAS and Hantzsch agree within their errors during the reference experiment (SI Tab. 1), when dry gaseous formaldehyde has been injected to humidified SAPHIR.

VOCs were measured by a proton transfer reaction time-of-flight mass spectrometer (PTR-TOF-MS-8000, Ionicon, Austria)[4] operated at a drift tube voltage of 590 V and a constant pressure of 2.2 mbar. In particular, formic acid was detected at its protonated molecular mass (m/z = 47.01276). Calibrations were performed using a Liquid Calibration Unit (LCUa, Ionicon, Austria) with aqueous formic acid solutions of known concentration. The accuracy, i.e. the 1σ-error of the sensitivity, is 3%. Other VOCs were calibrated using a self-made gas standard containing among others methanol (CH_3OH) and acetaldehyde (CH_3CHO) at mixing ratios of ~600 ppb. The gas standard was further diluted by factors of 1/500 – 1/50 by the LCUa before being measured by PTR-TOF-MS.

The OH reactivity (the inverse of the OH radical lifetime) was measured with a laser photolysis-laser induced fluorescence (LP-LIF) instrument[5, 6].

Supplementary Information Table 1 | SAPHIR experiment conditions.

Date	Injection	$HOCH_2OH$ (max.)	$HOCH_2OH + HCHO$ (max.)	Oxidation	Purpose
2018-04-07	Spraying $HCHO_{(aq)}$ by aerosol generator	8 ppb	39 ppb	4×10^6 cm^{-3} OH	HCOOH formation
2018-04-09	Pyrolysis of para-HCHO	0 ppb	28 ppb	2×10^6 cm^{-3} OH	Reference
2018-04-10	Spraying $HCHO_{(aq)}$ by aerosol generator	10.5 ppb	42 ppb	UV-Lights<br>7×10^6 cm^{-3} OH	HCOOH formation
2018-06-28	Syringe injection of Formalin-Solution (37% $HCHO_{(aq)}$)	9 ppb	20 ppb		HCOOH formation
2018-07-13	Syringe injection of Formalin-Solution (37% $HCHO_{(aq)}$)	18 ppb	33 ppb	7×10^6 cm^{-3} OH<br>250 ppm CO, no OH (2nd phase)	HCOOH formation w/wo CO

b. Model calculations

Model calculations were performed using the Master Chemical Mechanism (MCM) in its latest version 3.3.1 (http://mcm.leeds.ac.uk/MCM/). The model was constrained to measured values of temperature, pressure and OH concentration. Photolysis frequencies were calculated from the measured actinic flux. Injections of formaldehyde, methanediol and CO were modelled as sources being present during the times of injection.

The chamber source of formic acid was modelled as a continuous source during times of the experiment, when the chamber air was exposed to sunlight. The source strength was adjusted to match the observed increase of formic acid during times when OH was not present, i.e. when formic acid was not chemically produced from the reaction of methanediol with OH. This was the case, when the chamber roof was closed before the injection of formaldehyde and methanediol, and after the injection of excess CO as OH scavenger. Both periods give consistent source strengths. The uncertainty of the source strength was determined from sensitivity model runs, for which the value was varied:

source → HCOOH (k = (4.2 ± 1) s^{-1})

This gives an increase of formic acid of approximately 0.6 ppbv $hour^{-1}$.

After the injection of formaldehyde and methanediol, methanediol concentrations immediately decrease also under dark conditions, when no chemical reaction of methanediol is expected. A decrease in the concentration is also observed after CO has been injected as OH scavenger. At the same time formaldehyde concentrations increase. To account for this, a conversion reaction of methanediol that produces formaldehyde is implemented. The conversion rate constant is adjusted such that to match the decrease of methanediol in the absence of OH. Sensitivity model runs were used to determine the uncertainty of this approach:

$HOCH_2OH$ → HCHO + H_2O (k = (8.5 ± 1) × 10^{-5} s^{-1})

In addition, the reaction of methanediol and OH was added to the MCM. The reaction rate constant was adjusted to match the faster decrease of methanediol in the presence of OH after the chamber was opened and before CO was injected (see SI Sect. 1.c). The best match is achieved using a reaction rate constant of $k = 7.5 \times 10^{-12}$ cm^3 s^{-1} (SI Fig. 1):

$HOCH_2OH$ + OH → HO_2 + HCOOH ($k = 7.5 \times 10^{-12}$ cm^3 s^{-1})

The uncertainty of the reaction rate constant was determined by varying its value in sensitivity model and adjusting the chamber source strength for formic acid and the conversion reaction rate constant of methanediol, such that measured concentrations of methanediol, formaldehyde and formic acid are matched. This results in a range for its value between 1 and 10 × 10^{-12} cm^3 s^{-1}, for which all observations are described by the model within 5%.

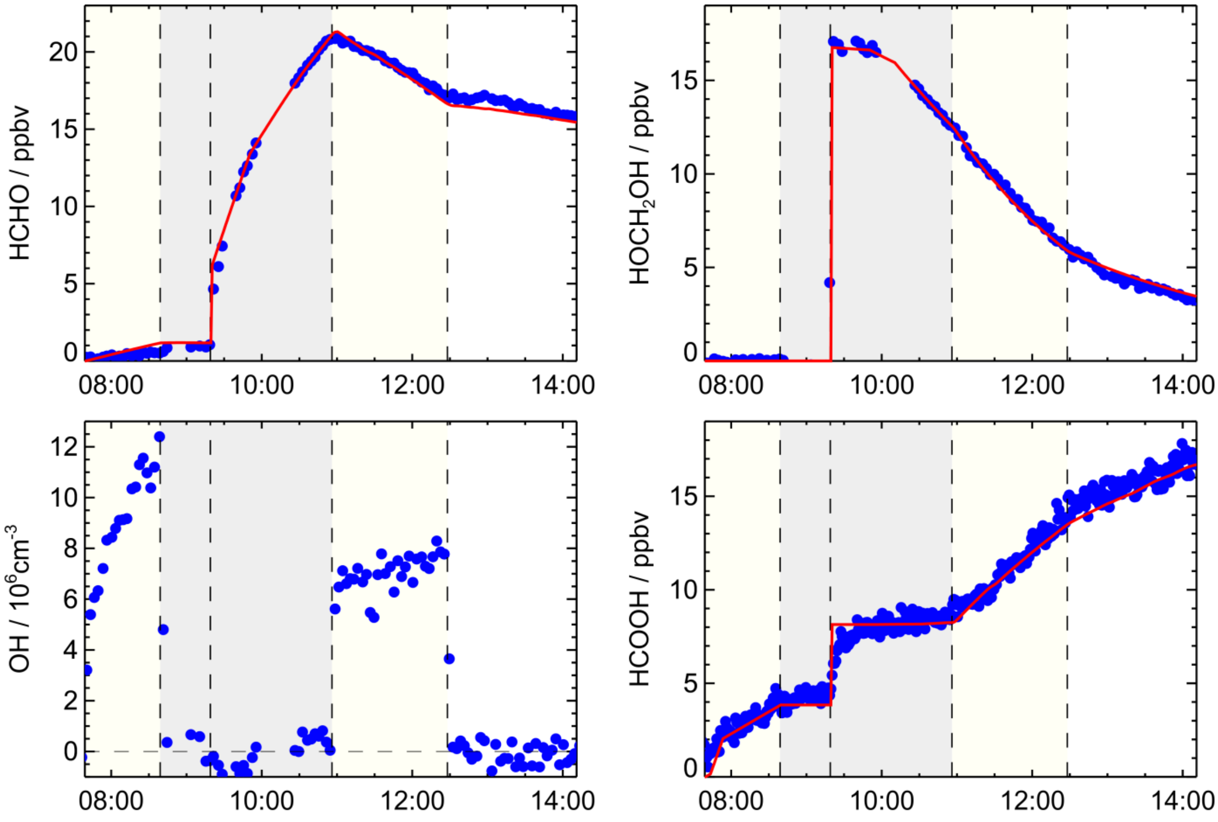

Supplementary Information Figure 1 | Experiment in the chamber SAPHIR. Time series of measured HCHO, $HOCH_2OH$, HCOOH and OH (blue dots) together with results from chemical box modelling (red lines) during an experiment in the simulation outdoor chamber SAPHIR. Grey shaded areas indicate conditions without sunlight and white areas indicate times when excess CO was present as OH scavenger.

c. Experimental determination of the rate coefficient between methanediol and OH radicals

The rate coefficient for the reaction of methanediol with OH radicals was determined experimentally for two different experiments in the SAPHIR chamber. In the initial phase of the first experiment (SI Fig. 2) before the injection of formalin, there is a small OH reactivity (up to 2 s^{-1}) which is due to the trace gases generated from the wall. After the contribution of the measured trace gases is removed from the OH reactivity, an average 0.5 s^{-1} missing reactivity remained. After the injection of formalin, the largest fraction of OH reactivity is due to formaldehyde, methanol and acetaldehyde. After removing the missing reactivity observed before injection of formalin, the remainder reactivity observed, on average 1.6 ± 0.25 s^{-1}, was assigned to the reaction between methanediol and OH radicals. The concentration of methanediol was obtained from the difference between the Hantzsch and the DOAS measurements and it was, on average, 7.3(±3.2) × 10^{10} cm^{-3}. The rate coefficient for the reaction between methanediol and OH radicals (2.2(±1.4) × 10^{-11} cm^3 s^{-1}) was then obtained from the ratio between the measured OH reactivity and the concentration of methanediol.

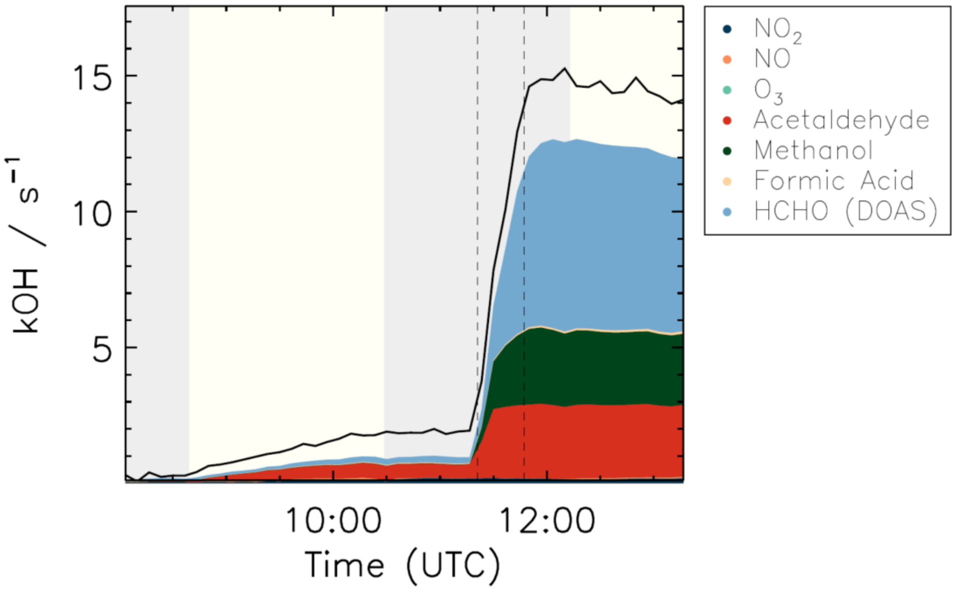

Supplementary Information Figure 2 | Experiment with sunlight. The yellow areas denote the time when the chamber roof was open. The vertical dashed lines indicate the injection of the formalin solution in the chamber.

The second experiment analysed (SI Fig. 3) was characterized by the use of UV-lamps to produce the OH radicals in the chamber instead of sunlight. This resulted in a negligible formation of trace gases from the chamber wall. The difference between the measured and the calculated OH reactivity after the injection of formalin (on average 0.5 ± 0.3 s^{-1}) was used to determine the rate coefficient for the reaction between methanediol and OH radical, which is 1.8(±1.2) × 10^{-11} cm^3 s^{-1}. Although the determined rate coefficients carry a large uncertainty due to the small residual OH reactivity used for the calculation (still larger than the limit of detection of the instrument[1], 0.2 s^{-1}), they agree with each other and they are consistent with the value determined with the model calculation.

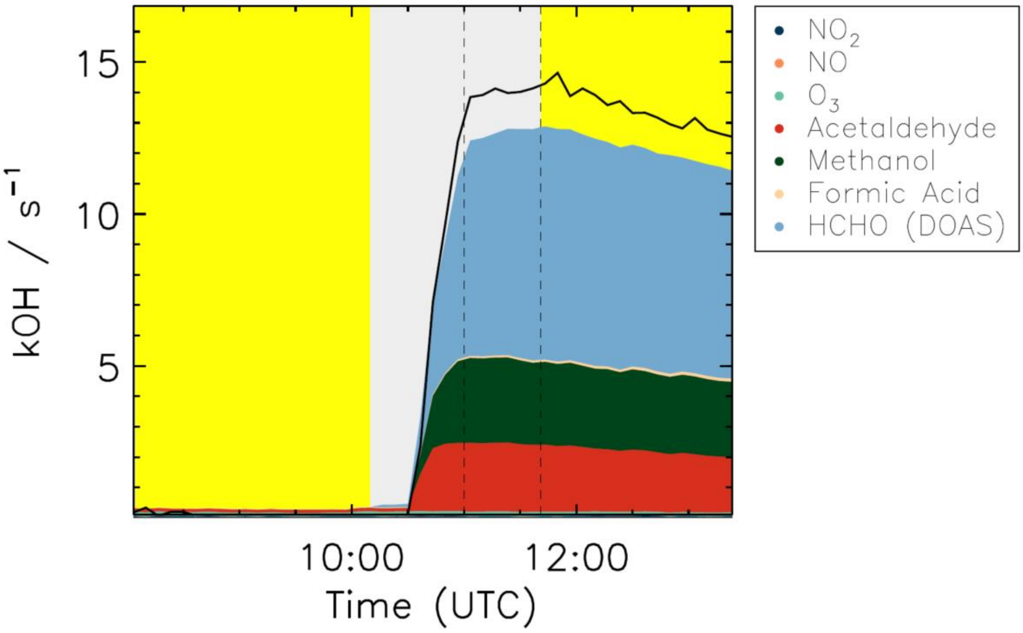

Supplementary Information Figure 3 | Experiment with UV-lamps. The yellow areas denote the time when the UV-lamps were on. The vertical dashed lines indicate the injection of the formalin solution in the chamber.

2. Theoretical calculations

a. Methodology

i. Quantum chemical calculations

The geometries of all reactants, products, intermediates, complexes and transition states are first optimized at the M06-2X/aug-cc-pVDZ and M06-2X/aug-cc-pVTZ levels of theory[7-10], using an integration grid of 99 radical shells and 590 angular points ('ultrafine'). For those structures that could have multiple conformers, an exhaustive search was performed, sampling the conformer space by starting optimizations at a large number of initial geometries, as appropriate for the degrees of freedom allowing for distinct conformers. All compounds and their distinct conformers thus found were re-optimized at the M06-2X/aug-cc-pVQZ level of theory, with a vibrational analysis at the same level of theory, in all cases using an integration grid of 175 radical shells and 974 angular points ('superfine'). The relative energy was further improved by a set of CCSD(T)/aug-cc-pVxZ single point energy calculations (x = D, T, Q)[11], and the obtained energies extrapolated to the complete basis set limit using the aug-Schwartz6 scheme introduced by Ref. [12]; we refer to this methodology as CCSD(T)/CBS(DTQ).

For the transition states of the methanediol (denoted by $CH_2(OH)_2$ in this section) + OH and $C^{\bullet}H(OH)_2$ + O_2 reactions, IRC calculations were additionally performed at the M06-2X/aug-cc-pVQZ level of theory using small steps of 0.5 Bohr, to ascertain the reaction path. These IRC pathways were then used to perform IRCMax calculations[13], where the optimal geometry was located using CCSD(T)/aug-cc-pVTZ single point calculations on geometries along the M06-2X IRC path. On the selected geometry, we then performed CCSD(T)/CBS(DTQ) energy calculations as described above; these are considered to be the best available activation barriers for the $CH_2(OH)_2$ + OH reaction. The IRC energy profile was also improved by an extensive set of single point CCSD(T)/aug-cc-pVTZ calculation along the IRC path; the resulting energy profile is used in tunneling calculations (see below). The barrierless complexation reaction was characterized by a set of constrained geometry optimizations with frequency analysis at the M06-2X/aug-cc-pVQZ level of theory, for distances between the $CH_2(OH)_2$ and OH reactions spanning 3.5 to 8.5 Å.

All calculations were performed using the Gaussian-09 and Gaussian-16 quantum chemistry software suite[14, 15].

ii. Theoretical kinetic calculations

As for methanol (CH_3OH) + OH, the rate coefficient of the $CH_2(OH)_2$ + OH reaction shows a very complex temperature dependence, owing to the presence of the pre-reactive complex, the competition between redissociation of the complex versus H-abstraction, the energy-specific impact of tunneling, and the lack of a clear saddle point in the barrierless complexation of the reactants; we refer to the theoretical section in Ref. [16] for a more in-depth discussion of these aspects.

In this work, we employ a similar methodology as in Ref. [16] for CH_3OH + OH, following the same reaction scheme and naming:

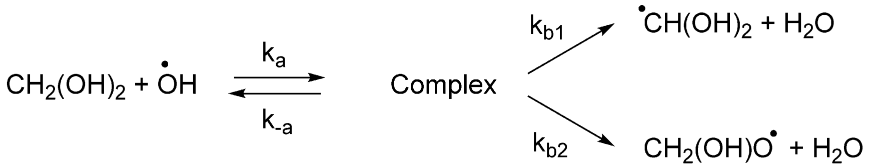

The total rate coefficient is calculated from capture rate coefficient for complexation, k_a, followed by either redissociation to the reactants, k_{-a}, or by H-abstraction from the carbon atom, k_{b1}, or oxygen atom, k_{b2} :

$$k_{HPL}(T) = k_a(T)\frac{k_{b1}(T) + k_{b2}(T)}{k_{b1}(T) + k_{b2}(T) + k_{-a}(T)}$$

The product yield $Y_j(T)$ for product *j* (*j*=1 for $C^{\bullet}H(OH)_2$ and 2 for $CH_2(OH)O^{\bullet}$) is then given by the ratio $Y_j(T) = k_{bj}(T)/\{k_{b1}(T)+k_{b2}(T)\}$. The rate coefficient $k_a(T)$ of the $CH_2(OH)_2$--OH complex formation is based on E,J-resolved micro-variational transition state theory calculations (E,J-μVTST) in an RRHO approximation. The M06-2X/aug-cc-pVQZ data used in this analysis is expected to yield reasonable rate coefficients at the temperatures of interest in the atmosphere. At lower temperatures (<150 K), it is however expected that the current data set is unable to correctly predict the capture rate coefficient, and we refrain from making predictions in this low-temperature regime. The high-pressure rate coefficients $k_{b1}(T)$ and $k_{b2}(T)$ for reaction over a saddle point were calculated using multi-conformer canonical transition state theory (MC-CTST) in a rigid rotor harmonic oscillator (RRHO) approximation[17] using the M06-2X/aug-cc-pVQZ rovibrational characteristics and the (IRCMax) CCSD(T)/CBS(DTQ)//M06-2X energies, augmented with conformer-specific zero-curvature WKB tunneling corrections based on the CCSD(T)/aug-cc-pVTZ//M06-2X IRC energy profiles. In the case of k_{-a}, the partition function is optimized in the aforementioned E,J-resolved μVTST calculations. For any of the elementary rate coefficient k_{-a}, k_{b1} and k_{b2} we have then the following expression:

$$k_{MC-TST}(T) = \frac{kT}{h}\frac{\sum_i \kappa_i \cdot Q_i^{\neq}(T)exp\left(\frac{-E_i}{kT}\right)}{\sum_i Q_i^{complex}(T)exp\left(\frac{-E_i}{kT}\right)}exp\left(\frac{-E_b}{kT}\right)$$

where Q_i are the conformer-specific partition functions for conformer i at energy E_i relative to the lowest conformer, and E_b is the energy difference between the complex ground state and the lowest TS conformer. The WKB tunneling corrections k_i are only calculated explicitly for the dominant conformers (see above); the remaining conformers are assigned averaged tunneling corrections. Note that in the $k_{HPL}(T)$ expression, the partition function and relative energy of the complex cancels out. The rate coefficient near room temperature is strongly influenced by redissociation of the complex back to the separated reactants. At these intermediate temperatures, a fair approximation would have been to assume an equilibrium between free reactants and the complex, describing the kinetics as a set of direct reactions from free reactants to the H-abstraction transition state. The lifetime of the complex near room temperature is comparatively short, ~10^{-11} s, and at atmospheric pressures the rate coefficient might be influenced by pressure; as discussed in Ref. [16], the influence is not expected to be significant and is thus not considered here.

b. The site-specific rate coefficients for methanediol + OH

The methanediol + OH reaction is similar to the CH_3OH + OH reaction[16, 18, 19], with two main channels of attack, i.e. H-abstraction from the carbon atom or the oxygen atoms. An S_N2-type substitution mechanism forming methanetriol was found to have a large barrier (see SI Tab. 2) and can be neglected.

Methanediol has been amply described in the literature (e.g., Refs [20-22]) and needs not to be discussed in detail. The main feature is the ground state of C_2 symmetry, of which two enantiomers exist. Geometries with other orientations of the hydroxyl H-atoms have significantly higher energies, with contributions to the room temperature population of only a few percent. Several different pre-reaction complexes of methanediol and OH exist; in our kinetic analysis we are not sensitive to the exact stability or state density of these complexes, as these parameters mostly cancel out of the kinetic equations in the high-pressure limit (see above).

Supplementary Information Table 2 | Relative energies (E_{rel} / kcal mol^{-1}) of the reactants, pre-reactive complex, and transition states for the CH_3OH + OH elementary reaction. Unless indicated otherwise, the energies are at the CCSD(T)/CBS(DTQ)//IRCMax(CCSD(T)/aug-cc-pVTZ//M06-2X/aug-cc-pVQZ) level of theory. CH_3OH + OH data are from Ref. [16]; CH_4 + OH data are from Ref. [23].

Compound		Conformer	E_{rel}
$CH_2(OH)_2$ + OH		most stable	0.00
Complex $CH_2(OH)_2$ --- OH		most stable	-5.22
TS $CH_2(OH)_2$ + OH → $C^{\bullet}H(OH)_2$ + H_2O	(R1a)	*ppl*	0.81
		tmt	1.50
		ptm	1.63
		mmc	1.71[a]
		mtp	3.07[a]
TS $CH_2(OH)_2$ + OH → $CH_2(OH)O^{\bullet}$ + H_2O	(R1b)	*mmt*	3.03
		mtt	4.75[b]
		mtl	5.00[b]
		tmm	5.07[b]
		tpl	5.45[b]
TS $CH_2(OH)_2$ + OH → $CH(OH)_3$ + H	(R1c)		44.04
CH_3OH + OH			0.00
TS CH_3OH + OH → $C^{\bullet}H_2OH$ + H_2O		most stable	0.98
TS CH_3OH + OH → $CH_3O^{\bullet}$ + H_2O		most stable	3.13
CH_4 + OH			0.00
TS CH_4 + OH → $C^{\bullet}H_3$ + H_2O			6.22[c]

[a] estimated from the CCSD(T)/CBS(DTQ)//M06-2X/aug-cc-pVQZ energies and the average impact of applying IRCMax refinements on the barrier height.
[b] derived from M06-2X/aug-cc-pVQZ energies relative to the most stable TS conformer mmt.
[c] W1 energy[23].

For each of the abstraction transition states, we located multiple conformers, where 3 out of 5 non-symmetry-related geometries contribute strongly (~90% at 300 K) to the C-H abstraction TS, and 1 geometry out of 5 (~80% at 300 K) to the O-H abstraction TS (see SI Tab. 2). Interestingly, according to our calculations, having two hydroxyl groups on the carbon does not lower the barrier height significantly compared to CH_3OH + OH, whereas the single hydroxyl group in methanol causes the barrier height of CH_3OH + OH to be significantly reduced compared to CH_4 + OH (SI Tab. 2). The pathways through the distinct TS conformers do not necessarily connect directly to the most stable pre-reaction or post-reaction complex geometry; this can affect the predicted rate coefficient by limiting the energy range accessible for tunneling for each particular TS conformer. As indicated above, we calculate conformer-specific tunneling based on explicit IRC pathway calculations of the dominant conformers, to account for these differences.

The obtained temperature-dependent effective rate coefficient and product distribution is listed in SI Tab. 3, and plotted in SI Fig. 4. The theoretical data favors the lower end of the rate coefficient range of the experimental analysis (see SI Sect. 1). Indeed, the calculated rate coefficient for the methanediol + OH reaction is about a factor of 7 lower than what is obtained by fitting the observed concentrations in the SAPHIR experiments. Accounting for the uncertainty on barrier height, tunneling, and state density suggests an uncertainty on the theoretical predictions of a factor 3; for comparison, our analogous analysis of the CH_3OH + OH reaction was able to reproduce the best available experimental data within a factor of 1.6 across the 20-400 K temperature range. Despite the apparent large difference in k(T) value, experiment and theory have thus overlapping uncertainty intervals. However, we cannot exclude that part of the difference is caused by effects not included in the theoretical analysis, such as complexation with water, or catalysis by available co-reactants or the chamber wall.

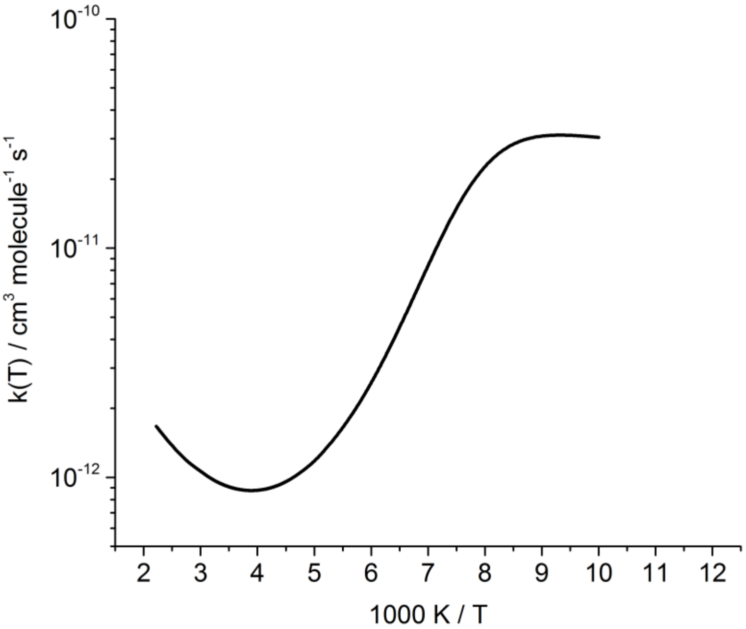

Supplementary Information Figure 4 | Theoretically predicted rate coefficient k(T) for the $CH_2(OH)_2$ + OH reaction.

Supplementary Information Table 3 | Theoretically predicted rate coefficient k(T), and product distribution Y(T), for the $CH_2(OH)_2$ + OH reaction.

T / K	k(T) / $cm^3\ s^{-1}$	Y($C^{\bullet}H(OH)_2$)	Y($CH_2(OH)O^{\bullet}$)
150	5.50×10^{-12}	0.61	0.39
160	3.35×10^{-12}	0.70	0.30
170	2.28×10^{-12}	0.77	0.23
180	1.71×10^{-12}	0.82	0.18
190	1.38×10^{-12}	0.86	0.14
200	1.17×10^{-12}	0.88	0.12
210	1.05×10^{-12}	0.90	0.10
220	9.68×10^{-13}	0.92	0.08
230	9.18×10^{-13}	0.93	0.07
240	8.90×10^{-13}	0.94	0.06
250	8.77×10^{-13}	0.94	0.06
260	8.75×10^{-13}	0.95	0.05
270	8.83×10^{-13}	0.95	0.05
280	8.97×10^{-13}	0.96	0.04
290	9.18×10^{-13}	0.96	0.04
300	9.44×10^{-13}	0.96	0.04
310	9.74×10^{-13}	0.96	0.04
320	1.01×10^{-12}	0.96	0.04
330	1.05×10^{-12}	0.96	0.04
340	1.09×10^{-12}	0.97	0.03
350	1.13×10^{-12}	0.97	0.03
360	1.17×10^{-12}	0.97	0.03
370	1.22×10^{-12}	0.97	0.03
380	1.27×10^{-12}	0.97	0.03
390	1.32×10^{-12}	0.97	0.03
400	1.38×10^{-12}	0.97	0.03
410	1.43×10^{-12}	0.97	0.03
420	1.49×10^{-12}	0.97	0.03
430	1.55×10^{-12}	0.97	0.03
440	1.61×10^{-12}	0.97	0.03
450	1.67×10^{-12}	0.97	0.03
460	1.74×10^{-12}	0.97	0.03
470	1.80×10^{-12}	0.97	0.03
480	1.86×10^{-12}	0.97	0.03
490	1.93×10^{-12}	0.97	0.03
500	2.00×10^{-12}	0.97	0.03

c. The products of the methanediol + OH reaction in the atmosphere

As discussed above, the $CH_2(OH)_2$ + OH reaction has two accessible channels: H-abstraction from the carbon atom, or from an oxygen atom. In this section, we present a set of theoretical calculations looking at the subsequent reactions of the products of these elementary reactions, showing that the only product formed in non-negligible quantities is formic acid (HCOOH), with coproduct HO_2, though this does not imply a direct reaction. The dominant product formation in atmospheric conditions then follows the following reaction scheme:

$CH_2(OH)_2 + OH \rightarrow {}^{\bullet}CH(OH)_2 + H_2O$

${}^{\bullet}CH(OH)_2 + O_2 \rightarrow {}^{\bullet}OOCH(OH)_2$

${}^{\bullet}OOCH(OH)_2 \rightarrow HCOOH + HO_2$

All energies discussed in this section were calculated at the CCSD(T)/CBS(DTQ) // M06-2X/aug-cc-pVQZ level of theory, unless indicated otherwise.

i. *After H-abstraction from the carbon atom*

The initiation reaction with the lowest barrier is abstraction from the carbon atom, with a predicted barrier of 0.81 kcal mol^{-1} :

$CH_2(OH)_2 + OH \rightarrow {}^{\bullet}CH(OH)_2 + H_2O$ (R1a)

For the dihydroxy-methyl radical formed, $C^{\bullet}H(OH)_2$, nine reactions were examined, listed in SI Tab. 4. Most of these reactions have either a high barrier to reaction, or are strongly endothermic, making these reactions negligible even for chemically activated $C^{\bullet}H(OH)_2$ possibly formed in reaction R1a. Under atmospheric conditions, with a partial pressure of O_2 of ~0.2 bar, the dominant loss reaction will then be direct H-abstraction, forming HCOOH + HO_2, or formation of dihydroxymethylperoxy, ${}^{\bullet}OOCH(OH)_2$, through a near-barrierless reaction path.

The formation of ${}^{\bullet}OOCH(OH)_2$ is highly exothermic, enabling fast unimolecular decomposition of the peroxy radical to HCOOH + HO_2. The mechanism is identical to that of other α-hydroxy alkyl radicals + O_2, whose chemistry has been described in detail[24-35]. The potential energy surface for ${}^{\bullet}OOCH(OH)_2$ is shown in SI Fig. 5, where we find that energetics are similar to those predicted by Ref. [33] for a range of α-hydroxy peroxy radicals, except that the complex of formic acid with HO_2 is significantly stronger than for carbonyl compounds, as expected, and that the formic acid product is somewhat more stable than the carbonyl compounds formed from mono-hydroxylated peroxy radicals. It should be noted that the lifetime of the chemically activated ${}^{\bullet}OOCH(OH)_2$ formed in the $C^{\bullet}H(OH)_2 + O_2$ reaction is too short to allow bimolecular reactions. Technically, formic acid and HO_2 establish an equilibrium with their complex and with the ${}^{\bullet}OOCH(OH)_2$ radicals, and thus enable bimolecular reactions of ${}^{\bullet}OOCH(OH)_2$; this equilibrium is shifted strongly to the free fragments, however, and will not have appreciable impact except at very low temperatures such as those found near the tropopause.

Supplementary Information Table 4 | Barrier heights and reaction energies for reactions of importance in the product formation in the methanediol + OH system.

Reaction		E_b / kcal mol^{-1}	E_{react} / kcal mol^{-1}
$C^{\bullet}H(OH)_2 + O_2 \rightarrow {}^{\bullet}OOCH(OH_2)$	(R2)	[low][a]	-39.4
$C^{\bullet}H(OH)_2 + O_2 \rightarrow HCOOH + HO_2$	(R3)	3.6[b]	-47.3
$C^{\bullet}H(OH)_2 \rightarrow HCOOH + H$	(R4)	24.8	10.3
$C^{\bullet}H(OH)_2 \rightarrow {}^{\bullet}OCH_2OH$	(R5)	34.1	9.3
$C^{\bullet}H(OH)_2 + H_2O \rightarrow CH_2(OH)_2 + OH$	(R-1a)		22.7
$C^{\bullet}H(OH)_2 \rightarrow {}^3C(OH)_2 + H$	(R6)		110.8
$C^{\bullet}H(OH)_2 \rightarrow {}^1C(OH)_2 + H$	(R7)		52.0
$C^{\bullet}H(OH)_2 \rightarrow {}^3CHOH + OH$	(R8)		108.5
$C^{\bullet}H(OH)_2 \rightarrow {}^1CHOH + OH$	(R9)		87.5

[a] No saddle point was located for this channel; the reaction is assumed to be near-barrierless, by analogy with other alkyl + O_2 addition reactions

[b] Based on IRCMax(CCSD(T)/aug-cc-pVTZ//M06-2X/aug-cc-pVQZ) geometries. The TS for direct H-abstraction has a very high multi-reference characters (T1 diagnostic > 0.06) and the result should be considered unreliable.

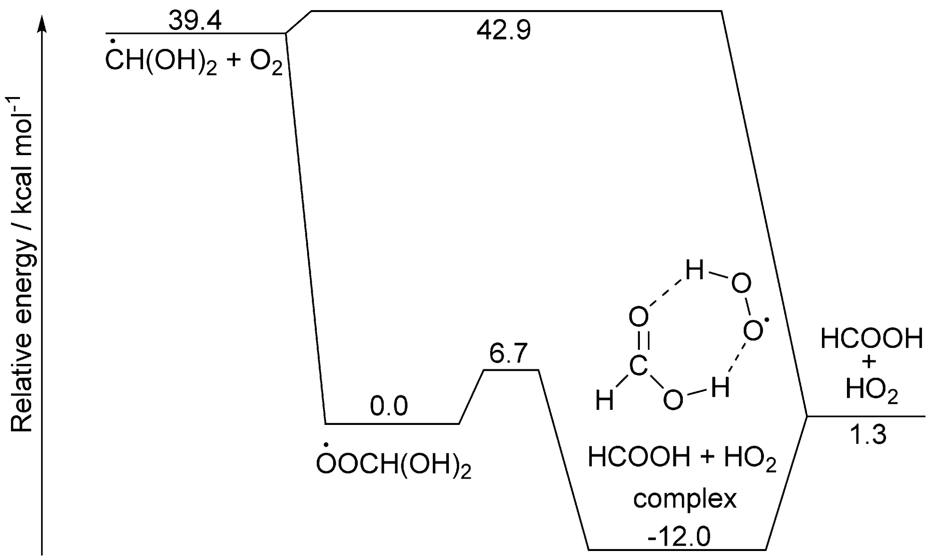

Supplementary Information Figure 5 | ZPE-corrected potential energy surface for the dihydroxymethyl + O_2 reaction.

ii. After H-abstraction from the oxygen atom

The initiation reaction where an H-atom is abstracted from methanediol has a predicted energy barrier of 3.03 kcal mol^{-1}:

$CH_2(OH)_2 + OH \rightarrow {}^{\bullet}OCH_2OH + H_2O$ (R1b)

For the dihydroxy-methyl radical formed, $CH_2(OH)O^{\bullet}$, four reactions were examined, listed in SI Table 5. Most of these reactions have either a sizable barrier to reaction, or are strongly endothermic, making these reactions less important even for chemically activated ${}^{\bullet}OCH_2OH$ possibly formed in reaction R1b. Under atmospheric conditions, with a partial pressure of O_2 of ~0.2 bar, the dominant loss reaction will be H-abstraction by O_2, with an energy barrier predicted to be below 6 kcal mol^{-1}. Direct unimolecular H-atom elimination can not be fully excluded, especially if highly energized hydroxymethoxy radicals are formed, but under atmospheric conditions this will lead to the same final products, HCOOH + HO_2.

Supplementary Information Table 5 | Reactions of the hydroxymethoxy radical formed from methanediol + OH.

Reaction		E_b / kcal mol^{-1}	E_{react} / kcal mol^{-1}
${}^{\bullet}OCH_2OH + O_2 \rightarrow HCOOH + HO_2$	(R10)	5.93	-47.3
${}^{\bullet}OCH_2OH \rightarrow HCOOH + H$	(R11)	12.0	1.1
${}^{\bullet}OCH_2OH \rightarrow HCHO + OH$	(R12)		22.0
${}^{\bullet}OCH_2OH \rightarrow C^{\bullet}H(OH)_2$	(R-5)		24.9

3. Model simulations

a. *BASE simulation*

i. *Gas-phase kinetics*

The chemical mechanism including the oxidation of organic compounds is implemented in the submodel MECCA[36] and referred to as the Mainz Organic Mechanism (MOM)[37]. It is available in the EMAC v2.53.0 release[38]. The employed configuration does not represent formation of Secondary Organic Aerosol (SOA). However, SOA formation from isoprene is approximated by assuming that the fraction of isoprene hydroperoxides (ISOPOOH) which does not yield epoxydiols (IEPOX) upon reaction with OH is removed. This is consistent with Ref. [39]. SOA formation via reactive uptake of IEPOX and cloud processing of dicarbonyls is currently neglected. Representation of these processes could significantly lower the formic acid (HCOOH) yield from isoprene oxidation both directly and indirectly.

Formic acid production in MOM takes place via four pathways:

a) Ozonolysis of unsaturated organics, primary and secondary, which yields the stabilized Criegee Intermediate (CH_2OO) that mainly forms hydroxymethyl hydroperoxide ($HOCH_2OOH$) upon reaction with the water monomer and dimer, which then degrades to HCOOH following:

$HOCH_2OOH + OH \rightarrow OH + H_2O + HCOOH$ $k = 2.86 \times 10^{-11}$ cm^3 molec^{-1} s^{-1}

$HOCH_2OOH + h\nu\ (+ O_2) \rightarrow OH + HO_2 + HCOOH$ J_{CH3OOH} s^{-1}

b) HO_2-initiated oxidation of formaldehyde (HCHO) at low-temperature according to the IUPAC recommendations (sheets HOx_VOC58 and HOx_VOC59) for the initial steps:

$HCHO + HO_2 \rightarrow HOCH_2O_2$ $k = 9.7 \times 10^{-15} \exp(625/T)$ cm^3 molec^{-1} s^{-1}

$HOCH_2O_2 \rightarrow HCHO + HO_2$ $k = 2.4 \times 10^{12} \exp(-7000/T)$ s^{-1}

c) OH-initiated oxidation of acetylene (HC≡CH) according to Table 2-1 (D4) of JPL recommendations:

$HC{\equiv}CH + OH \rightarrow CO + HO_2 + HCOOH$ $k_o^{300} = 5.5 \times 10^{-30}$, n = 0, $k_{inf}^{300} = 8.3 \times 10^{-13}$, m = 2., F_c=0.6

d) OH-initiated oxidation of vinyl alcohol ($H_2C{=}CHOH$) according to Ref. [40]

$H_2C{=}CHOH + OH \rightarrow CO + HO_2 + HCOOH$ $k = 4.3 \times 10^{-11}$ cm^3 molec^{-1} s^{-1}

Vinyl alcohol is mainly produced by the photo-induced keto-enol tautomerization of acetaldehyde (CH_3CHO)[41, 42]

$CH_3CHO + h\nu \rightarrow H_2C{=}CHOH$

Other sources of vinyl alcohol are photolysis of *n*-butanal and pyruvic acid according to Ref. [43]. The theoretical catalytic effect of formic acid in the conversion between acetaldehyde and vinyl alcohol is implemented according to Ref. [44].

The speculative formic acid production from enols larger than vinyl alcohol is not included in MOM. In particular, MOM as well as MCM v3.3.1 contains the species HMAC and HVMK, which are enols. MOM consequently has formic acid production from their reaction with OH. However, since the production of HMAC and HVMK from isoprene is speculative, the respective reaction products assigned by MCM v3.3.1 are used here.

ii. Emissions

Biogenic emissions of organics like isoprene have a strong influence on the atmospheric composition but are rather uncertain[45]. Despite the overall underestimate of formic acid in the troposphere, the standard EMAC model with the MEGAN2 emission algorithm[46] produces 591 Tg yr^{-1} of isoprene leading to an overestimate of the formic acid total column over the Amazon. The implementation of the emission algorithm lacks the known reduction effect of soil moisture stress which is estimated to reduce isoprene emissions by as much as 50%[45]. Moreover, the EMAC model tends to overestimate the 2m temperature over the Amazon which lacks a realistic soil moisture memory effect due to its simple "bucket model" for the soil water. This leads to a significant overestimate of isoprene emissions which are strongly sensitive to light and temperature[47]. Given the uncertainties and issues described above, we reduced the isoprene emission factors by 50% and obtain a global isoprene emission of 382 Tg yr^{-1} similar to the estimate obtained by Ref. [45] when soil moisture stress is accounted for.

The surface fluxes of formic acid are generally considered bi-directional with large upward fluxes occasionally inferred[48, 49]. MEGAN2 produces a small flux that is lower than 3 Tg yr^{-1}. The corresponding emission factors are very uncertain[46]. Since we aim to an explicit representation of sources and sinks of formic acid, we turned off such emissions.

iii. Scavenging and dry deposition

The equilibrium between the gas- and aqueous phase for formic acid is governed by its intrinsic Henry's law constant (H_{HCOOH}=8.9 × 10^3 M atm^{-1}) and acidity constant (K_a = 1.8 × 10^{-4} M) which are used in the scavenging routines. The effective Henry's law constants (H*) for acids are calculated according to the formula:

$$H^* = H \times (1 + K_a / [H^+])$$

For dry deposition, pH values of 7 and 8.14 for the leaf mesophyll and ocean water have been assumed, respectively. The corresponding H* are calculated to be 1.6 × 10^7 and 2.2 × 10^8 M atm^{-1}, respectively. The surface reactivity parameter is set to 0. The scavenging of formaldehyde is computed with the H* of 3.2 × 10^3 M atm^{-1}, which takes into account the equilibrium with methanediol[50].

The standard EMAC model has been modified with respect to the problematic representation of surface exchanges over the Amazon forest. In the dry season (September – November), the modelled soil moisture stress for stomatal conductance unrealistically shuts down stomatal dry deposition of trace gases. As a result, levels of ozone and oxygenated organics are significantly overestimated. This model deficiency also affects the evapotranspiration leading to positive temperature biases[47]. Improving the hydrological model is beyond the scope of this study. Moreover, also under non-limiting drought conditions, the modelled dry deposition

fluxes of major oxygenated organics is far below what is measured in the field. An example is hydroxymethyl hydroperoxide which is a main product of VOC ozonolysis and an important precursor of formic acid. Our modelled maximum deposition velocities are less than 2 cm s^{-1} while the measured ones reach up to 5 cm s^{-1} (see Ref. [51]). Thus, a series of changes to the EMAC dry deposition algorithm has been implemented and is detailed below.

The dry deposition scheme implemented in EMAC by Ref. [52] has been modified. The scheme effectively represents only stomatal deposition without meterological controls by vapor pressure deficit and heat stress. The latters have been introduced as factors multiplying the stomatal conductance according to Refs [53, 54], respectively. The soil moisture stress factor which reduces stomatal conductance, and thus stomatal deposition, depends on a critical and wilting point as in second-generation hydrological models. However, the hydrological model implemented in ECHAM5 is essentially a "bucket" model and the original formula for the soil moisture stress factor by Ref. [55] has been re-introduced. This makes the stress factor assume more realistic values and slightly ameliorates the ECHAM5 problem of the too dry Amazon in the dry season. Moreover, the parameterization for the non-stomatal deposition under dry and wet conditions by Ref. [56] has been implemented. Finally, for the major oxygenated C_1-C_5 organics artificially high effective Henry's law coefficients have been implemented similarly to Ref. [57] in order to produce realistic deposition velocities like the ones reported by Ref. [51].

Over the ocean dry deposition of formic acid is treated by a two-layer model fully described by Ref. [58]. As concentration in the ocean surface layer we took the average lower bound concentration of 500 nmol L^{-1} of formate measured by Ref. [59]. At pH = 8.14 the corresponding formic acid concentration must be 2 nmol L^{-1}.

b. DIOH and DIOL simulations

i. Gas-phase kinetics

Methanediol ($HOCH_2OH$) in the gas phase reacts with OH as below

$HOCH_2OH + OH (+ O_2) \rightarrow HO_2 + H_2O + HCOOH$ $\qquad k = 7.5 \times 10^{-12}\ cm^3\ molec^{-1}\ s^{-1}$

The rate constant is from the best fit of the box model results to the SAPHIR experimental time series of formic acid and methanediol (see SI Sect. 1.b). This is consistent with the independent determination of the rate constant using the OH reactivity measurements (see SI Sect. 1.c), but outside the range of values from the theoretical predictions (see SI Sect. 2.b). A more accurate determination of this rate constant is needed.

ii. Aqueous-phase kinetics

The temperature-dependent equilibrium is considered explicitly in the aqueous-phase chemical mechanism as:

$HCHO_{(aq)} (+ H_2O) \rightarrow HOCH_2OH_{(aq)}$ $\qquad k_h = 2.04 \times 10^5 \exp(-2936/T)\ s^{-1}$

$HOCH_2OH_{(aq)} \rightarrow HCHO_{(aq)} (+ H_2O)$ $\qquad k_d = 4.96 \times 10^7 \exp(-6705/T)\ s^{-1}$

In order to account for the kinetically-limited dehydration and outgassing of aqueous-phase methanediol, in the reaction

$HCHO_{(aq)} + OH_{(aq)} \rightarrow HCOOH_{(aq)} + HO_{2(aq)}$

$HCHO_{(aq)}$ has been replaced by $HOCH_2OH_{(aq)}$.

The temperature range where most of the cloud-mediated formic acid production occurs is 260-300 K. However, the kinetic data used here for the hydration/dehydration of methanediol are derived from experiments in the 293-333 K range. Furthermore, it is known that acid catalysis of methanediol dehydration is important for solution pH < 3.5 (see Ref. [60, 61]). This is outside the typical pH range of cloud droplets which is entirely covered (pH 5-7) by the kinetic data we use in this study[62]. Specifically, Ref. [62] showed that the dehydration constant of methanediol did not change significantly (<13%) for the pH 6-7.5 range.

The full kinetic model is detailed in SI Tab. 6.

Supplementary Information Table 6 | The kinetic model for the aqueous-phase oxidation of methanediol. k_arr is the rate constant function A * exp(B*(1/temp-1/298.15)). In blue are the new reactions added for the DIOH and DIOL simulations.

Reaction		Rate constant $[M^{-1}s^{-1}]$ or $[s^{-1}]$	Reference
HCHO + OH	→ $HCOOH + HO_2$	k_arr(7.7E8,-1020.,temp)	Ref. [63]
HCHO {+ H_2O}	→ $HOCH_2OH$	2.04E5*EXP(-2936./temp)	Ref. [62]
$HOCH_2OH$	→ HCHO	4.96E7*EXP(-6705./temp)	Ref. [62]
$HOCH_2OH$ + OH	→ $HCOOH + HO_2$	k_arr(7.7E8,-1020.,temp)	Ref. [63]
$HOCH_2OH + CO_3$	→ HCO_3^-+ $HCOOH + HO_2$	1.3E4	Ref. [64]
$HOCH_2OH + NO_3$	→ NO_3^- + H^+ + $HCOOH + HO_2$	k_arr(1.E6,-4500.,temp)	Ref. [65]
$HOCH_2OH + SO_4^-$	→ SO_4^{2-} + $HCOOH + HO_2$	k_arr(1.4E7,-1300.,temp)	Ref. [66]
$HOCH_2OOH$ + OH	→ HCOOH + OH	6.3E8*0.20	Ref. [67]
$HOCH_2OOH$ + OH	→ $HCHO + HO_2$	6.3E8*0.80	Ref. [67]
HCOOH + OH	→ $HO_2 + CO_2$	k_arr(1.1E8, -991.,temp)	Ref. [63]
$HCOOH + NO_3$	→ NO_3^-+ Hp + $HO_2 + CO_2$	k_arr(3.8E5,-3400.,temp)	Ref. [65]
$HCOOH + SO_4^-$	→ SO_4^{2-} + H^+ + $HO_2 + CO_2$	k_arr(1.7E8,-1500.,temp)	Ref. [68]
$HCOO^-$ + OH	→ $H_2O + O_2^- + CO_2$	k_arr(3.1E9,-1240.,temp)	Ref. [63]
$HCOO^- + NO_3$	→ NO_3^- + H^+ + O_2^- + CO_2	k_arr(5.1E7,-2200.,temp)	Ref. [65]
$HCOO^- + SO_4^-$	→ SO_4^{2-} + $HO_2 + CO_2$	k_arr(1.7E8,-1500.,temp)	Ref. [68]
$HCOO^- + SO_5^-$	→ HSO_5^- + O_2^- + CO_2	k_arr(1.4E4,-4000.,temp)	Ref. [68]
$HCOO^- + CO_3^-$	→ 2 HCO_3^- + HO_2	1.5E5	Ref. [64]
$HCOO^- + O_3$	→ OH + O_2^- + CO_2	1.E2	Ref. [69]

iii. Henry's law constants

The intrinsic Henry's law constant for formaldehyde is used (H_{HCHO}=2.53 M atm^{-1}). This is derived from the H* used in Ref. [70] and the hydration equilibrium constant at 298 K by Ref. [62] K_{hyd}= 1.28×10^3 knowing that the relationship between H* and H for carbonyls is calculated according to the formula:

$$H^* = H \times (1 + K_{hyd})$$

The Henry's law constant of methanediol ($H_{HOCH2OH}$) has not been measured at any temperature. Moreover, estimation methods bear inherent uncertainties. The HENRYWIN v3.10 software from the EPI suite provides estimates with three methods ranging from 0.25 to 9.3×10^4 M atm^{-1} at 298 K (i.e., spanning almost two orders of magnitude). For C_1-C_2 alcohols

and hydroperoxides (including hydroxymethyl hydroperoxide), the HENRYWIN estimates with the bond method are within a factor of 3 of the experimental data, whereas for the small glycol 1,2-ethanediol, the estimate differs from the experimental values by more than an order of magnitude. For such similar compounds, the temperature dependence of the Henry's law constant suggests that this latter could be roughly one order of magnitude higher at typical temperatures of warm clouds. Nevertheless, this remains uncertain as measurements at temperatures down to ~248 K indicate that in supercooled water (such as in the clouds) the solubility of some VOCs decreases with the temperature as much as it increases between 273 K and 298 K due to ice-like clusters expelling the VOCs to the gas phase[71]. Therefore, to gauge the impact of all these uncertainties on the results, two EMAC simulations have been performed with $H_{HOCH2OH}=10^6$ M atm^{-1} (EMAC$_{(dioh)}$) and $H_{HOCH2OH}=10^4$ M atm^{-1} (EMAC$_{(diol)}$). The latter value is the intermediate estimate employing the bond method by HENRYWIN v3.10 from the EPI suite, whereas the former ($H_{HOCH2OH}=10^6$ M atm^{-1}) has been set two orders of magnitude higher. This interval includes both an uncertainty on HENRYWIN predictions, set here at one order of magnitude, and the possible temperature dependence of the methanediol solubility, for which no constraint exists. The extra formic acid that is predicted by EMAC$_{(dioh)}$ and EMAC$_{(diol)}$ compared to the reference simulation provide, respectively, a lower and a higher estimate of the additional formic acid that is produced via the multiphase processing of formaldehyde. However, if we assume that the solubility of methanediol at 298 K is similar to the one of hydroxymethyl hydroperoxide (~10^6 M atm^{-1}), methanediol solubility could be as high as ~10^7 M atm^{-1} at typical temperatures of warm clouds.

For the dry deposition calculations, an effective H of 10^6 M atm^{-1} for methanediol has been used similarly to the one needed to reproduce deposition fluxes of hydroxymethyl hydroperoxide as reported by Ref. [51].

4. Methanediol measurements

The unexpectedly large formic acid amount resulting from the cloud processing pathway raises the question why significant concentrations of a simple molecule like methanediol could elude detection and identification. Many chamber studies in the past have used the Hantzsch method to detect formaldehyde[72]. However, the method involves a liquid-phase derivatization of formaldehyde, which involves methanediol, and therefore cannot be distinguished from. On the other hand, in the last two decades field measurements of formaldehyde have been widely performed with proton-transfer-reaction mass spectrometry (PTR-MS). With PTR-MS instruments, the fragment mass-to-charge ratio (m/z) 31 (CH_3O+) is detected, for which interferences from other compounds have been identified and investigated[73-75]. Although methyl hydroperoxide and higher alcohols have been shown to be sources of m/z 31, the contribution of methanediol has never been considered. Alcohols, when protonated in the PTR-MS instruments, are well known to eliminate a water molecule and methanediol is expected to significantly contribute to m/z 31 in air masses with a cloud processing history. In fact, our chamber experiments yielded a PTR-MS signal at m/z 31 with a time profile that is a combination of the ones for formaldehyde (DOAS) and methanediol (Hantzsch - DOAS). This is also the case for the much weaker signal at m/z 49. Our model predictions with $EMAC_{(dioh)}$ and $EMAC_{(diol)}$ for the minimum and maximum interference of methanediol on field measurements of formaldehyde are given as the annual average $HOCH_2OH_{(g)}/HCHO_{(g)}$ ratio (SI Fig. 6).

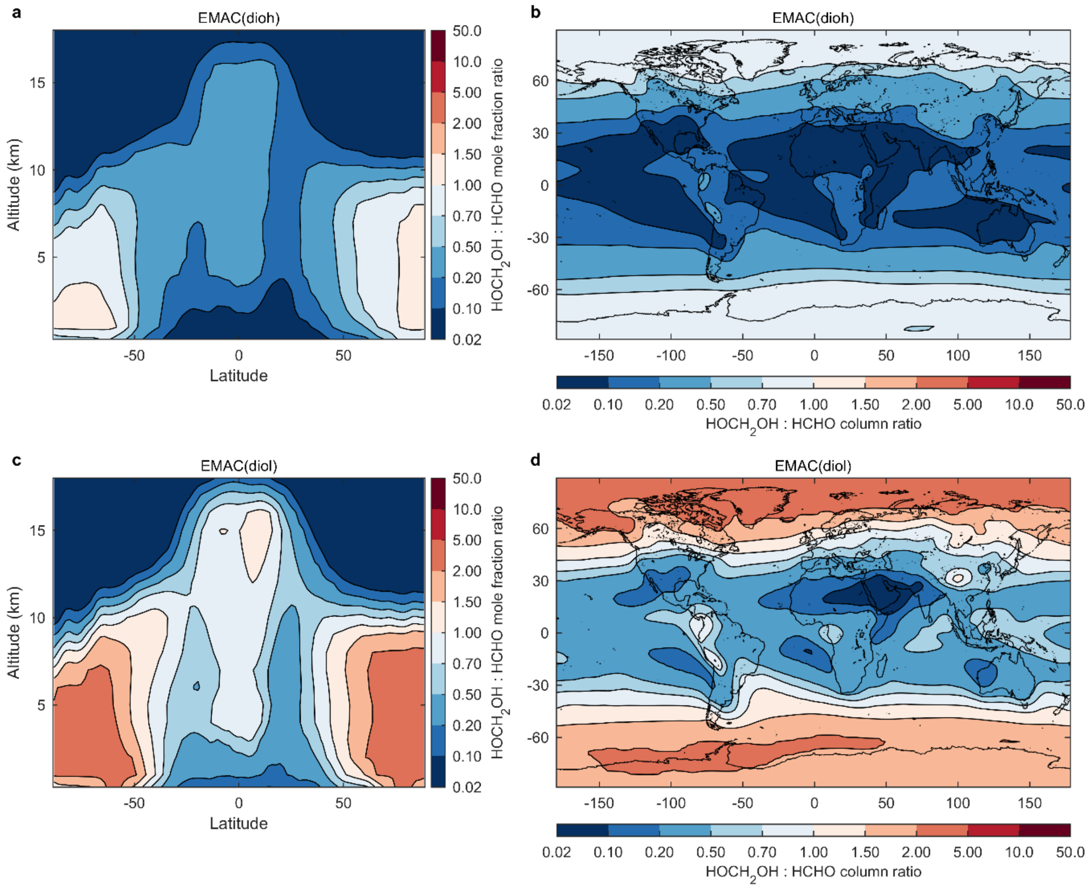

Supplementary Information Figure 6 | Methanediol-to-formaldehyde ratio from EMAC. a-d, Zonal mean of the $HOCH_2OH/HCHO$ ratio (**a,c**) and of the $HOCH_2OH/HCHO$ column ratio (**b,d**) predicted by the $EMAC_{(dioh)}$ (**top panels**) and $EMAC_{(diol)}$ (**bottom panels**) simulations, averaged over the 2010-2012 time period.

5. Remaining sources of uncertainties in model simulations

Uncertainties on the strength of the major pathways leading to formic acid remain. In this section we explore several reasons for the model-to-observation biases. Note that several points are already presented in the manuscript and that the effect of the uncertainties on the Henry's law constant of methanediol is discussed in SI Sect. 3.b.*iii*.

The total column overestimate of EMAC compared to IASI (Fig. 1; ED Figs 3,4) could suggest that VOC ozonolysis is overestimated over pristine tropical forests. Indeed, the isoprene oxidation scheme used in this study[37] may still under-predict the measured OH concentrations in such environments[76, 77] and overestimate the magnitude of VOC reactions with ozone. Inversion studies suggest a model overestimate of formaldehyde over tropical continental regions[78]. This is ascribed to a bias in the modelled isoprene emissions, although the uncertainty springing from the model VOC oxidation schemes is acknowledged. Our results indicate that the slow methanediol dehydration curbs formaldehyde outgassing, and thus leads to significantly lower formaldehyde levels, especially in remote environments.

The modelled distribution of clouds and cloud liquid water content is expected to significantly affect our estimates of the total methanediol being outgassed from cloud droplets. For instance, different convection parameterizations yield quite different cloudiness and precipitation patterns[79]. Furthermore, observational evidence of self-aggregation of convection systems has been presented and shown to lead to decreased low- and mid-level cloudiness, and to a drier free troposphere[80, 81]. A recent model study suggests that self-aggregation of convection could reduce the modelled cloud liquid water total column by as much as 15%[82]. This could also partly explained the $EMAC_{(diol)}$ over-prediction compared to the FTIR formic acid columns at remote sites.

Finally, the potential formation of unreactive water complexes of formic acid ($HCOOH{\cdot}H_2O$) and methanediol ($HOCH_2OH{\cdot}H_2O$) has to be considered. From theoretical kinetic data[83] it has indeed been suggested that up to 12% of formic acid can form this complex under tropospheric conditions frequent within the tropics (100% humidity and high ambient temperatures). Hydrogen bonding of water with the two hydroxyl groups could potentially favour the formation of a $HOCH_2OH{\cdot}H_2O$ complex stronger than $HCOOH{\cdot}H_2O$. Although the experimentally determined equilibrium constant is lacking, a recent theoretical determination suggest that $HOCH_2OH{\cdot}H_2O$ cannot be abundant[22].

The remaining negative biases of the model-to-IASI comparison, especially over Siberia and North China, could be further reduced by including a more advanced vegetative source accounting for high net upward fluxes of formic acid from the boreal ecosystem[48, 49]. The source from vinyl alcohol might be underestimated as models predict too low levels of its precursor acetaldehyde. Moreover, such biases could also be dampened by implementing the explicit methanediol dehydration kinetics in the aerosol chemical mechanism. Ref. [84] discussed the potential role of aerosol for formic acid production although focusing on a reactive uptake of OH on biomass burning aerosols. However, the aerosol-mediated pathway to formic acid we propose here is expected to have a lower impact than the cloud-mediated one on the global scale because of the much larger liquid water content in clouds.

6. Model comparison with FTIR measurements

The ground-based Fourier Transform InfraRed (FTIR) total column measurements used here have been derived from high-resolution (between 0.004 and 0.011 cm^{-1}) IR solar absorption spectra recorded regularly, under clear-sky conditions, at a suite of sites located at various latitudes (ED Fig. 1). Most of them are affiliated with the Network for the Detection of Atmospheric Composition Change (NDACC; http://www.ndacc.org)[85]. Vertical distributions of formic acid have been retrieved with either the PROFITT, SFIT-2 or SFIT-4 NDACC codes, all implementing the Optimal Estimation method of Ref. [86]. In all cases, the HITRAN 2008 spectroscopic compilation[87] has been adopted, complemented with pseudo-line parameters for heavy species provided by G.C. Toon (NASA-JPL, Pasadena, CA). Although the retrieval approaches may vary slightly from site to site, all strategies use a broad spectral fitting window encompassing the ν_6 *Q*-branch of formic acid at 1105 cm^{-1} as well as a supplemental window needed to properly fit HDO, a critical interference species in the main window (e.g., Refs [88-90]). The a priori vertical distributions for the target and interfering species correspond to averages of profiles deduced from the IRWG iteration 6 of WACCM[91] v4 (Whole Atmosphere Community Climate Model) model predictions for the 1980-2020 time period employing the REFC1.3 scenario as part of the CCMVal/CCMI, 2012 project. Typical Degree Of Freedom for Signal (DOFS) values of ~1 from the retrievals, and inspection of the averaging kernel matrices, indicate that limited vertical information is available from the FTIR measurements, with a sensitivity restricted to the troposphere. Typical random and systematic uncertainties affecting the retrieved total columns have been evaluated in previous studies; they are in the range of 11-13 and 15-18%, respectively. More information can be found in Ref. [92] and references therein.

The model outputs and FTIR observations presented here cover the 2010-2012 time period. Owing to the scarcity of FTIR observations at some sites, a regular sampling of FTIR measurements throughout the year is not always guaranteed and hence the representation of the typical seasonal cycle of formic acid abundance may be impacted. The model outputs used here consist of daily mean formic acid volume mixing ratio (VMR) profiles, saved at the pixel encompassing the FTIR station. To account for the vertical resolution and sensitivity of the FTIR retrievals, the individual VMR profiles simulated by EMAC are interpolated onto the NDACC standard vertical scheme adopted at each FTIR station, according to a mass conservative interpolation. They are then smoothed by applying the FTIR averaging kernels according to the formalism of Ref. [93] (except for Wollongong where no averaging kernels are available). The averaging kernels used to convolve the model outputs are seasonal averages over December – February, March – May, June – August and September – November, and over the 2010-2012 years, obtained from the individual FTIR retrievals. Application of the averaging kernels provides the model vertical distribution of formic acid as would be seen by each FTIR instrument. The EMAC total columns are eventually computed from these smoothed VMR profiles by using the corresponding regridded air density profiles simulated by the model.

Extended Data Fig. 1 presents the seasonal cycle of the formic acid total columns derived from the ground-based FTIR observations and EMAC simulations at nine sites. Similar to the comparison involving IASI, it highlights the global under-prediction of formic acid abundance by $EMAC_{(base)}$ compared to the FTIR measurements. The implementation of the kinetically-limited dehydration and outgassing of aqueous-phase methanediol ($EMAC_{(dioh\text{-}diol)}$) reconciles the model simulations with the FTIR observations at many sites. However, $EMAC_{(diol)}$ simulation, in particular, over-predicts the FTIR formic acid columns at the Jungfraujoch and Izana high-altitude station, whereas $EMAC_{(dioh\text{-}diol)}$, despite noticeable improvements, cannot completely reproduce the peaks of elevated formic acid columns observed at Toronto and Wollongong. Reasons for these remaining model-to-observation discrepancies are likely linked to the relatively coarse resolution of the model simulation, which dilutes the formic acid information within a grid box and therefore fails to reproduce local enhancements or steep topography. Other local sources of formic acid might also be missing in the model.

7. OMI column observations

The Ozone Monitoring Instrument (OMI) is a nadir-viewing imaging spectrometer launched onboard the Aura platform in July 2004 in a Sun-synchronous polar orbit crossing the Equator around 13:30 local time (in ascending mode). It measures the solar radiation backscattered by the Earth's atmosphere and surface over the 270-500 nm wavelength range with a spectral resolution of about 0.5 nm[94].

The OMI formaldehyde dataset used here[95] is a European product developed in the framework of the EU FP7-project Quality Assurance for Essential Climate Variables (QA4ECV). The aim of this project is to address the need for reliable and traceable quality information by providing a fully traceable quality assurance effort on all aspects of the retrieval algorithm. The OMI formaldehyde retrieval details are provided in the QA4ECV deliverable 4.2 (2016, http://www.qa4ecv.eu/sites/default/files/D4.2.pdf) and the data files (v1.1) are openly distributed via the QA4ECV website (http://www.qa4ecv.eu/ecv/hcho-p).

Formaldehyde tropospheric columns are retrieved using the DOAS method including two main steps: (1) the fit into the earthshine radiance of the formaldehyde absorption along the mean light path between the Sun and the satellite (the slant column), (2) a radiative transfer calculation of the mean light path in order to transform the slant column into a vertical column for each observation condition (the air mass factor). For weak absorbers such as formaldehyde, an additional step is performed, consisting in the normalization of the tropospheric column using as reference region the remote Pacific Ocean (the background correction). The final tropospheric column is therefore a differential column providing the amount of formaldehyde over the background value due to methane oxidation.

The OMI formaldehyde slant columns are retrieved in the 328.5-359 nm interval. A set of high-resolution cross-sections is used, among them the formaldehyde absorption cross sections of Ref. [96]. Air mass factors are calculated using radiative transfer simulations performed with the LIDORT code[97]. Clouds are treated in the independent pixel approximation. Observations with cloud fractions larger than 40% are filtered out. No explicit correction is applied for aerosols but the cloud correction scheme accounts for a large part of the aerosol scattering effect[98]. The monthly albedo climatology of Ref. [99] at 342 nm is used. Daily a priori formaldehyde vertical profiles are obtained from the TM5 model[100]. Total column averaging kernels are provided for each pixel, as well as an estimate of the random and systematic error budget. The random uncertainty is of the order of 8×10^{15} molecules cm^{-2} for an OMI pixel. The systematic error remaining on the monthly and regionally averaged column is comprised between 20 and 40%.

The OMI averaging kernels were considered throughout this study for the model-to-satellite comparisons (see ED Figs 5,6). In that framework, the EMAC vertical profiles of formaldehyde (in partial subcolumns), sampled at the time and location of the OMI measurements, were interpolated onto the OMI pressure grids and smoothed by applying the OMI total column averaging kernels. The interpolation/convolution of a model profile was performed successively for each individual OMI observations embedded in a given model grid box, on a daily basis. It resulted in model vertical distributions of formaldehyde as would be retrieved by the OMI sensor, taking into account its non-uniform vertical sensitivity to the formaldehyde vertical distribution which is different for every satellite observation. A daily mean formaldehyde total column was eventually obtained for this model grid box by averaging all the formaldehyde total columns computed from the smoothed model profiles.

8. Supplementary figures

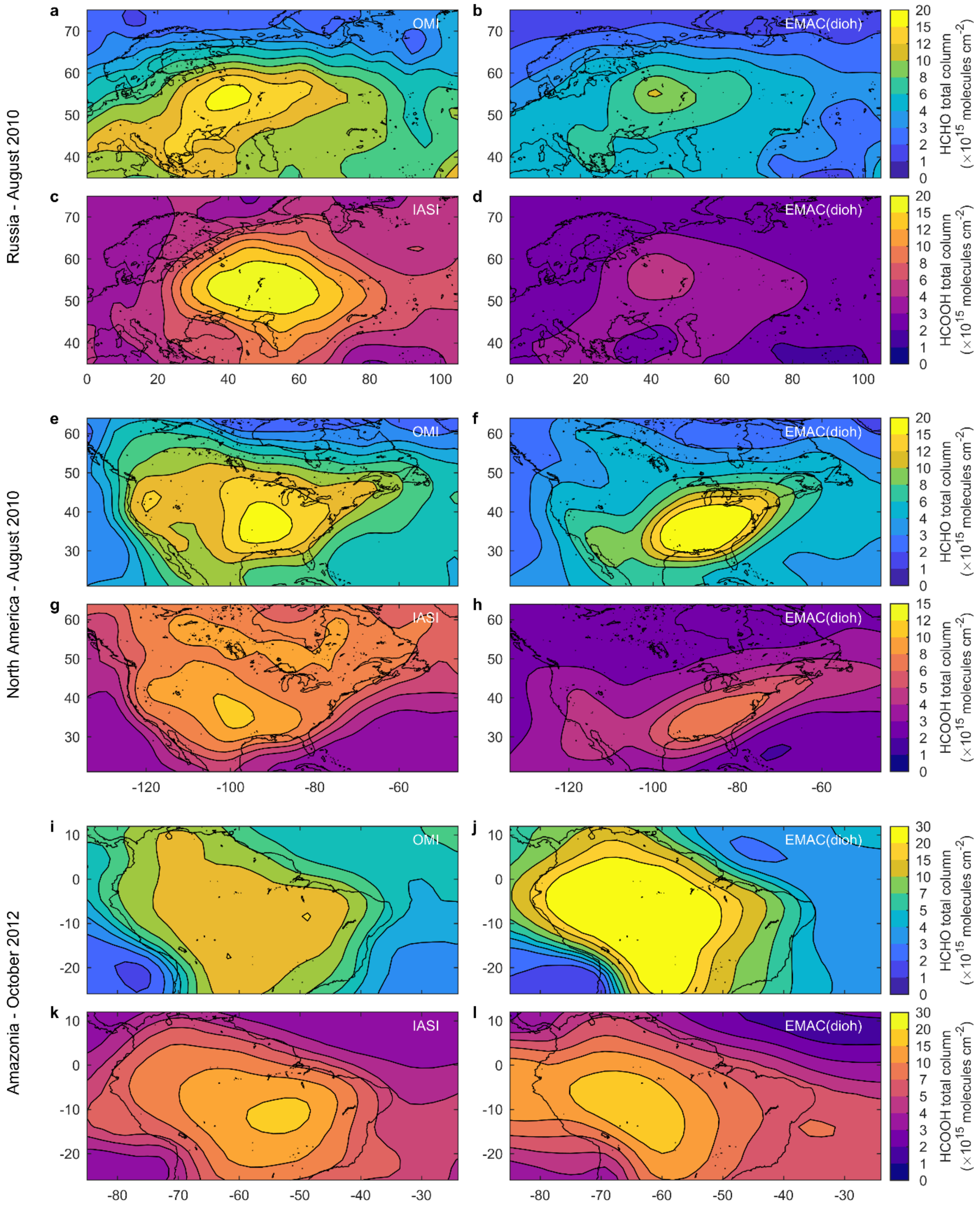

Supplementary Information Figure 7 | Impact of modelled formaldehyde biases on formic acid prediction. a-l, Monthly averaged formaldehyde (HCHO) and formic acid (HCOOH) columns (in 10^{15} molecules cm^{-2}) from IASI and OMI satellite measurements, respectively, and from the $EMAC_{(dioh)}$ simulation, over Russia in August 2010 (**a-d**), North America in August 2012 (**e-h**), and Amazonia in October 2010 (**i-l**). HCHO and HCOOH model data were sampled at the time and location of the OMI and IASI satellite measurements, respectively. The OMI averaging kernels were applied to the model profiles to account for the vertical sensitivity and resolution of OMI (IASI averaging kernels are not available).

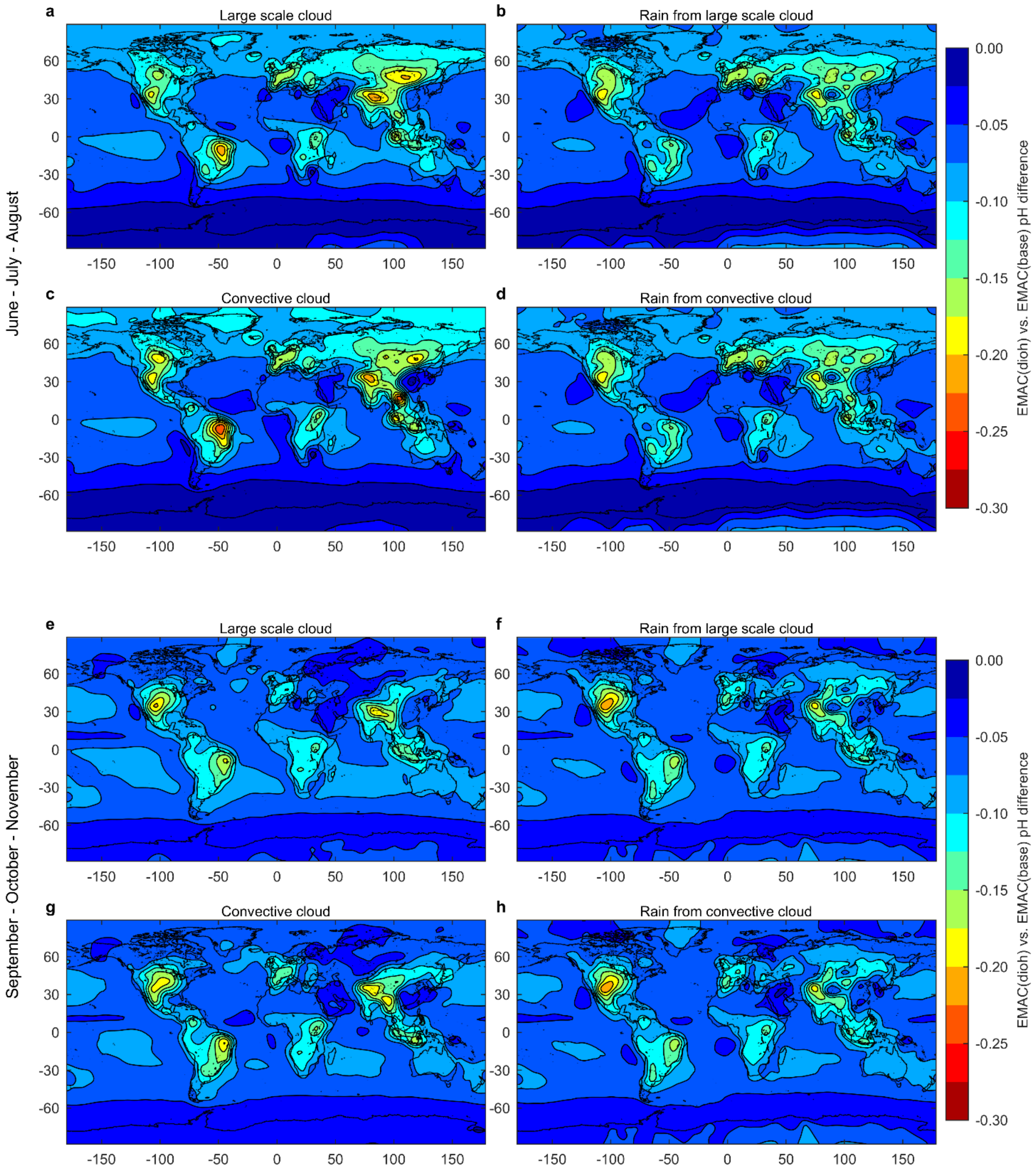

Supplementary Information Figure 8 | Impact of the cloud processing on cloud and rainwater acidity. a-h, pH difference of the large scale clouds (**a,e**) and associated rain (**b,f**), and of the convective clouds (**c,g**) and associated rain (**d,h**), between the $EMAC_{(dioh)}$ and $EMAC_{(base)}$ simulations. The pH differences presented here are seasonal averages over June – August (**a-d**) and September – November (**e-h**) 2010-2012. The pH decrease is due to the additional formic acid (HCOOH) production via the multiphase chemistry of methanediol implemented in $EMAC_{(dioh)}$.

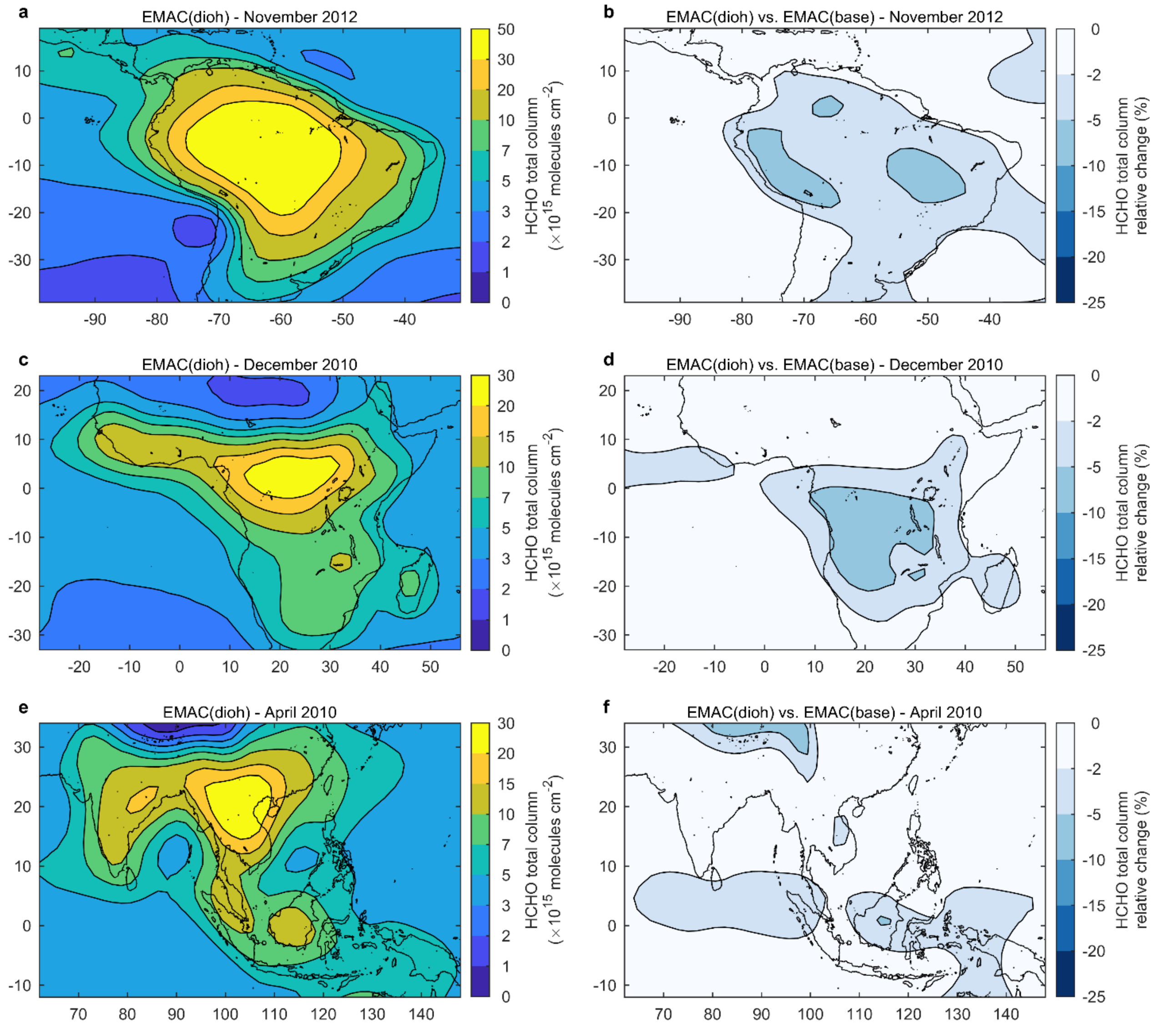

Supplementary Information Figure 9 | Impact of the cloud processing on formaldehyde modelling. Monthly averaged formaldehyde (HCHO) total column (in 10^{15} molecules cm^{-2}) simulated by the model implementing the multiphase chemistry of methanediol ($EMAC_{(dioh)}$; **left panels**), and relative difference of HCHO total column between $EMAC_{(dioh)}$ and the base version of the model ($EMAC_{(base)}$; **right panels**), over Amazonia in November 2012 (**a,b**), Central Africa in December 2010 (**c,d**) and Southeast Asia in April 2010 (**e,f**).

9. References

[1] Rohrer, F. *et al.* Characterisation of the photolytic HONO-source in the atmosphere simulation chamber SAPHIR. *Atmospheric Chemistry and Physics* **5**, 2189–2201 (2005).

[2] Kaiser, J. *et al.* Intercomparison of Hantzsch and fiber-laser-induced-fluorescence formaldehyde measurements. *Atmospheric Measurement Techniques* **7**, 1571–1580 (2014).

[3] Dorn, H.-P., Brandenburger, U., Brauers, T. & Hausmann, M. A New In Situ Laser Long-Path Absorption Instrument for the Measurement of Tropospheric OH Radicals. *Journal of the Atmospheric Sciences* **52**, 3373–3380 (1995).

[4] Jordan, A. *et al.* A high resolution and high sensitivity proton-transfer-reaction time-of-flight mass spectrometer (PTR-TOF-MS). *International Journal of Mass Spectrometry* **286**, 122–128 (2009).

[5] Lou, S. *et al.* Atmospheric OH reactivities in the Pearl River Delta - China in summer 2006: measurement and model results. *Atmospheric Chemistry and Physics* **10**, 11243–11260 (2010).

[6] Fuchs, H. *et al.* Comparison of OH reactivity measurements in the atmospheric simulation chamber SAPHIR. *Atmospheric Measurement Techniques* **10**, 4023–4053 (2017).

[7] Dunning, T. H. Gaussian basis sets for use in correlated molecular calculations. I. The atoms boron through neon and hydrogen. *The Journal of Chemical Physics* **90**, 1007–1023 (1989).

[8] Zhao, Y. & Truhlar, D. G. The M06 suite of density functionals for main group thermochemistry, thermochemical kinetics, noncovalent interactions, excited states, and transition elements: two new functionals and systematic testing of four M06-class functionals and 12 other functionals. *Theoretical Chemistry Accounts* **120**, 215–241 (2008). https://doi.org/10.1007/s00214-007-0310-x.

[9] Alecu, I. M., Zheng, J., Zhao, Y. & Truhlar, D. G. Computational Thermochemistry: Scale Factor Databases and Scale Factors for Vibrational Frequencies Obtained from Electronic Model Chemistries. *Journal of Chemical Theory and Computation* **6**, 2872–2887 (2010).

[10] Bao, J. L. *et al.* Database of Frequency Scale Factors for Electronic Model Chemistries (Version 3 Beta 2) (2017). https://comp.chem.umn.edu/freqscale/version3b2.htm.

[11] Purvis, G. D. & Bartlett, R. J. A full coupled-cluster singles and doubles model: The inclusion of disconnected triples. *The Journal of Chemical Physics* **76**, 1910–1918 (1982).

[12] Martin, J. M. L. Ab initio total atomization energies of small molecules - towards the basis set limit. *Chemical Physics Letters* **259**, 669–678 (1996).

[13] Coote, M. L., Collins, M. A. & Radom, L. Calculation of accurate imaginary frequencies and tunnelling coefficients for hydrogen abstraction reactions using IRCmax. *Molecular Physics* **101**, 1329–1338 (2003).

[14] Frisch, M. J. *et al. Gaussian 09, Revision B.01*. Gaussian Inc., Wallingford CT (2010).

[15] Frisch, M. J. *et al. Gaussian 09, Revision A.02*. Gaussian Inc., Wallingford CT (2016).

[16] Ocaña, A. J. *et al.* Gas-phase reactivity of CH3OH toward OH at interstellar temperatures (11.7–177.5 K): experimental and theoretical study. *Physical Chemistry Chemical Physics* **21**, 6942–6957 (2019).

[17] Vereecken, L. & Peeters, J. The 1,5-H-shift in 1-butoxy: A case study in the rigorous implementation of transition state theory for a multirotamer system. *The Journal of Chemical Physics* **119**, 5159–5170 (2003).

[18] Gao, L. G., Zheng, J., Fernández-Ramos, A., Truhlar, D. G. & Xu, X. Kinetics of the Methanol Reaction with OH at Interstellar, Atmospheric, and Combustion Temperatures. *Journal of the American Chemical Society* **140**, 2906–2918 (2018).

[19] Nguyen, T. L., Ruscic, B. & Stanton, J. F. A master equation simulation for the .OH + CH3OH reaction. *The Journal of Chemical Physics* **150**, 084105 (2019).

[20] Hazra, M. K., Francisco, J. S. & Sinha, A. Gas Phase Hydrolysis of Formaldehyde To Form Methanediol: Impact of Formic Acid Catalysis. *The Journal of Physical Chemistry A* **117**, 11704–11710 (2013).

[21] Kumar, M. & Francisco, J. S. The Role of Catalysis in Alkanediol Decomposition: Implications for General Detection of Alkanediols and Their Formation in the Atmosphere. *The Journal of Physical Chemistry A* **119**, 9821–9833 (2015).

[22] Kumar, M., Anglada, J. M. & Francisco, J. S. Role of Proton Tunneling and Metal-Free Organocatalysis in the Decomposition of Methanediol: A Theoretical Study. *The Journal of Physical Chemistry A* **121**, 4318–4325 (2017).

[23] Boese, A. D. & Martin, J. M. L. Development of density functionals for thermochemical kinetics. *The Journal of Chemical Physics* **121**, 3405–3416 (2004).

[24] Veyret, B. *et al.* Kinetics and mechanism of the photo-oxidation of formaldehyde. 1. Flash photolysis study. *The Journal of Physical Chemistry* **93**, 2368–2374 (1989).

[25] Evleth, E. M., Melius, C. F., Rayez, M. T., Rayez, J. C. & Forst, W. Theoretical characterization of the reaction of hydroperoxy with formaldehyde. *The Journal of Physical Chemistry* **97**, 5040–5045 (1993).

[26] Aloisio, S. & Francisco, J. S. Complexes of Hydroxyl and Hydroperoxyl Radical with Formaldehyde, Acetaldehyde, and Acetone. *The Journal of Physical Chemistry A* **104**, 3211–3224 (2000).

[27] Gierczak, T. & Ravishankara, A. R. Does the HO2 radical react with ketones? *International Journal of Chemical Kinetics* **32**, 573–580 (2000).

[28] Olivella, S., Bofill, J. M. & Solé, A. Ab Initio Calculations on the Mechanism of the Oxidation of the Hydroxymethyl Radical by Molecular Oxygen in the Gas Phase: A Significant Reaction for Environmental Science. *Chemistry - A European Journal* **7**, 3377–3386 (2001).

[29] Tomas, A., Villenave, E. & Lesclaux, R. Reactions of the HO2 Radical with CH3CHO and CH3C(O)O2 in the Gas Phase. *The Journal of Physical Chemistry A* **105**, 3505–3514 (2001).

[30] Dibble, T. S. Mechanism and dynamics of the CH2OH + O2 reaction. *Chemical Physics Letters* **355**, 193–200 (2002).

[31] Aloisio, S. & Francisco, J. S. Complexes of Hydroperoxyl Radical with Glyoxal, Methylglyoxal, Methylvinyl Ketone, Acrolein, and Methacrolein: Possible New Sinks for HO2 in the Atmosphere? *The Journal of Physical Chemistry A* **107**, 2492–2496 (2003).

[32] Hermans, I., Nguyen, T. L., Jacobs, P. A. & Peeters, J. Tropopause Chemistry Revisited: HO2-Initiated Oxidation as an Efficient Acetone Sink. *Journal of the American Chemical Society* **126**, 9908–9909 (2004).

[33] Hermans, I., Müller, J.-F., Nguyen, T. L., Jacobs, P. A. & Peeters, J. Kinetics of -Hydroxy-alkylperoxyl Radicals in Oxidation Processes. HO2-Initiated Oxidation of Ketones/Aldehydes near the Tropopause. *The Journal of Physical Chemistry A* **109**, 4303–4311 (2005).

[34] Cours, T., Canneaux, S. & Bohr, F. Features of the potential energy surface for the reaction of HO2 radical with acetone. *International Journal of Quantum Chemistry* **107**, 1344–1354 (2007).

[35] Dillon, T. J., Pozzer, A., Vereecken, L., Crowley, J. N. & Lelieveld, J. Does acetone react with HO2 in the upper-troposphere? *Atmospheric Chemistry and Physics* **12**, 1339–1351 (2012).

[36] Sander, R. *et al.* The community atmospheric chemistry box model CAABA/MECCA-4.0. *Geoscientific Model Development* **12**, 1365–1385 (2019).

[37] Lelieveld, J., Gromov, S., Pozzer, A. & Taraborrelli, D. Global tropospheric hydroxyl distribution, budget and reactivity. *Atmospheric Chemistry and Physics* **16**, 12477–12493 (2016).

[38] Jöckel, P. *et al.* Development cycle 2 of the Modular Earth Submodel System (MESSy2). *Geoscientific Model Development* **3**, 717–752 (2010).

[39] Riva, M. *et al.* Chemical Characterization of Secondary Organic Aerosol from Oxidation of Isoprene Hydroxyhydroperoxides. *Environmental Science & Technology* **50**, 9889–9899 (2016).

[40] So, S., Wille, U. & da Silva, G. Atmospheric Chemistry of Enols: A Theoretical Study of the Vinyl Alcohol + OH + O2 Reaction Mechanism. *Environmental Science & Technology* **48**, 6694–6701 (2014).

[41] Andrews, D. U. *et al.* Photo-Tautomerization of Acetaldehyde to Vinyl Alcohol: A Potential Route to Tropospheric Acids. *Science* **337**, 1203–1206 (2012).

[42] Clubb, A. E., Jordan, M. J. T., Kable, S. H. & Osborn, D. L. Phototautomerization of Acetaldehyde to Vinyl Alcohol: A Primary Process in UV-Irradiated Acetaldehyde from 295 to 335 nm. *The Journal of Physical Chemistry Letters* **3**, 3522–3526 (2012).

[43] Calvert, J. *Mechanisms of Atmospheric Oxidation of the Oxygenates* (Oxford University Press, Inc., 2011).

[44] da Silva, G. Carboxylic Acid Catalyzed Keto-Enol Tautomerizations in the Gas Phase. *Angewandte Chemie International Edition* **49**, 7523–7525 (2010).

[45] Sindelarova, K. *et al.* Global data set of biogenic VOC emissions calculated by the MEGAN model over the last 30 years. *Atmospheric Chemistry and Physics* **14**, 9317–9341 (2014).

[46] Guenther, A. B. *et al.* The Model of Emissions of Gases and Aerosols from Nature version 2.1 (MEGAN2.1): an extended and updated framework for modeling biogenic emissions. *Geoscientific Model Development* **5**, 1471–1492 (2012).

[47] Hagemann, S. & Stacke, T. Impact of the soil hydrology scheme on simulated soil moisture memory. *Climate Dynamics* **44**, 1731–1750 (2014).

[48] Schobesberger, S. *et al.* High upward fluxes of formic acid from a boreal forest canopy. *Geophysical Research Letters* **43**, 9342–9351 (2016).

[49] Alwe, H. D. *et al.* Oxidation of Volatile Organic Compounds as the Major Source of Formic Acid in a Mixed Forest Canopy. *Geophysical Research Letters* **46**, 2940–2948 (2019).

[50] Chameides, W. L. The photochemistry of a remote marine stratiform cloud. *Journal of Geophysical Research* **89**, 4739 (1984).

[51] Nguyen, T. B. *et al.* Rapid deposition of oxidized biogenic compounds to a temperate forest. *Proceedings of the National Academy of Sciences* **112**, E392–E401 (2015).

[52] Kerkweg, A. *et al.* Technical Note: An implementation of the dry removal processes DRY DEPosition and SEDImentation in the Modular Earth Submodel System (MESSy). *Atmospheric Chemistry and Physics* **6**, 4617–4632 (2006).

[53] Katul, G. G., Palmroth, S. & Oren, R. Leaf stomatal responses to vapour pressure deficit under current and CO2-enriched atmosphere explained by the economics of gas exchange. *Plant, Cell & Environment* **32**, 968–979 (2009).

[54] Jarvis, P. G. The Interpretation of the Variations in Leaf Water Potential and Stomatal Conductance Found in Canopies in the Field. *Philosophical Transactions of the Royal Society B: Biological Sciences* **273**, 593–610 (1976).

[55] Delworth, T. L. & Manabe, S. The Influence of Potential Evaporation on the Variabilities of Simulated Soil Wetness and Climate. *Journal of Climate* **1**, 523–547 (1988).

[56] Zhang, L., Brook, J. R. & Vet, R. On ozone dry depositionwith emphasis on non-stomatal uptake and wet canopies. *Atmospheric Environment* **36**, 4787–4799 (2002).

[57] Paulot, F. *et al.* Representing sub-grid scale variations in nitrogen deposition associated with land use in a global Earth system model: implications for present and future nitrogen deposition fluxes over North America. *Atmospheric Chemistry and Physics* **18**, 17963–17978 (2018).

[58] Pozzer, A. *et al.* Technical Note: The MESSy-submodel AIRSEA calculating the air-sea exchange of chemical species. *Atmospheric Chemistry and Physics* **6**, 5435–5444 (2006).

[59] Kieber, D. J., Vaughan, G. M. & Mopper, K. Determination of formate in natural waters by a coupled enzymatic/high-performance liquid chromatographic technique. *Analytical Chemistry* **60**, 1654–1659 (1988).

[60] Funderburk, L. H., Aldwin, L. & Jencks, W. P. Mechanisms of general acid and base catalysis of the reactions of water and alcohols with formaldehyde. *Journal of the American Chemical Society* **100**, 5444–5459 (1978).

[61] Boyce, S. D. & Hoffmann, M. R. Kinetics and mechanism of the formation of hydroxymethanesulfonic acid at low pH. *The Journal of Physical Chemistry* **88**, 4740–4746 (1984).

[62] Winkelman, J. G. M., Voorwinde, O. K., Ottens, M., Beenackers, A. A. C. M. & Janssen, L. P. B. M. Kinetics and chemical equilibrium of the hydration of formaldehyde. *Chemical Engineering Science* **57**, 4067–4076 (2002).

[63] Chin, M. & Wine, P. H. A Temperature-Dependent Competitive Kinetics Study of the Aqueous-Phase Reactions of OH Radicals with Formate, Formic Acid, Acetate, Acetic Acid, and Hydrated Formaldehyde. In *Aquatic and Surface Photochemistry*, 85–96 (Lewis Publishers, 1994).

[64] Zellner, R. *et al.* Formation and reactions of oxidants in the aqueous. In Warneck, G. (ed.) *Heterogeneous and Liquid Phase Processes*, 146–152 (Springer, Berlin, 1996).

[65] Exner, M., Herrmann, H. & Zellner, R. Rate constants for the reactions of the NO3 radical with HCOOH/HCOO- and CH3COOH/CH3COO- in aqueous solution between 278 and 328 K. *Journal of Atmospheric Chemistry* **18**, 359–378 (1994).

[66] Buxton, G. V., Salmon, G. A. & Wood, N. D. A Pulse Radiolysis Study of the Chemistry of Oxysulphur Radicals in Aqueous Solution. In Restelli, G. & Angeletti, G. (eds.) *Physico-Chemical Behaviour of Atmospheric Pollutants: Air Pollution Research Reports*, 245–250 (Springer Netherlands, Dordrecht, 1990).

[67] Mouchel-Vallon, C. *et al.* CLEPS 1.0: A new protocol for cloud aqueous phase oxidation of VOC mechanisms. *Geoscientific Model Development* **10**, 1339–1362 (2017).

[68] Jacob, D. J. Chemistry of OH in remote clouds and its role in the production of formic acid and peroxymonosulfate. *Journal of Geophysical Research* **91**, 9807 (1986).

[69] Hoigné, J. & Bader, H. Rate constants of reactions of ozone with organic and inorganic compounds in waterII. *Water Research* **17**, 185–194 (1983).

[70] Burkholder, J. B. *et al.* Chemical Kinetics and Photochemical Data for Use in Atmospheric Studies, Evaluation No. 18. *JPL Publication 15-10, Jet Propulsion Lab., Pasadena, CA* (2015). http://hdl.handle.net/2014/45510.

[71] Sieg, K., Starokozhev, E., Schmidt, M. U. & PÃ¼ttmann, W. Inverse temperature dependence of Henry's law coefficients for volatile organic compounds in supercooled water. *Chemosphere* **77**, 8–14 (2009).

[72] Kelly, T. J. & Fortune, C. R. Continuous Monitoring of Gaseous Formaldehyde Using an Improved Fluorescence Approach. *International Journal of Environmental Analytical Chemistry* **54**, 249–263 (1994).

[73] Steinbacher, M. *et al.* Performance characteristics of a proton-transfer-reaction mass spectrometer (PTR-MS) derived from laboratory and field measurements. *International Journal of Mass Spectrometry* **239**, 117–128 (2004).

[74] Inomata, S. *et al.* Technical Note: Determination of formaldehyde mixing ratios in air with PTR-MS: laboratory experiments and field measurements. *Atmospheric Chemistry and Physics* **8**, 273–284 (2008).

[75] Warneke, C. *et al.* Airborne formaldehyde measurements using PTR-MS: calibration, humidity dependence, inter-comparison and initial results. *Atmospheric Measurement Techniques* **4**, 2345–2358 (2011).

[76] Lelieveld, J. *et al.* Atmospheric oxidation capacity sustained by a tropical forest. *Nature* **452**, 737–740 (2008).

[77] Whalley, L. K. *et al.* Quantifying the magnitude of a missing hydroxyl radical source in a tropical rainforest. *Atmospheric Chemistry and Physics* **11**, 7223–7233 (2011).

[78] Bauwens, M. *et al.* Nine years of global hydrocarbon emissions based on source inversion of OMI formaldehyde observations. *Atmospheric Chemistry and Physics* **16**, 10133–10158 (2016).

[79] Tost, H., Jöckel, P. & Lelieveld, J. Influence of different convection parameterisations in a GCM. *Atmospheric Chemistry and Physics* **6**, 5475–5493 (2006).

[80] Bretherton, C. S., Blossey, P. N. & Khairoutdinov, M. An Energy-Balance Analysis of Deep Convective Self-Aggregation above Uniform SST. *Journal of the Atmospheric Sciences* **62**, 4273–4292 (2005).

[81] Tobin, I., Bony, S. & Roca, R. Observational Evidence for Relationships between the Degree of Aggregation of Deep Convection, Water Vapor, Surface Fluxes, and Radiation. *Journal of Climate* **25**, 6885–6904 (2012).

[82] Mauritsen, T. & Stevens, B. Missing iris effect as a possible cause of muted hydrological change and high climate sensitivity in models. *Nature Geoscience* **8**, 346–351 (2015).

[83] Iuga, C., Alvarez-Idaboy, J. R. & Vivier-Bunge, A. Mechanism and Kinetics of the Water-Assisted Formic Acid + OH Reaction under Tropospheric Conditions. *The Journal of Physical Chemistry A* **115**, 5138–5146 (2011).

[84] Paulot, F. *et al.* Importance of secondary sources in the atmospheric budgets of formic and acetic acids. *Atmospheric Chemistry and Physics* **11**, 1989–2013 (2011).

[85] De Mazière, M. *et al.* The Network for the Detection of Atmospheric Composition Change (NDACC): history, status and perspectives. *Atmospheric Chemistry and Physics* **18**, 4935–4964 (2018).

[86] Rodgers, C. D. *Inverse Methods for Atmospheric Sounding* (WORLD SCIENTIFIC, 2000).

[87] Rothman, L. S. *et al.* The HITRAN 2008 molecular spectroscopic database. *Journal of Quantitative Spectroscopy and Radiative Transfer* **110**, 533–572 (2009).

[88] Paton-Walsh, C. *et al.* Measurements of trace gas emissions from Australian forest fires and correlations with coincident measurements of aerosol optical depth. *Journal of Geophysical Research* **110** (2005).

[89] Zander, R. *et al.* Formic acid above the Jungfraujoch during 1985–2007: observed variability, seasonality, but no long-term background evolution. *Atmospheric Chemistry and Physics* **10**, 10047–10065 (2010).

[90] Vigouroux, C. *et al.* FTIR time-series of biomass burning products (HCN, C2H6, C2H2, CH3OH, and HCOOH) at Reunion Island (21° S, 55° E) and comparisons with model data. *Atmospheric Chemistry and Physics* **12**, 10367–10385 (2012).

[91] Chang, L. *et al.* Structure of the migrating diurnal tide in the Whole Atmosphere Community Climate Model (WACCM). *Advances in Space Research* **41**, 1398–1407 (2008).

[92] Pommier, M. *et al.* HCOOH distributions from IASI for 2008–2014: comparison with ground-based FTIR measurements and a global chemistry-transport model. *Atmospheric Chemistry and Physics* **16**, 8963–8981 (2016).

[93] Rodgers, C. D. & Connor, B. J. Intercomparison of remote sounding instruments. *Journal of Geophysical Research: Atmospheres* **108** (2003).

[94] Levelt, P. F. *et al.* The ozone monitoring instrument. *IEEE Transactions on Geoscience and Remote Sensing* **44**, 1093–1101 (2006).

[95] De Smedt, I. *et al.* QA4ECV HCHO tropospheric column data from OMI (2017).

[96] Meller, R. & Moortgat, G. K. Temperature dependence of the absorption cross sections of formaldehyde between 223 and 323 K in the wavelength range 225-375 nm. *Journal of Geophysical Research: Atmospheres* **105**, 7089–7101 (2000).

[97] Spurr, R. LIDORT and VLIDORT: Linearized pseudo-spherical scalar and vector discrete ordinate radiative transfer models for use in remote sensing retrieval problems. In *Light Scattering Reviews 3*, 229–275 (Springer Berlin Heidelberg, 2008).

[98] De Smedt, I. *et al.* Diurnal, seasonal and long-term variations of global formaldehyde columns inferred from combined OMI and GOME-2 observations. *Atmospheric Chemistry and Physics* **15**, 12519–12545 (2015).

[99] Kleipool, Q. L., Dobber, M. R., de Haan, J. F. & Levelt, P. F. Earth surface reflectance climatology from 3 years of OMI data. *Journal of Geophysical Research* **113** (2008).

[100] Huijnen, V. *et al.* The global chemistry transport model TM5: description and evaluation of the tropospheric chemistry version 3.0. *Geoscientific Model Development* **3**, 445–473 (2010).

10. Raw quantum chemical data

```
***********************************************
CH2OHOH + OH : M06-2X/aug-cc-pVQZ geometry
***********************************************

OH
-------------------------------
E(CCSD(T)/Aug-CC-pVDZ) (Hartree):  -75.58401208
E(CCSD/Aug-CC-pVDZ) (Hartree):  -75.58065075
    T1 diagnostic:  0.012115
E(MP2/Aug-CC-pVDZ) (Hartree):  -75.56555498
E(MP3/Aug-CC-pVDZ) (Hartree):  -75.57785261
E(PMP2/Aug-CC-pVDZ) (Hartree):  -75.56731410
E(PMP3/Aug-CC-pVDZ) (Hartree):  -75.57891269
E(PUHF/Aug-CC-pVDZ) (Hartree):  -75.40654471
E(UHF/Aug-CC-pVDZ) (Hartree):  -75.40362085
E(CCSD(T)/Aug-CC-pVTZ) (Hartree):  -75.64558106
E(CCSD/Aug-CC-pVTZ) (Hartree):  -75.63969742
    T1 diagnostic:  0.010018
E(MP2/Aug-CC-pVTZ) (Hartree):  -75.62633534
E(MP3/Aug-CC-pVTZ) (Hartree):  -75.63790257
E(PMP2/Aug-CC-pVTZ) (Hartree):  -75.62832327
E(PMP3/Aug-CC-pVTZ) (Hartree):  -75.63904324
E(PUHF/Aug-CC-pVTZ) (Hartree):  -75.42495141
E(UHF/Aug-CC-pVTZ) (Hartree):  -75.42160059
E(CCSD(T)/Aug-CC-pVQZ) (Hartree):  -75.66449481
E(CCSD/Aug-CC-pVQZ) (Hartree):  -75.65801686
    T1 diagnostic:  0.009499
E(MP2/Aug-CC-pVQZ) (Hartree):  -75.64662073
E(MP3/Aug-CC-pVQZ) (Hartree):  -75.65673028
E(PMP2/Aug-CC-pVQZ) (Hartree):  -75.64863276
E(PMP3/Aug-CC-pVQZ) (Hartree):  -75.65786986
E(PUHF/Aug-CC-pVQZ) (Hartree):  -75.42997948
E(UHF/Aug-CC-pVQZ) (Hartree):  -75.42659099
E(UM062X/Aug-CC-pVQZ) (Hartree):  -75.73716255
Point group : C*V
Cartesian coordinates (Angs):
    O      0.000000      0.000000      0.107876
    H      0.000000      0.000000     -0.863009
Rotational constants (GHz):    0.0000000  565.5013271  565.5013271
Vibrational harmonic frequencies (cm-1):
    3774.9088 (  SG)
Zero-point correction (Hartree): 0.008600

H2O
-------------------------------
E(CCSD(T)/Aug-CC-pVDZ) (Hartree):  -76.27375874
E(CCSD/Aug-CC-pVDZ) (Hartree):  -76.26854748
    T1 diagnostic:  0.012271
E(MP2/Aug-CC-pVDZ) (Hartree):  -76.26077824
E(MP3/Aug-CC-pVDZ) (Hartree):  -76.26552888
E(RHF/Aug-CC-pVDZ) (Hartree):  -76.04138029
E(CCSD(T)/Aug-CC-pVTZ) (Hartree):  -76.34228717
E(CCSD/Aug-CC-pVTZ) (Hartree):  -76.33365549
    T1 diagnostic:  0.010000
E(MP2/Aug-CC-pVTZ) (Hartree):  -76.32895322
E(MP3/Aug-CC-pVTZ) (Hartree):  -76.33162846
E(RHF/Aug-CC-pVTZ) (Hartree):  -76.06056433
E(CCSD(T)/Aug-CC-pVQZ) (Hartree):  -76.36357038
E(CCSD/Aug-CC-pVQZ) (Hartree):  -76.35419494
    T1 diagnostic:  0.009302
E(MP2/Aug-CC-pVQZ) (Hartree):  -76.35189790
E(MP3/Aug-CC-pVQZ) (Hartree):  -76.35266331
E(RHF/Aug-CC-pVQZ) (Hartree):  -76.06594476
E(RM062X/Aug-CC-pVQZ) (Hartree):  -76.43406572
Point group : C2V
Electronic state : 1-A1
```

Cartesian coordinates (Angs):
O 0.000000 0.000000 0.116414
H -0.000000 0.761370 -0.465655
H -0.000000 -0.761370 -0.465655
Rotational constants (GHz): 833.2946100 432.5247700 284.7330100
Vibrational harmonic frequencies (cm-1):
1621.5710 3874.2767 3977.1406
Zero-point correction (Hartree): 0.021581

HCOOH

E(CCSD(T)/Aug-CC-pVDZ) (Hartree): -189.35460327
E(CCSD/Aug-CC-pVDZ) (Hartree): -189.33585238
T1 diagnostic: 0.017420
E(MP2/Aug-CC-pVDZ) (Hartree): -189.32513793
E(MP3/Aug-CC-pVDZ) (Hartree): -189.32728021
E(RHF/Aug-CC-pVDZ) (Hartree): -188.79822504
E(CCSD(T)/Aug-CC-pVTZ) (Hartree): -189.51736368
E(CCSD/Aug-CC-pVTZ) (Hartree): -189.48983123
T1 diagnostic: 0.016283
E(MP2/Aug-CC-pVTZ) (Hartree): -189.48630572
E(MP3/Aug-CC-pVTZ) (Hartree): -189.48385234
E(RHF/Aug-CC-pVTZ) (Hartree): -188.84541078
E(CCSD(T)/Aug-CC-pVQZ) (Hartree): -189.56749249
E(CCSD/Aug-CC-pVQZ) (Hartree): -189.53793915
T1 diagnostic: 0.015816
E(MP2/Aug-CC-pVQZ) (Hartree): -189.54025456
E(MP3/Aug-CC-pVQZ) (Hartree): -189.53334291
E(RHF/Aug-CC-pVQZ) (Hartree): -188.85794793
E(RM062X/Aug-CC-pVQZ) (Hartree): -189.77904290
Electronic state : 1-A
Cartesian coordinates (Angs):
C 0.135063 0.398134 0.000098
H 0.112031 1.492065 -0.000172
O 1.125570 -0.264624 -0.000029
O -1.109799 -0.088756 -0.000030
H -1.048574 -1.053829 0.000058
Rotational constants (GHz): 78.2078700 12.2278200 10.5745000
Vibrational harmonic frequencies (cm-1):
645.6403 673.2618 1075.0717
1162.0952 1317.8711 1416.2754
1868.3314 3097.6245 3796.7595
Zero-point correction (Hartree): 0.034293

O2

E(CCSD(T)/Aug-CC-pVDZ) (Hartree): -150.01953864
E(CCSD/Aug-CC-pVDZ) (Hartree): -150.00783992
T1 diagnostic: 0.017180
E(MP2/Aug-CC-pVDZ) (Hartree): -150.00162297
E(MP3/Aug-CC-pVDZ) (Hartree): -149.99987068
E(PMP2/Aug-CC-pVDZ) (Hartree): -150.00832489
E(PMP3/Aug-CC-pVDZ) (Hartree): -150.00299291
E(PUHF/Aug-CC-pVDZ) (Hartree): -149.65581650
E(UHF/Aug-CC-pVDZ) (Hartree): -149.64504328
E(CCSD(T)/Aug-CC-pVTZ) (Hartree): -150.14009459
E(CCSD/Aug-CC-pVTZ) (Hartree): -150.12173589
T1 diagnostic: 0.017054
E(MP2/Aug-CC-pVTZ) (Hartree): -150.11923543
E(MP3/Aug-CC-pVTZ) (Hartree): -150.11496264
E(PMP2/Aug-CC-pVTZ) (Hartree): -150.12629332
E(PMP3/Aug-CC-pVTZ) (Hartree): -150.11817226
E(PUHF/Aug-CC-pVTZ) (Hartree): -149.69218984
E(UHF/Aug-CC-pVTZ) (Hartree): -149.68093561
E(CCSD(T)/Aug-CC-pVQZ) (Hartree): -150.17807700
E(CCSD/Aug-CC-pVQZ) (Hartree): -150.15815697
T1 diagnostic: 0.017139
E(MP2/Aug-CC-pVQZ) (Hartree): -150.15917791
E(MP3/Aug-CC-pVQZ) (Hartree): -150.15235332
E(PMP2/Aug-CC-pVQZ) (Hartree): -150.16632728
E(PMP3/Aug-CC-pVQZ) (Hartree): -150.15557017

E(PUHF/Aug-CC-pVQZ) (Hartree): -149.70270389
E(UHF/Aug-CC-pVQZ) (Hartree): -149.69134411
E(UM062X/Aug-CC-pVQZ) (Hartree): -150.33255017
Point group : D*H
Electronic state : 3-SGG
Cartesian coordinates (Angs):
O 0.000000 0.000000 0.593860
O 0.000000 0.000000 -0.593860
Rotational constants (GHz): 0.0000000 44.7958175 44.7958175
Vibrational harmonic frequencies (cm-1):
1764.4816 (SGG)
Zero-point correction (Hartree): 0.004020

HO2

E(CCSD(T)/Aug-CC-pVDZ) (Hartree): -150.59955230
E(CCSD/Aug-CC-pVDZ) (Hartree): -150.58786865
T1 diagnostic: 0.029900
E(MP2/Aug-CC-pVDZ) (Hartree): -150.56825960
E(MP3/Aug-CC-pVDZ) (Hartree): -150.57687421
E(PMP2/Aug-CC-pVDZ) (Hartree): -150.57104087
E(PMP3/Aug-CC-pVDZ) (Hartree): -150.57851097
E(PUHF/Aug-CC-pVDZ) (Hartree): -150.20776121
E(UHF/Aug-CC-pVDZ) (Hartree): -150.20320830
E(CCSD(T)/Aug-CC-pVTZ) (Hartree): -150.72587625
E(CCSD/Aug-CC-pVTZ) (Hartree): -150.70765412
T1 diagnostic: 0.028563
E(MP2/Aug-CC-pVTZ) (Hartree): -150.69252076
E(MP3/Aug-CC-pVTZ) (Hartree): -150.69872991
E(PMP2/Aug-CC-pVTZ) (Hartree): -150.69553578
E(PMP3/Aug-CC-pVTZ) (Hartree): -150.70045929
E(PUHF/Aug-CC-pVTZ) (Hartree): -150.24531381
E(UHF/Aug-CC-pVTZ) (Hartree): -150.24037787
E(CCSD(T)/Aug-CC-pVQZ) (Hartree): -150.76491643
E(CCSD/Aug-CC-pVQZ) (Hartree): -150.74516786
T1 diagnostic: 0.028228
E(MP2/Aug-CC-pVQZ) (Hartree): -150.73404054
E(MP3/Aug-CC-pVQZ) (Hartree): -150.73738063
E(PMP2/Aug-CC-pVQZ) (Hartree): -150.73709048
E(PMP3/Aug-CC-pVQZ) (Hartree): -150.73911347
E(PUHF/Aug-CC-pVQZ) (Hartree): -150.25548760
E(UHF/Aug-CC-pVQZ) (Hartree): -150.25050549
E(UM062X/Aug-CC-pVQZ) (Hartree): -150.91559722
Point group : CS
Electronic state : 2-A"
Cartesian coordinates (Angs):
O 0.054934 0.706553 0.000000
O 0.054934 -0.598328 0.000000
H -0.878945 -0.865806 0.000000
Rotational constants (GHz): 631.3519400 34.8637000 33.0392500
Vibrational harmonic frequencies (cm-1):
1261.0103 (A') 1462.8452 (A') 3693.0970 (A')
Zero-point correction (Hartree): 0.014619

methanediol.mm

E(RM062X/Aug-CC-pVQZ) (Hartree): -190.96721248
Electronic state : 1-A
Cartesian coordinates (Angs):
C 0.000051 0.530467 0.000000
O 1.158799 -0.246531 -0.094017
O -1.158832 -0.246492 0.094033
H 0.003891 1.157073 0.891573
H -0.003820 1.156838 -0.891721
H 1.233486 -0.776278 0.703860
H -1.233596 -0.776251 -0.703835
Rotational constants (GHz): 41.9880400 10.3331200 9.1363100
Vibrational harmonic frequencies (cm-1):
355.5174 376.8627 570.7402
1018.7149 1075.6039 1118.6251
1218.3507 1374.3339 1398.7829

1458.3559 1542.2453 3073.7287
3121.6784 3869.1883 3870.0049
Zero-point correction (Hartree): 0.057963

methanediol.mt

E(RM062X/Aug-CC-pVQZ) (Hartree): -190.96292783
Electronic state : 1-A
Cartesian coordinates (Angs):
C 0.000018 0.504006 0.093864
O 1.170367 -0.225681 -0.139307
O -1.170203 -0.225779 -0.139471
H -0.000013 0.914823 1.107781
H -0.000139 1.313741 -0.628742
H 1.336943 -0.821026 0.593744
H -1.338218 -0.819885 0.594253
Rotational constants (GHz): 43.4632600 10.0405900 9.0035600
Vibrational harmonic frequencies (cm-1):
171.5193 377.0856 552.0890
1010.2489 1099.9151 1114.2485
1164.9270 1383.1862 1409.9748
1453.0667 1529.9706 3036.2048
3159.2644 3887.4967 3889.1342
Zero-point correction (Hartree): 0.057497

methanediol.pp

E(CCSD(T)/Aug-CC-pVDZ) (Hartree): -190.53807688
E(CCSD/Aug-CC-pVDZ) (Hartree): -190.52127562
T1 diagnostic: 0.013162
E(MP2/Aug-CC-pVDZ) (Hartree): -190.49913172
E(MP3/Aug-CC-pVDZ) (Hartree): -190.51377943
E(RHF/Aug-CC-pVDZ) (Hartree): -189.94565379
E(CCSD(T)/Aug-CC-pVTZ) (Hartree): -190.70756068
E(CCSD/Aug-CC-pVTZ) (Hartree): -190.68199448
T1 diagnostic: 0.011671
E(MP2/Aug-CC-pVTZ) (Hartree): -190.66790063
E(MP3/Aug-CC-pVTZ) (Hartree): -190.67747224
E(RHF/Aug-CC-pVTZ) (Hartree): -189.99338643
E(CCSD(T)/Aug-CC-pVQZ) (Hartree): -190.75857917
E(CCSD/Aug-CC-pVQZ) (Hartree): -190.73099079
T1 diagnostic: 0.011191
E(MP2/Aug-CC-pVQZ) (Hartree): -190.72329798
E(MP3/Aug-CC-pVQZ) (Hartree): -190.72780600
E(RHF/Aug-CC-pVQZ) (Hartree): -190.00581501
E(RM062X/Aug-CC-pVQZ) (Hartree): -190.96721248
Electronic state : 1-A
Cartesian coordinates (Angs):
C -0.000051 0.530467 0.000000
O -1.158799 -0.246531 -0.094017
O 1.158832 -0.246492 0.094033
H 0.003820 1.156838 -0.891721
H -0.003891 1.157073 0.891573
H -1.233486 -0.776278 0.703860
H 1.233596 -0.776251 -0.703835
Rotational constants (GHz): 41.9880400 10.3331200 9.1363100
Vibrational harmonic frequencies (cm-1):
355.5174 376.8627 570.7402
1018.7149 1075.6039 1118.6251
1218.3507 1374.3339 1398.7829
1458.3559 1542.2453 3073.7287
3121.6784 3869.1883 3870.0049
Zero-point correction (Hartree): 0.057963

methanediol.pt

E(RM062X/Aug-CC-pVQZ) (Hartree): -190.96292783
Electronic state : 1-A
Cartesian coordinates (Angs):
C -0.000018 0.504006 0.093864
O -1.170367 -0.225681 -0.139307

O 1.170203 -0.225779 -0.139471
H 0.000013 0.914823 1.107781
H 0.000139 1.313741 -0.628742
H -1.336943 -0.821026 0.593744
H 1.338218 -0.819885 0.594253
Rotational constants (GHz): 43.4632600 10.0405900 9.0035600
Vibrational harmonic frequencies (cm-1):
171.5193 377.0856 552.0890
1010.2489 1099.9151 1114.2485
1164.9270 1383.1862 1409.9748
1453.0667 1529.9706 3036.2048
3159.2644 3887.4967 3889.1342
Zero-point correction (Hartree): 0.057497

complex.CH2OHOH.OH.m045090000

E(CCSD(T)/Aug-CC-pVDZ) (Hartree): -266.13417322
E(CCSD/Aug-CC-pVDZ) (Hartree): -266.11303907
T1 diagnostic: 0.014798
E(MP2/Aug-CC-pVDZ) (Hartree): -266.07652450
E(MP3/Aug-CC-pVDZ) (Hartree): -266.10302543
E(PMP2/Aug-CC-pVDZ) (Hartree): -266.07824604
E(PMP3/Aug-CC-pVDZ) (Hartree): -266.10405290
E(PUHF/Aug-CC-pVDZ) (Hartree): -265.35861329
E(UHF/Aug-CC-pVDZ) (Hartree): -265.35571230
E(CCSD(T)/Aug-CC-pVTZ) (Hartree): -266.36501580
E(CCSD/Aug-CC-pVTZ) (Hartree): -266.33256037
T1 diagnostic: 0.012898
E(MP2/Aug-CC-pVTZ) (Hartree): -266.30587952
E(MP3/Aug-CC-pVTZ) (Hartree): -266.32661686
E(PMP2/Aug-CC-pVTZ) (Hartree): -266.30782467
E(PMP3/Aug-CC-pVTZ) (Hartree): -266.32772542
E(PUHF/Aug-CC-pVTZ) (Hartree): -265.42391111
E(UHF/Aug-CC-pVTZ) (Hartree): -265.42059156
E(CCSD(T)/Aug-CC-pVQZ) (Hartree): -266.43467714
E(CCSD/Aug-CC-pVQZ) (Hartree): -266.39958306
T1 diagnostic: 0.012294
E(MP2/Aug-CC-pVQZ) (Hartree): -266.38137057
E(MP3/Aug-CC-pVQZ) (Hartree): -266.39553070
E(PMP2/Aug-CC-pVQZ) (Hartree): -266.38333781
E(PMP3/Aug-CC-pVQZ) (Hartree): -266.39663790
E(PUHF/Aug-CC-pVQZ) (Hartree): -265.44124130
E(UHF/Aug-CC-pVQZ) (Hartree): -265.43788597
E(UM062X/Aug-CC-pVQZ) (Hartree): -266.71717942
Electronic state : 2-A
Cartesian coordinates (Angs):
C 0.984225 0.001418 0.418340
O 0.679593 -1.147384 -0.296430
O 0.430394 1.164865 -0.169675
H 0.579764 -0.022334 1.430221
H 2.069562 0.071562 0.453163
H -0.267068 -1.311982 -0.206887
H 0.802121 1.264286 -1.050504
O -2.069672 -0.096498 0.079302
H -1.412248 0.622092 -0.041605
Rotational constants (GHz): 9.3394600 4.7996800 3.4971000
Vibrational harmonic frequencies (cm-1):
89.4177 140.7120 204.0378
328.1746 432.8619 484.0936
581.0222 648.6334 1023.2507
1052.4519 1126.3971 1225.6086
1379.3239 1419.0160 1458.8735
1537.3712 3079.3999 3134.7653
3583.8392 3801.1150 3864.5973
Zero-point correction (Hartree): 0.069700

complex.CH2OHOH.OH.b

E(UM062X/Aug-CC-pVQZ) (Hartree): -266.70828175
Electronic state : 2-A
Cartesian coordinates (Angs):

C 0.837706 0.000026 0.484431
O 0.749880 1.169648 -0.273071
O 0.749991 -1.169632 -0.273055
H 1.822503 0.000087 0.941899
H 0.071659 0.000027 1.271788
H -0.162188 1.286126 -0.551336
H -0.162037 -1.286179 -0.551419
O -1.987277 -0.000013 -0.034690
H -2.696921 -0.000234 0.629019
Rotational constants (GHz): 9.0556400 4.6361700 3.4287200
Vibrational harmonic frequencies (cm-1):
97.0769 121.6565 150.4585
157.8088 215.8763 324.8368
465.9808 569.7526 1018.2417
1098.7561 1118.9527 1178.0766
1377.9407 1434.4446 1449.2279
1526.1918 2976.5106 3149.8753
3767.5232 3868.0322 3871.3198
Zero-point correction (Hartree): 0.068205

TS.CH2OHOH+OH.CHOHOH+H2O.mmc

E(CCSD(T)/Aug-CC-pVDZ) (Hartree): -266.11768459
E(CCSD/Aug-CC-pVDZ) (Hartree): -266.09469541
T1 diagnostic: 0.022049
E(MP2/Aug-CC-pVDZ) (Hartree): -266.05630639
E(MP3/Aug-CC-pVDZ) (Hartree): -266.08079210
E(PMP2/Aug-CC-pVDZ) (Hartree): -266.05973456
E(PMP3/Aug-CC-pVDZ) (Hartree): -266.08296100
E(PUHF/Aug-CC-pVDZ) (Hartree): -265.32906214
E(UHF/Aug-CC-pVDZ) (Hartree): -265.32389709
E(CCSD(T)/Aug-CC-pVTZ) (Hartree): -266.34862710
E(CCSD/Aug-CC-pVTZ) (Hartree): -266.31395330
T1 diagnostic: 0.020504
E(MP2/Aug-CC-pVTZ) (Hartree): -266.28585174
E(MP3/Aug-CC-pVTZ) (Hartree): -266.30439770
E(PMP2/Aug-CC-pVTZ) (Hartree): -266.28950597
E(PMP3/Aug-CC-pVTZ) (Hartree): -266.30666527
E(PUHF/Aug-CC-pVTZ) (Hartree): -265.39443380
E(UHF/Aug-CC-pVTZ) (Hartree): -265.38890805
E(CCSD(T)/Aug-CC-pVQZ) (Hartree): -266.41856723
E(CCSD/Aug-CC-pVQZ) (Hartree): -266.38115775
T1 diagnostic: 0.020156
E(MP2/Aug-CC-pVQZ) (Hartree): -266.36155131
E(MP3/Aug-CC-pVQZ) (Hartree): -266.37348719
E(PMP2/Aug-CC-pVQZ) (Hartree): -266.36522921
E(PMP3/Aug-CC-pVQZ) (Hartree): -266.37575557
E(PUHF/Aug-CC-pVQZ) (Hartree): -265.41174746
E(UHF/Aug-CC-pVQZ) (Hartree): -265.40618733
E(UM062X/Aug-CC-pVQZ) (Hartree): -266.70109970
Electronic state : 2-A
Cartesian coordinates (Angs):
C -0.419900 -0.020575 0.427827
O -1.177376 -0.975805 -0.228579
O -0.562377 1.255006 -0.091128
H -0.627495 0.039516 1.496787
H 0.669320 -0.380245 0.297769
H -2.110258 -0.774567 -0.106505
H -0.366304 1.216577 -1.033043
O 2.078829 -0.346309 -0.108121
H 2.241530 0.559028 0.200654
Rotational constants (GHz): 9.7863500 4.3619700 3.2830800
Vibrational harmonic frequencies (cm-1):
i732.2989 94.8260 100.7240
148.7988 260.4144 435.8889
568.8398 688.3313 932.9939
1070.1660 1143.8474 1177.0702
1298.9764 1347.7045 1427.7200
1430.7686 1684.4065 3094.0464
3780.3232 3842.3489 3846.4247
Zero-point correction (Hartree): 0.064642

TS.CH2OHOH+OH.CHOHOH+H2O.mtp

E(CCSD(T)/Aug-CC-pVDZ) (Hartree): -266.11533231
E(CCSD/Aug-CC-pVDZ) (Hartree): -266.09252655
T1 diagnostic: 0.022326
E(MP2/Aug-CC-pVDZ) (Hartree): -266.05368739
E(MP3/Aug-CC-pVDZ) (Hartree): -266.07869193
E(PMP2/Aug-CC-pVDZ) (Hartree): -266.05693536
E(PMP3/Aug-CC-pVDZ) (Hartree): -266.08076460
E(PUHF/Aug-CC-pVDZ) (Hartree): -265.32813523
E(UHF/Aug-CC-pVDZ) (Hartree): -265.32321995
E(CCSD(T)/Aug-CC-pVTZ) (Hartree): -266.34634313
E(CCSD/Aug-CC-pVTZ) (Hartree): -266.31189245
T1 diagnostic: 0.020718
E(MP2/Aug-CC-pVTZ) (Hartree): -266.28332371
E(MP3/Aug-CC-pVTZ) (Hartree): -266.30242508
E(PMP2/Aug-CC-pVTZ) (Hartree): -266.28679511
E(PMP3/Aug-CC-pVTZ) (Hartree): -266.30459456
E(PUHF/Aug-CC-pVTZ) (Hartree): -265.39358937
E(UHF/Aug-CC-pVTZ) (Hartree): -265.38831306
E(CCSD(T)/Aug-CC-pVQZ) (Hartree): -266.41633600
E(CCSD/Aug-CC-pVQZ) (Hartree): -266.37916049
T1 diagnostic: 0.020351
E(MP2/Aug-CC-pVQZ) (Hartree): -266.35908196
E(MP3/Aug-CC-pVQZ) (Hartree): -266.37158672
E(PMP2/Aug-CC-pVQZ) (Hartree): -266.36257618
E(PMP3/Aug-CC-pVQZ) (Hartree): -266.37375654
E(PUHF/Aug-CC-pVQZ) (Hartree): -265.41093648
E(UHF/Aug-CC-pVQZ) (Hartree): -265.40562672
E(UM062X/Aug-CC-pVQZ) (Hartree): -266.69912344
Electronic state : 2-A
Cartesian coordinates (Angs):

C	0.416747	-0.043122	0.427351
O	0.393155	1.231124	-0.101372
O	1.295401	-0.824931	-0.312112
H	0.667413	-0.051813	1.495309
H	-0.662786	-0.426927	0.334578
H	1.297706	1.506781	-0.278789
H	1.425029	-1.671062	0.122654
O	-2.092790	-0.355097	-0.052033
H	-1.993967	0.492979	-0.513722

Rotational constants (GHz): 10.2471800 4.3293500 3.3064800
Vibrational harmonic frequencies (cm-1):

i751.9233	82.3990	116.2714
209.3149	239.1373	249.1363
548.0837	717.0357	967.8039
1078.2452	1145.7546	1165.2020
1269.1015	1341.4747	1418.3328
1481.1348	1809.7659	3010.0160
3776.4984	3852.1978	3886.7762

Zero-point correction (Hartree): 0.064617

TS.CH2OHOH+OH.CHOHOH+H2O.ppl

E(CCSD(T)/Aug-CC-pVDZ) (Hartree): -266.11971065
E(CCSD/Aug-CC-pVDZ) (Hartree): -266.09647171
T1 diagnostic: 0.022425
E(MP2/Aug-CC-pVDZ) (Hartree): -266.05825816
E(MP3/Aug-CC-pVDZ) (Hartree): -266.08232261
E(PMP2/Aug-CC-pVDZ) (Hartree): -266.06186573
E(PMP3/Aug-CC-pVDZ) (Hartree): -266.08458766
E(PUHF/Aug-CC-pVDZ) (Hartree): -265.32945690
E(UHF/Aug-CC-pVDZ) (Hartree): -265.32402936
E(CCSD(T)/Aug-CC-pVTZ) (Hartree): -266.35076038
E(CCSD/Aug-CC-pVTZ) (Hartree): -266.31578375
T1 diagnostic: 0.020915
E(MP2/Aug-CC-pVTZ) (Hartree): -266.28786969
E(MP3/Aug-CC-pVTZ) (Hartree): -266.30598913
E(PMP2/Aug-CC-pVTZ) (Hartree): -266.29170135
E(PMP3/Aug-CC-pVTZ) (Hartree): -266.30835239

E(PUHF/Aug-CC-pVTZ) (Hartree): -265.39469537
E(UHF/Aug-CC-pVTZ) (Hartree): -265.38891382
E(CCSD(T)/Aug-CC-pVQZ) (Hartree): -266.42078558
E(CCSD/Aug-CC-pVQZ) (Hartree): -266.38305985
T1 diagnostic: 0.020574
E(MP2/Aug-CC-pVQZ) (Hartree): -266.36364560
E(MP3/Aug-CC-pVQZ) (Hartree): -266.37515676
E(PMP2/Aug-CC-pVQZ) (Hartree): -266.36750137
E(PMP3/Aug-CC-pVQZ) (Hartree): -266.37752112
E(PUHF/Aug-CC-pVQZ) (Hartree): -265.41200581
E(UHF/Aug-CC-pVQZ) (Hartree): -265.40618963
E(UM062X/Aug-CC-pVQZ) (Hartree): -266.70372012
Electronic state : 2-A
Cartesian coordinates (Angs):

C	-0.464353	-0.007938	0.486081
O	-1.079242	-1.042857	-0.180692
O	-0.501879	1.203804	-0.199370
H	-0.897225	0.052460	1.484946
H	0.669502	-0.228604	0.591293
H	-0.603812	-1.200153	-1.003288
H	-1.419295	1.454425	-0.343426
O	1.973941	-0.245312	-0.025084
H	1.894390	0.644420	-0.404839

Rotational constants (GHz): 9.4541400 4.9200400 3.5985200
Vibrational harmonic frequencies (cm-1):

i868.0321	69.7365	140.9234
272.0113	291.2540	415.1330
572.3868	733.4673	872.0630
1070.1260	1158.7311	1229.9810
1289.8284	1369.6142	1378.7452
1432.8406	1794.2785	3098.5934
3776.3678	3840.1689	3851.8829

Zero-point correction (Hartree): 0.065288

IRC information available.
IRCMax information available :
E(CCSD(T)/Aug-CC-pVDZ) (Hartree): -266.11927158
E(CCSD/Aug-CC-pVDZ) (Hartree): -266.09521375
T1 diagnostic: 0.024052
E(MP2/Aug-CC-pVDZ) (Hartree): -266.05730953
E(MP3/Aug-CC-pVDZ) (Hartree): -266.07972659
E(PMP2/Aug-CC-pVDZ) (Hartree): -266.06244655
E(PMP3/Aug-CC-pVDZ) (Hartree): -266.08285606
E(PUHF/Aug-CC-pVDZ) (Hartree): -265.32375482
E(UHF/Aug-CC-pVDZ) (Hartree): -265.31622154
E(CCSD(T)/Aug-CC-pVQZ) (Hartree): -266.42040646
E(CCSD/Aug-CC-pVQZ) (Hartree): -266.38160440
T1 diagnostic: 0.022388
E(MP2/Aug-CC-pVQZ) (Hartree): -266.36262403
E(MP3/Aug-CC-pVQZ) (Hartree): -266.37234426
E(PMP2/Aug-CC-pVQZ) (Hartree): -266.36801562
E(PMP3/Aug-CC-pVQZ) (Hartree): -266.37557970
E(PUHF/Aug-CC-pVQZ) (Hartree): -265.40609187
E(UHF/Aug-CC-pVQZ) (Hartree): -265.39818848
E(CCSD(T)/Aug-CC-pVTZ) (Hartree): -266.35032121
E(CCSD/Aug-CC-pVTZ) (Hartree): -266.31430793
T1 diagnostic: 0.022681
E(MP2/Aug-CC-pVTZ) (Hartree): -266.28682524
E(MP3/Aug-CC-pVTZ) (Hartree): -266.30316792
E(PMP2/Aug-CC-pVTZ) (Hartree): -266.29218803
E(PMP3/Aug-CC-pVTZ) (Hartree): -266.30640017
E(PUHF/Aug-CC-pVTZ) (Hartree): -265.38879541
E(UHF/Aug-CC-pVTZ) (Hartree): -265.38093097
Electronic state : 2-A
Cartesian coordinates (Angs):

C	-0.462746	-0.008221	0.483198
O	-1.078254	-1.042458	-0.176438
O	-0.504653	1.203085	-0.196163
H	-0.866590	0.045307	1.494406
H	0.729674	-0.228649	0.544965
H	-0.613362	-1.194887	-1.006766

H -1.423081 1.453171 -0.339137
O 1.964310 -0.244315 -0.025491
H 1.898612 0.643887 -0.407925
Rotational constants (GHz): 9.4827684 4.9508727 3.6142439

TS.CH2OHOH+OH.CHOHOH+H2O.ptm

E(CCSD(T)/Aug-CC-pVDZ) (Hartree): -266.11861994
E(CCSD/Aug-CC-pVDZ) (Hartree): -266.09572252
T1 diagnostic: 0.023774
E(MP2/Aug-CC-pVDZ) (Hartree): -266.05652866
E(MP3/Aug-CC-pVDZ) (Hartree): -266.08189360
E(PMP2/Aug-CC-pVDZ) (Hartree): -266.05938441
E(PMP3/Aug-CC-pVDZ) (Hartree): -266.08378843
E(PUHF/Aug-CC-pVDZ) (Hartree): -265.33203824
E(UHF/Aug-CC-pVDZ) (Hartree): -265.32773847
E(CCSD(T)/Aug-CC-pVTZ) (Hartree): -266.34976088
E(CCSD/Aug-CC-pVTZ) (Hartree): -266.31523651
T1 diagnostic: 0.022087
E(MP2/Aug-CC-pVTZ) (Hartree): -266.28628998
E(MP3/Aug-CC-pVTZ) (Hartree): -266.30577579
E(PMP2/Aug-CC-pVTZ) (Hartree): -266.28937114
E(PMP3/Aug-CC-pVTZ) (Hartree): -266.30776896
E(PUHF/Aug-CC-pVTZ) (Hartree): -265.39758268
E(UHF/Aug-CC-pVTZ) (Hartree): -265.39291284
E(CCSD(T)/Aug-CC-pVQZ) (Hartree): -266.41969725
E(CCSD/Aug-CC-pVQZ) (Hartree): -266.38244934
T1 diagnostic: 0.021727
E(MP2/Aug-CC-pVQZ) (Hartree): -266.36198453
E(MP3/Aug-CC-pVQZ) (Hartree): -266.37487763
E(PMP2/Aug-CC-pVQZ) (Hartree): -266.36508829
E(PMP3/Aug-CC-pVQZ) (Hartree): -266.37687041
E(PUHF/Aug-CC-pVQZ) (Hartree): -265.41494015
E(UHF/Aug-CC-pVQZ) (Hartree): -265.41023645
E(UM062X/Aug-CC-pVQZ) (Hartree): -266.70269575
Electronic state : 2-A
Cartesian coordinates (Angs):
C -0.503384 -0.026642 0.462162
O -1.470341 -0.772920 -0.174428
O -0.328474 1.216793 -0.125290
H -0.794696 0.054477 1.509876
H 0.503308 -0.559474 0.415529
H -1.352913 -0.668234 -1.123713
H 0.596023 1.292205 -0.382953
O 1.996650 -0.358138 -0.175604
H 2.485896 -0.644999 0.610867
Rotational constants (GHz): 10.9443700 4.1958700 3.3557400
Vibrational harmonic frequencies (cm-1):
i608.0189 50.2692 133.6232
168.9483 337.9629 372.4144
565.9008 610.3591 896.0893
1052.1954 1163.0855 1203.7661
1304.0426 1360.1867 1425.6484
1463.4284 2146.3320 3093.4452
3788.8158 3836.6566 3853.8969
Zero-point correction (Hartree): 0.065673

IRC information available.
IRCMax information available :
E(CCSD(T)/Aug-CC-pVDZ) (Hartree): -266.11848401
E(CCSD/Aug-CC-pVDZ) (Hartree): -266.09478659
T1 diagnostic: 0.026562
E(MP2/Aug-CC-pVDZ) (Hartree): -266.05495178
E(MP3/Aug-CC-pVDZ) (Hartree): -266.07938067
E(PMP2/Aug-CC-pVDZ) (Hartree): -266.05862904
E(PMP3/Aug-CC-pVDZ) (Hartree): -266.08180675
E(PUHF/Aug-CC-pVDZ) (Hartree): -265.32705797
E(UHF/Aug-CC-pVDZ) (Hartree): -265.32168706
E(CCSD(T)/Aug-CC-pVQZ) (Hartree): -266.41954609
E(CCSD/Aug-CC-pVQZ) (Hartree): -266.38129083
T1 diagnostic: 0.024632

E(MP2/Aug-CC-pVQZ) (Hartree): -266.36030944
E(MP3/Aug-CC-pVQZ) (Hartree): -266.37218751
E(PMP2/Aug-CC-pVQZ) (Hartree): -266.36423574
E(PMP3/Aug-CC-pVQZ) (Hartree): -266.37471725
E(PUHF/Aug-CC-pVQZ) (Hartree): -265.40978494
E(UHF/Aug-CC-pVQZ) (Hartree): -265.40403187
E(CCSD(T)/Aug-CC-pVTZ) (Hartree): -266.34954542
E(CCSD/Aug-CC-pVTZ) (Hartree): -266.31405175
T1 diagnostic: 0.024945
E(MP2/Aug-CC-pVTZ) (Hartree): -266.28458666
E(MP3/Aug-CC-pVTZ) (Hartree): -266.30306746
E(PMP2/Aug-CC-pVTZ) (Hartree): -266.28848875
E(PMP3/Aug-CC-pVTZ) (Hartree): -266.30559633
E(PUHF/Aug-CC-pVTZ) (Hartree): -265.39244225
E(UHF/Aug-CC-pVTZ) (Hartree): -265.38672354
Electronic state : 2-A
Cartesian coordinates (Angs):
C -0.498105 -0.027724 0.457412
O -1.465279 -0.773387 -0.169270
O -0.331507 1.216166 -0.121981
H -0.759000 0.031800 1.514953
H 0.557320 -0.542632 0.372025
H -1.364880 -0.658225 -1.120075
H 0.596054 1.299331 -0.371700
O 1.981936 -0.357457 -0.176647
H 2.477941 -0.646503 0.603498
Rotational constants (GHz): 10.9840910 4.2432726 3.3837155

TS.CH2OHOH+OH.CHOHOH+H2O.tmt

E(CCSD(T)/Aug-CC-pVDZ) (Hartree): -266.11874987
E(CCSD/Aug-CC-pVDZ) (Hartree): -266.09577815
T1 diagnostic: 0.023668
E(MP2/Aug-CC-pVDZ) (Hartree): -266.05680209
E(MP3/Aug-CC-pVDZ) (Hartree): -266.08191914
E(PMP2/Aug-CC-pVDZ) (Hartree): -266.05974867
E(PMP3/Aug-CC-pVDZ) (Hartree): -266.08385743
E(PUHF/Aug-CC-pVDZ) (Hartree): -265.33127300
E(UHF/Aug-CC-pVDZ) (Hartree): -265.32683806
E(CCSD(T)/Aug-CC-pVTZ) (Hartree): -266.34981101
E(CCSD/Aug-CC-pVTZ) (Hartree): -266.31519681
T1 diagnostic: 0.022047
E(MP2/Aug-CC-pVTZ) (Hartree): -266.28646597
E(MP3/Aug-CC-pVTZ) (Hartree): -266.30568729
E(PMP2/Aug-CC-pVTZ) (Hartree): -266.28963919
E(PMP3/Aug-CC-pVTZ) (Hartree): -266.30772483
E(PUHF/Aug-CC-pVTZ) (Hartree): -265.39670208
E(UHF/Aug-CC-pVTZ) (Hartree): -265.39189754
E(CCSD(T)/Aug-CC-pVQZ) (Hartree): -266.41979151
E(CCSD/Aug-CC-pVQZ) (Hartree): -266.38244524
T1 diagnostic: 0.021699
E(MP2/Aug-CC-pVQZ) (Hartree): -266.36219093
E(MP3/Aug-CC-pVQZ) (Hartree): -266.37481912
E(PMP2/Aug-CC-pVQZ) (Hartree): -266.36538736
E(PMP3/Aug-CC-pVQZ) (Hartree): -266.37685679
E(PUHF/Aug-CC-pVQZ) (Hartree): -265.41405370
E(UHF/Aug-CC-pVQZ) (Hartree): -265.40921470
E(UM062X/Aug-CC-pVQZ) (Hartree): -266.70282831
Electronic state : 2-A
Cartesian coordinates (Angs):
C 0.544406 0.039195 0.495890
O 0.548112 1.230489 -0.203068
O 1.114650 -1.018220 -0.190587
H 1.085968 0.150066 1.433296
H -0.545454 -0.199383 0.738204
H -0.305029 1.324710 -0.639244
H 0.797995 -0.997312 -1.098586
O -1.934806 -0.140243 -0.035534
H -2.123574 -1.089453 0.024509
Rotational constants (GHz): 9.4339100 4.8897300 3.6093300
Vibrational harmonic frequencies (cm-1):

i757.5397	49.8965	119.8157
158.8087	305.2701	391.1876
556.9512	638.9943	834.1525
1064.0216	1161.5435	1221.3069
1301.3903	1378.3051	1416.3807
1449.4450	2133.3364	3124.0616
3787.9928	3837.4429	3857.3877

Zero-point correction (Hartree): 0.065583

IRC information available.
IRCMax information available :
E(CCSD(T)/Aug-CC-pVDZ) (Hartree): -266.11858999
E(CCSD/Aug-CC-pVDZ) (Hartree): -266.09514409
T1 diagnostic: 0.025275
E(MP2/Aug-CC-pVDZ) (Hartree): -266.05581407
E(MP3/Aug-CC-pVDZ) (Hartree): -266.08035638
E(PMP2/Aug-CC-pVDZ) (Hartree): -266.05924974
E(PMP3/Aug-CC-pVDZ) (Hartree): -266.08260889
E(PUHF/Aug-CC-pVDZ) (Hartree): -265.32820576
E(UHF/Aug-CC-pVDZ) (Hartree): -265.32312818
E(CCSD(T)/Aug-CC-pVQZ) (Hartree): -266.41964081
E(CCSD/Aug-CC-pVQZ) (Hartree): -266.38169744
T1 diagnostic: 0.023364
E(MP2/Aug-CC-pVQZ) (Hartree): -266.36116707
E(MP3/Aug-CC-pVQZ) (Hartree): -266.37317260
E(PMP2/Aug-CC-pVQZ) (Hartree): -266.36485369
E(PMP3/Aug-CC-pVQZ) (Hartree): -266.37552806
E(PUHF/Aug-CC-pVQZ) (Hartree): -265.41088425
E(UHF/Aug-CC-pVQZ) (Hartree): -265.40541523
E(CCSD(T)/Aug-CC-pVTZ) (Hartree): -266.34961506
E(CCSD/Aug-CC-pVTZ) (Hartree): -266.31442670
T1 diagnostic: 0.023685
E(MP2/Aug-CC-pVTZ) (Hartree): -266.28541732
E(MP3/Aug-CC-pVTZ) (Hartree): -266.30402219
E(PMP2/Aug-CC-pVTZ) (Hartree): -266.28907972
E(PMP3/Aug-CC-pVTZ) (Hartree): -266.30637679
E(PUHF/Aug-CC-pVTZ) (Hartree): -265.39354102
E(UHF/Aug-CC-pVTZ) (Hartree): -265.38810684
Electronic state : 2-A
Cartesian coordinates (Angs):

C	0.541261	0.038246	0.493329
O	0.549019	1.230382	-0.200026
O	1.115003	-1.016833	-0.187515
H	1.061859	0.142601	1.443522
H	-0.578250	-0.197944	0.702944
H	-0.302892	1.326176	-0.639766
H	0.804907	-0.995748	-1.098216
O	-1.927697	-0.140624	-0.036053
H	-2.123794	-1.087958	0.020291

Rotational constants (GHz): 9.4591381 4.9157631 3.6225656

CHOHOH.mm

E(UM062X/Aug-CC-pVQZ) (Hartree): -190.30284411
Electronic state : 2-A
Cartesian coordinates (Angs):

C	-0.009263	0.506935	-0.151629
O	-1.168687	-0.160517	0.084666
O	1.133148	-0.232938	-0.077244
H	-0.007531	1.497483	0.284884
H	-1.081379	-1.051090	-0.269918
H	1.428798	-0.340357	0.835423

Rotational constants (GHz): 55.3908600 10.7691500 9.4371800
Vibrational harmonic frequencies (cm-1):

57.3154	391.9774	559.2706
826.1288	1084.3230	1216.9428
1220.6268	1291.4609	1421.8864
3180.9538	3751.3297	3852.3624

Zero-point correction (Hartree): 0.042954

CHOHOH.mt

```
-------------------------------
E(UM062X/Aug-CC-pVQZ) (Hartree): -190.30548854
Electronic state : 2-A
Cartesian coordinates (Angs):
    C     0.003915     0.505672     0.147268
    O     1.173715    -0.139887    -0.053708
    O    -1.076518    -0.309480    -0.079792
    H    -0.008027     1.487976    -0.315307
    H     1.076969    -1.046960     0.252996
    H    -1.870013     0.119882     0.246701
Rotational constants (GHz):   57.1698300   11.0170300    9.4336700
Vibrational harmonic frequencies (cm-1):
     276.0525          340.4898          546.6847
     945.8457         1089.0453         1160.6999
    1220.9512         1371.8299         1429.0547
    3133.9673         3854.2030         3897.1603
Zero-point correction (Hartree): 0.043891

CHOHOH.pp
-------------------------------
E(UM062X/Aug-CC-pVQZ) (Hartree): -190.30284418
Electronic state : 2-A
Cartesian coordinates (Angs):
    C     0.009265     0.506887    -0.151704
    O    -1.133101    -0.233056    -0.077222
    O     1.168725    -0.160474     0.084689
    H     0.007467     1.497490     0.284679
    H    -1.429404    -0.339392     0.835351
    H     1.081353    -1.051182    -0.269537
Rotational constants (GHz):   55.3944900   10.7688700    9.4370000
Vibrational harmonic frequencies (cm-1):
      60.8695          391.8915          559.2661
     826.1119         1084.3453         1216.8415
    1220.6000         1291.5289         1421.9214
    3180.9706         3751.4833         3852.3811
Zero-point correction (Hartree): 0.042962

CHOHOH.tp
-------------------------------
E(CCSD(T)/Aug-CC-pVDZ) (Hartree): -189.87779943
E(CCSD/Aug-CC-pVDZ) (Hartree): -189.86154507
    T1 diagnostic:  0.017288
E(MP2/Aug-CC-pVDZ) (Hartree): -189.84049088
E(MP3/Aug-CC-pVDZ) (Hartree): -189.85314286
E(PMP2/Aug-CC-pVDZ) (Hartree): -189.84187985
E(PMP3/Aug-CC-pVDZ) (Hartree): -189.85397301
E(PUHF/Aug-CC-pVDZ) (Hartree): -189.32092402
E(UHF/Aug-CC-pVDZ) (Hartree): -189.31859388
E(CCSD(T)/Aug-CC-pVTZ) (Hartree): -190.04298060
E(CCSD/Aug-CC-pVTZ) (Hartree): -190.01809951
    T1 diagnostic:  0.015983
E(MP2/Aug-CC-pVTZ) (Hartree): -190.00428703
E(MP3/Aug-CC-pVTZ) (Hartree): -190.01250374
E(PMP2/Aug-CC-pVTZ) (Hartree): -190.00575607
E(PMP3/Aug-CC-pVTZ) (Hartree): -190.01335995
E(PUHF/Aug-CC-pVTZ) (Hartree): -189.36838198
E(UHF/Aug-CC-pVTZ) (Hartree): -189.36591438
E(CCSD(T)/Aug-CC-pVQZ) (Hartree): -190.09328043
E(CCSD/Aug-CC-pVQZ) (Hartree): -190.06639701
    T1 diagnostic:  0.015533
E(MP2/Aug-CC-pVQZ) (Hartree): -190.05851869
E(MP3/Aug-CC-pVQZ) (Hartree): -190.06215484
E(PMP2/Aug-CC-pVQZ) (Hartree): -190.06000364
E(PMP3/Aug-CC-pVQZ) (Hartree): -190.06301110
E(PUHF/Aug-CC-pVQZ) (Hartree): -189.38089068
E(UHF/Aug-CC-pVQZ) (Hartree): -189.37839919
E(UM062X/Aug-CC-pVQZ) (Hartree): -190.30548856
Electronic state : 2-A
Cartesian coordinates (Angs):
    C    -0.003867     0.505686     0.147243
    O     1.076446    -0.309534    -0.079674
```

```
    O    -1.173674    -0.139863    -0.053754
    H     0.008089     1.487913    -0.315498
    H     1.870045     0.120020     0.246297
    H    -1.077106    -1.046876     0.253167
Rotational constants (GHz):   57.1702000   11.0179200   9.4343000
Vibrational harmonic frequencies (cm-1):
     275.6855          340.3432          546.7052
     945.9116          1089.1348         1160.7716
    1220.9928          1371.8201         1429.0947
    3133.9323          3854.2691         3897.2323
Zero-point correction (Hartree): 0.043891

CHOHOH.tt
-------------------------------
E(UM062X/Aug-CC-pVQZ) (Hartree): -190.30159363
Electronic state : 2-A
Cartesian coordinates (Angs):
    C    -0.000040     0.477142    -0.124970
    O    -1.103653    -0.298640     0.034607
    O     1.103701    -0.298584     0.034827
    H    -0.000072     1.435603     0.398906
    H    -1.885219     0.239710    -0.101761
    H     1.885141     0.239632    -0.102803
Rotational constants (GHz):   62.3352300   10.8685800   9.3816200
Vibrational harmonic frequencies (cm-1):
     167.5695          336.8171          544.6949
     959.4974          1097.4517         1133.4395
    1250.6366          1357.1143         1442.7841
    3041.4734          3910.1273         3910.3107
Zero-point correction (Hartree): 0.043631

TS.CH2OHOH+OH.CH2OHO+H2O.mmt
-------------------------------
E(CCSD(T)/Aug-CC-pVDZ) (Hartree): -266.11391085
E(CCSD/Aug-CC-pVDZ) (Hartree): -266.08899476
    T1 diagnostic:  0.024883
E(MP2/Aug-CC-pVDZ) (Hartree): -266.05153549
E(MP3/Aug-CC-pVDZ) (Hartree): -266.07312310
E(PMP2/Aug-CC-pVDZ) (Hartree): -266.05746070
E(PMP3/Aug-CC-pVDZ) (Hartree): -266.07644834
E(PUHF/Aug-CC-pVDZ) (Hartree): -265.31649796
E(UHF/Aug-CC-pVDZ) (Hartree): -265.30762906
E(CCSD(T)/Aug-CC-pVTZ) (Hartree): -266.34618953
E(CCSD/Aug-CC-pVTZ) (Hartree): -266.30919097
    T1 diagnostic:  0.023721
E(MP2/Aug-CC-pVTZ) (Hartree): -266.28216054
E(MP3/Aug-CC-pVTZ) (Hartree): -266.29768855
E(PMP2/Aug-CC-pVTZ) (Hartree): -266.28825652
E(PMP3/Aug-CC-pVTZ) (Hartree): -266.30108562
E(PUHF/Aug-CC-pVTZ) (Hartree): -265.38234429
E(UHF/Aug-CC-pVTZ) (Hartree): -265.37322096
E(CCSD(T)/Aug-CC-pVQZ) (Hartree): -266.41581300
E(CCSD/Aug-CC-pVQZ) (Hartree): -266.37600498
    T1 diagnostic:  0.023343
E(MP2/Aug-CC-pVQZ) (Hartree): -266.35758850
E(MP3/Aug-CC-pVQZ) (Hartree): -266.36645384
E(PMP2/Aug-CC-pVQZ) (Hartree): -266.36371329
E(PMP3/Aug-CC-pVQZ) (Hartree): -266.36984924
E(PUHF/Aug-CC-pVQZ) (Hartree): -265.39948416
E(UHF/Aug-CC-pVQZ) (Hartree): -265.39032266
E(UM062X/Aug-CC-pVQZ) (Hartree): -266.70091173
Electronic state : 2-A
Cartesian coordinates (Angs):
    C    -0.865718     0.371526     0.377010
    O    -1.092017    -0.923746    -0.107341
    O     0.103469     1.046530    -0.338523
    H    -1.761860     0.996247     0.294692
    H    -0.595134     0.255492     1.426061
    H    -1.386329    -0.864016    -1.020592
    H     1.083422     0.572848    -0.165998
    O     1.814703    -0.378152     0.104033
```

H 1.244962 -1.146778 -0.061573
Rotational constants (GHz): 11.0706000 5.2570700 3.9573500
Vibrational harmonic frequencies (cm-1):
i1664.7095 131.8599 217.1876
319.2797 414.7191 447.5109
586.8123 729.3837 886.6551
1044.2648 1079.4543 1108.2246
1258.9999 1360.0263 1433.7718
1496.8542 1574.0439 3029.7199
3112.0365 3767.0294 3862.0706
Zero-point correction (Hartree): 0.063470

IRC information available.
IRCMax information available :
E(CCSD(T)/Aug-CC-pVDZ) (Hartree): -266.11371678
E(CCSD/Aug-CC-pVDZ) (Hartree): -266.08854881
T1 diagnostic: 0.023695
E(MP2/Aug-CC-pVDZ) (Hartree): -266.05162667
E(MP3/Aug-CC-pVDZ) (Hartree): -266.07268904
E(PMP2/Aug-CC-pVDZ) (Hartree): -266.05892657
E(PMP3/Aug-CC-pVDZ) (Hartree): -266.07666319
E(PUHF/Aug-CC-pVDZ) (Hartree): -265.31530785
E(UHF/Aug-CC-pVDZ) (Hartree): -265.30448275
E(CCSD(T)/Aug-CC-pVQZ) (Hartree): -266.41562932
E(CCSD/Aug-CC-pVQZ) (Hartree): -266.37547225
T1 diagnostic: 0.022338
E(MP2/Aug-CC-pVQZ) (Hartree): -266.35760149
E(MP3/Aug-CC-pVQZ) (Hartree): -266.36587898
E(PMP2/Aug-CC-pVQZ) (Hartree): -266.36512359
E(PMP3/Aug-CC-pVQZ) (Hartree): -266.36993424
E(PUHF/Aug-CC-pVQZ) (Hartree): -265.39819231
E(UHF/Aug-CC-pVQZ) (Hartree): -265.38705796
E(CCSD(T)/Aug-CC-pVTZ) (Hartree): -266.34601989
E(CCSD/Aug-CC-pVTZ) (Hartree): -266.30868912
T1 diagnostic: 0.022671
E(MP2/Aug-CC-pVTZ) (Hartree): -266.28221139
E(MP3/Aug-CC-pVTZ) (Hartree): -266.29716309
E(PMP2/Aug-CC-pVTZ) (Hartree): -266.28969839
E(PMP3/Aug-CC-pVTZ) (Hartree): -266.30121844
E(PUHF/Aug-CC-pVTZ) (Hartree): -265.38106529
E(UHF/Aug-CC-pVTZ) (Hartree): -265.36997634
Electronic state : 2-A
Cartesian coordinates (Angs):
C -0.865685 0.371877 0.376505
O -1.092578 -0.923213 -0.106821
O 0.102956 1.046445 -0.338919
H -1.761538 0.998358 0.293857
H -0.595428 0.257332 1.425902
H -1.386562 -0.864567 -1.020279
H 1.100991 0.553545 -0.163528
O 1.813653 -0.377079 0.104029
H 1.244397 -1.145152 -0.061296
Rotational constants (GHz): 11.0835300 5.2576421 3.9590010

TS.CH2OHOH+OH.CH2OHO+H2O.mtl

E(UM062X/Aug-CC-pVQZ) (Hartree): -266.69890636
Electronic state : 2-A
Cartesian coordinates (Angs):
C 0.908784 0.373151 0.333775
O 1.245819 -0.856885 -0.215257
O -0.139665 1.004991 -0.339745
H 0.668474 0.281294 1.396018
H 1.770538 1.029746 0.206439
H 0.526075 -1.466877 -0.026560
H -1.073547 0.643531 0.052711
O -1.775756 -0.409839 0.191915
H -1.987430 -0.632733 -0.726564
Rotational constants (GHz): 11.6702000 4.9759300 3.8567600
Vibrational harmonic frequencies (cm-1):
i1430.3965 99.6042 200.9021

261.2021 394.4103 452.0133
577.6480 789.4049 1011.3768
1076.0091 1129.8657 1230.3148
1333.4910 1393.3723 1431.1310
1496.1699 1677.6729 3046.0926
3105.3107 3805.2228 3848.3659
Zero-point correction (Hartree): 0.064608

TS.CH2OHOH+OH.CH2OHO+H2O.mtt

E(UM062X/Aug-CC-pVQZ) (Hartree): -266.69847498
Electronic state : 2-A
Cartesian coordinates (Angs):
C -0.846117 0.385855 0.410057
O -1.440334 -0.769679 -0.085540
O 0.191147 0.857120 -0.382297
H -1.553061 1.221872 0.463035
H -0.499041 0.145498 1.415070
H -1.782117 -0.591563 -0.965479
H 0.959711 0.100717 -0.465835
O 1.931254 -0.511606 0.055910
H 2.494677 0.201672 0.388282
Rotational constants (GHz): 13.5998600 4.1066400 3.5022200
Vibrational harmonic frequencies (cm-1):
i1531.9022 95.1209 186.5934
232.8401 360.0958 424.0857
557.7707 791.4692 1016.0227
1064.1115 1123.4835 1224.5437
1278.9601 1371.8379 1426.7091
1487.5440 1540.2929 3021.2148
3102.4725 3815.1333 3870.6255
Zero-point correction (Hartree): 0.063768

TS.CH2OHOH+OH.CH2OHO+H2O.tmm

E(UM062X/Aug-CC-pVQZ) (Hartree): -266.69902832
Electronic state : 2-A
Cartesian coordinates (Angs):
C -0.911803 0.366619 0.350722
O -1.216488 -0.863111 -0.211664
O 0.122531 1.035242 -0.311621
H -1.787259 1.007815 0.242205
H -0.664521 0.270235 1.412034
H -0.504366 -1.476047 -0.003842
H 0.890339 0.349379 -0.525426
O 1.752114 -0.477717 0.041468
H 2.271369 0.093596 0.625230
Rotational constants (GHz): 11.4334100 5.0855000 3.8678000
Vibrational harmonic frequencies (cm-1):
i1174.9240 101.1873 199.3771
257.0928 383.6836 463.6255
547.9640 792.7513 1017.4087
1072.9590 1131.2853 1198.9460
1311.8405 1393.7621 1423.0361
1501.7890 1866.7083 3035.1392
3103.7532 3813.1502 3848.1197
Zero-point correction (Hartree): 0.064845

TS.CH2OHOH+OH.CH2OHO+H2O.tpl

E(UM062X/Aug-CC-pVQZ) (Hartree): -266.69654559
Electronic state : 2-A
Cartesian coordinates (Angs):
C 0.878676 0.392486 0.334482
O 1.047118 -0.872754 -0.269606
O -0.123161 1.066066 -0.308441
H 0.670748 0.274909 1.402955
H 1.773587 1.011421 0.199644
H 1.628260 -1.410961 0.272655
H -1.086307 0.578483 -0.123965
O -1.796234 -0.403133 0.138958

H -1.280127 -1.130197 -0.245468
Rotational constants (GHz): 10.9978100 5.3637700 3.9684200
Vibrational harmonic frequencies (cm-1):
i1630.6325 130.2850 196.7989
216.2269 334.2607 454.8506
574.7802 753.7831 908.3560
1049.0699 1115.4558 1121.6422
1263.0257 1280.5983 1450.4309
1501.9019 1567.1896 3009.1271
3049.5235 3769.5528 3887.2591
Zero-point correction (Hartree): 0.062955

CH2OHO.m

E(UM062X/Aug-CC-pVQZ) (Hartree): -190.29002724
Electronic state : 2-A
Cartesian coordinates (Angs):
C -0.100200 0.456323 0.043302
O 1.145823 -0.157084 -0.084546
O -1.169825 -0.342063 -0.040931
H -0.204299 0.971764 1.019954
H -0.152218 1.239940 -0.724777
H 1.149740 -0.956472 0.448821
Rotational constants (GHz): 50.4068200 10.8995100 9.6133000
Vibrational harmonic frequencies (cm-1):
246.8100 559.3098 770.6132
1017.2974 1131.6947 1161.4961
1309.6934 1361.3332 1417.0854
2880.1884 3003.7630 3870.4433
Zero-point correction (Hartree): 0.042669

CH2OHO.p

E(CCSD(T)/Aug-CC-pVDZ) (Hartree): -189.86572575
E(CCSD/Aug-CC-pVDZ) (Hartree): -189.85004544
T1 diagnostic: 0.022233
E(MP2/Aug-CC-pVDZ) (Hartree): -189.81856929
E(MP3/Aug-CC-pVDZ) (Hartree): -189.84013517
E(PMP2/Aug-CC-pVDZ) (Hartree): -189.82096199
E(PMP3/Aug-CC-pVDZ) (Hartree): -189.84164872
E(PUHF/Aug-CC-pVDZ) (Hartree): -189.32331283
E(UHF/Aug-CC-pVDZ) (Hartree): -189.31946959
E(CCSD(T)/Aug-CC-pVTZ) (Hartree): -190.02897549
E(CCSD/Aug-CC-pVTZ) (Hartree): -190.00523066
T1 diagnostic: 0.021193
E(MP2/Aug-CC-pVTZ) (Hartree): -189.98055487
E(MP3/Aug-CC-pVTZ) (Hartree): -189.99838076
E(PMP2/Aug-CC-pVTZ) (Hartree): -189.98314160
E(PMP3/Aug-CC-pVTZ) (Hartree): -189.99997161
E(PUHF/Aug-CC-pVTZ) (Hartree): -189.37029990
E(UHF/Aug-CC-pVTZ) (Hartree): -189.36610683
E(CCSD(T)/Aug-CC-pVQZ) (Hartree): -190.07819593
E(CCSD/Aug-CC-pVQZ) (Hartree): -190.05251579
T1 diagnostic: 0.021052
E(MP2/Aug-CC-pVQZ) (Hartree): -190.03372800
E(MP3/Aug-CC-pVQZ) (Hartree): -190.04696501
E(PMP2/Aug-CC-pVQZ) (Hartree): -190.03633704
E(PMP3/Aug-CC-pVQZ) (Hartree): -190.04855754
E(PUHF/Aug-CC-pVQZ) (Hartree): -189.38251354
E(UHF/Aug-CC-pVQZ) (Hartree): -189.37828550
E(UM062X/Aug-CC-pVQZ) (Hartree): -190.29002724
Electronic state : 2-A
Cartesian coordinates (Angs):
C 0.100200 0.456323 0.043302
O -1.145823 -0.157084 -0.084546
O 1.169825 -0.342063 -0.040931
H 0.152218 1.239940 -0.724777
H 0.204299 0.971764 1.019954
H -1.149740 -0.956472 0.448821
Rotational constants (GHz): 50.4068200 10.8995100 9.6133000
Vibrational harmonic frequencies (cm-1):

246.8099 559.3098 770.6138
1017.2974 1131.6943 1161.4959
1309.6934 1361.3333 1417.0855
2880.1884 3003.7630 3870.4433
Zero-point correction (Hartree): 0.042669

CH2OHO.t

E(UM062X/Aug-CC-pVQZ) (Hartree): -190.28590917
Electronic state : 2-A
Cartesian coordinates (Angs):
C -0.097560 0.439027 0.001443
O 1.075750 -0.328025 0.002425
O -1.195473 -0.307727 -0.002055
H -0.151064 1.096812 0.889742
H -0.145929 1.103107 -0.882098
H 1.840140 0.251936 -0.019260
Rotational constants (GHz): 53.1578300 10.8743400 9.5685800
Vibrational harmonic frequencies (cm-1):
120.1001 553.6882 743.4039
1018.6925 1154.8139 1178.3889
1232.1007 1365.7841 1419.5224
2897.8592 2916.5585 3889.0608
Zero-point correction (Hartree): 0.042123

TS.CHOHOH.CH2OHO.c

E(CCSD(T)/Aug-CC-pVDZ) (Hartree): -189.81951234
E(CCSD/Aug-CC-pVDZ) (Hartree): -189.80067273
T1 diagnostic: 0.021583
E(MP2/Aug-CC-pVDZ) (Hartree): -189.77894598
E(MP3/Aug-CC-pVDZ) (Hartree): -189.78986113
E(PMP2/Aug-CC-pVDZ) (Hartree): -189.78415852
E(PMP3/Aug-CC-pVDZ) (Hartree): -189.79288938
E(PUHF/Aug-CC-pVDZ) (Hartree): -189.25150206
E(UHF/Aug-CC-pVDZ) (Hartree): -189.24421314
E(CCSD(T)/Aug-CC-pVTZ) (Hartree): -189.98504689
E(CCSD/Aug-CC-pVTZ) (Hartree): -189.95730519
T1 diagnostic: 0.020530
E(MP2/Aug-CC-pVTZ) (Hartree): -189.94312742
E(MP3/Aug-CC-pVTZ) (Hartree): -189.94945756
E(PMP2/Aug-CC-pVTZ) (Hartree): -189.94859183
E(PMP3/Aug-CC-pVTZ) (Hartree): -189.95259451
E(PUHF/Aug-CC-pVTZ) (Hartree): -189.29919797
E(UHF/Aug-CC-pVTZ) (Hartree): -189.29156401
E(CCSD(T)/Aug-CC-pVQZ) (Hartree): -190.03474369
E(CCSD/Aug-CC-pVQZ) (Hartree): -190.00492402
T1 diagnostic: 0.020255
E(MP2/Aug-CC-pVQZ) (Hartree): -189.99679291
E(MP3/Aug-CC-pVQZ) (Hartree): -189.99843644
E(PMP2/Aug-CC-pVQZ) (Hartree): -190.00229790
E(PMP3/Aug-CC-pVQZ) (Hartree): -190.00157749
E(PUHF/Aug-CC-pVQZ) (Hartree): -189.31140572
E(UHF/Aug-CC-pVQZ) (Hartree): -189.30371975
E(UM062X/Aug-CC-pVQZ) (Hartree): -190.24752704
Electronic state : 2-A
Cartesian coordinates (Angs):
C 0.040442 0.463513 0.047766
O -1.168132 -0.131321 -0.021354
O 1.148722 -0.299785 -0.104334
H 0.024494 1.471550 -0.358726
H 0.933668 0.272262 0.950834
H -1.045536 -1.076037 0.126795
Rotational constants (GHz): 56.8131500 10.9343600 9.5948300
Vibrational harmonic frequencies (cm-1):
i1809.5382 426.5446 559.1535
755.8059 1021.5551 1106.4456
1250.3819 1295.5851 1421.0881
2324.7321 3137.3267 3833.9480
Zero-point correction (Hartree): 0.039031

TS.CHOHOH.CH2OHO.t

E(UM062X/Aug-CC-pVQZ) (Hartree): -190.24249978
Electronic state : 2-A
Cartesian coordinates (Angs):

C	0.042567	0.427317	0.037957
O	-1.098862	-0.304945	0.003654
O	1.185925	-0.266504	-0.096779
H	-0.017674	1.422872	-0.405981
H	0.926511	0.305636	0.951241
H	-1.860740	0.279178	-0.027998

Rotational constants (GHz): 61.9109700 10.6628000 9.5098100
Vibrational harmonic frequencies (cm-1):

i1835.4493	342.4600	560.6462
726.5211	1015.5678	1102.2900
1235.9497	1296.2257	1428.4024
2360.8849	3071.4056	3881.6283

Zero-point correction (Hartree): 0.038779

TS.CH2OHOH+OH.CHOHOHOH+H

E(CCSD(T)/Aug-CC-pVDZ) (Hartree): -266.04711026
E(CCSD/Aug-CC-pVDZ) (Hartree): -266.01950760
T1 diagnostic: 0.023688
E(MP2/Aug-CC-pVDZ) (Hartree): -265.98988117
E(MP3/Aug-CC-pVDZ) (Hartree): -266.00327195
E(PMP2/Aug-CC-pVDZ) (Hartree): -266.00020309
E(PMP3/Aug-CC-pVDZ) (Hartree): -266.00999323
E(PUHF/Aug-CC-pVDZ) (Hartree): -265.23283689
E(UHF/Aug-CC-pVDZ) (Hartree): -265.21942698
E(CCSD(T)/Aug-CC-pVTZ) (Hartree): -266.27897954
E(CCSD/Aug-CC-pVTZ) (Hartree): -266.23872206
T1 diagnostic: 0.022752
E(MP2/Aug-CC-pVTZ) (Hartree): -266.22007206
E(MP3/Aug-CC-pVTZ) (Hartree): -266.22668029
E(PMP2/Aug-CC-pVTZ) (Hartree): -266.23065869
E(PMP3/Aug-CC-pVTZ) (Hartree): -266.23355038
E(PUHF/Aug-CC-pVTZ) (Hartree): -265.29651972
E(UHF/Aug-CC-pVTZ) (Hartree): -265.28278275
E(CCSD(T)/Aug-CC-pVQZ) (Hartree): -266.34958860
E(CCSD/Aug-CC-pVQZ) (Hartree): -266.30639707
T1 diagnostic: 0.022571
E(MP2/Aug-CC-pVQZ) (Hartree): -266.29628649
E(MP3/Aug-CC-pVQZ) (Hartree): -266.29627937
E(PMP2/Aug-CC-pVQZ) (Hartree): -266.30693774
E(PMP3/Aug-CC-pVQZ) (Hartree): -266.30317410
E(PUHF/Aug-CC-pVQZ) (Hartree): -265.31380465
E(UHF/Aug-CC-pVQZ) (Hartree): -265.29999023
E(UM062X/Aug-CC-pVQZ) (Hartree): -266.63361271
Electronic state : 2-A
Cartesian coordinates (Angs):

C	0.215522	0.087678	0.406184
O	0.894117	-0.939183	-0.241372
O	0.177027	1.321613	-0.188426
H	-0.206572	-0.085242	1.421911
H	1.210978	0.450934	1.221107
H	1.797814	-0.659790	-0.407758
H	-0.562024	1.280762	-0.811261
O	-1.354302	-0.401997	-0.035075
H	-1.268067	-1.356200	-0.142125

Rotational constants (GHz): 8.9295400 8.7327900 5.1154900
Vibrational harmonic frequencies (cm-1):

i1557.9218	165.6724	325.1477
398.3603	426.3695	492.6434
586.5002	671.1235	937.8656
989.6752	1020.5336	1113.0994
1138.9312	1267.1585	1322.7269
1362.4710	1454.3841	2810.3547
3769.9528	3847.7420	3875.2726

Zero-point correction (Hartree): 0.063734

```
CHOHOHOO.ccm
-------------------------------
E(UM062X/Aug-CC-pVQZ) (Hartree): -340.70414905
Electronic state : 2-A
Cartesian coordinates (Angs):
   C    -0.554507    0.000001    0.429014
   O    -0.891800    1.154112   -0.212124
   O    -0.892578   -1.153592   -0.212655
   H    -0.986696   -0.000114    1.422346
   H    -0.386844    1.216248   -1.032061
   H    -0.388219   -1.215351   -1.032982
   O     0.905019   -0.000530    0.660666
   O     1.515460   -0.000088   -0.477310
Rotational constants (GHz):    7.8935500    5.1206700    4.0170200
Vibrational harmonic frequencies (cm-1):
     81.5371        260.3337        338.5196
    452.2729        503.7889        567.7031
    773.4271        815.7345       1093.1856
   1182.1884       1220.4018       1257.1352
   1380.9290       1439.3420       1446.6224
   3202.2520       3815.2983       3820.2145
Zero-point correction (Hartree): 0.053881

CHOHOHOO.hlh
-------------------------------
E(UM062X/Aug-CC-pVQZ) (Hartree): -340.70073512
Electronic state : 2-A
Cartesian coordinates (Angs):
   C     0.443083    0.043881    0.320916
   O     1.566365   -0.557511   -0.139096
   O     0.419634    1.312547   -0.151450
   H     0.306281   -0.023539    1.404685
   H     1.706205   -1.388502    0.321251
   H    -0.446315    1.685230    0.051453
   O    -0.706289   -0.714686   -0.232545
   O    -1.807794   -0.107409    0.060230
Rotational constants (GHz):   10.0198300    4.4760400    3.2904400
Vibrational harmonic frequencies (cm-1):
     93.9092        239.5912        337.4647
    407.3638        464.8906        598.7642
    645.1490        906.3863       1093.0352
   1171.7442       1279.1363       1304.6467
   1356.0744       1390.8201       1506.8601
   3054.9520       3833.7657       3890.8096
Zero-point correction (Hartree): 0.053709

CHOHOHOO.hlm
-------------------------------
E(UM062X/Aug-CC-pVQZ) (Hartree): -340.69536550
Electronic state : 2-A
Cartesian coordinates (Angs):
   C    -0.527892    0.000098    0.401105
   O    -0.915310    1.089662   -0.313802
   O    -0.916129   -1.088910   -0.314161
   H    -0.884602    0.000024    1.435745
   H    -0.670230    1.889121    0.158876
   H    -0.674474   -1.888702    0.159700
   O     0.926750   -0.000399    0.614567
   O     1.579271   -0.000481   -0.506722
Rotational constants (GHz):    8.0940700    4.9180400    3.8690700
Vibrational harmonic frequencies (cm-1):
    116.0738        197.9604        313.5372
    354.4201        466.6924        543.0429
    793.9028        871.9354       1102.3379
   1143.6773       1229.9818       1271.4241
   1355.2366       1421.9515       1481.0464
   3057.2425       3886.0023       3887.2432
Zero-point correction (Hartree): 0.053523

CHOHOHOO.llm
-------------------------------
```

E(UM062X/Aug-CC-pVQZ) (Hartree): -340.70332791
Electronic state : 2-A
Cartesian coordinates (Angs):

C	-0.535267	0.015076	0.429159
O	-0.717404	1.253167	-0.065525
O	-1.071131	-0.923517	-0.420644
H	-0.912330	-0.036326	1.448555
H	-0.328680	1.274025	-0.950135
H	-0.873210	-1.808731	-0.101272
O	0.901016	-0.284205	0.595301
O	1.553246	0.014627	-0.480644

Rotational constants (GHz): 8.0932100 5.0159200 3.9390000
Vibrational harmonic frequencies (cm-1):

131.9730	281.0084	323.2799
410.2748	485.7788	565.2131
784.0457	827.1604	1092.6636
1189.6071	1232.0977	1257.8668
1386.0428	1412.8754	1469.8193
3139.3094	3803.5323	3867.4352

Zero-point correction (Hartree): 0.053901

CHOHOHOO.lpt

E(UM062X/Aug-CC-pVQZ) (Hartree): -340.70222874
Electronic state : 2-A
Cartesian coordinates (Angs):

C	0.388615	0.027310	0.330318
O	1.406798	-0.815730	-0.035950
O	0.680664	1.296333	-0.032837
H	0.121686	0.012637	1.385270
H	1.158600	-1.728030	0.139459
H	1.059979	1.282711	-0.919390
O	-0.793432	-0.462959	-0.388146
O	-1.878025	0.015959	0.133528

Rotational constants (GHz): 9.6332800 4.3345500 3.2505300
Vibrational harmonic frequencies (cm-1):

97.1360	276.3166	346.7326
360.6855	488.9426	570.8020
638.9776	938.9475	1093.6399
1187.9024	1242.5361	1292.1778
1363.9274	1397.1449	1469.5910
3137.2664	3829.1913	3870.0898

Zero-point correction (Hartree): 0.053769

CHOHOHOO.mlt

E(CCSD(T)/Aug-CC-pVDZ) (Hartree): -339.96217171
E(CCSD/Aug-CC-pVDZ) (Hartree): -339.93089033
T1 diagnostic: 0.024772
E(MP2/Aug-CC-pVDZ) (Hartree): -339.89975105
E(MP3/Aug-CC-pVDZ) (Hartree): -339.91263126
E(PMP2/Aug-CC-pVDZ) (Hartree): -339.90274823
E(PMP3/Aug-CC-pVDZ) (Hartree): -339.91441029
E(PUHF/Aug-CC-pVDZ) (Hartree): -339.00330077
E(UHF/Aug-CC-pVDZ) (Hartree): -338.99841396
E(CCSD(T)/Aug-CC-pVTZ) (Hartree): -340.25115060
E(CCSD/Aug-CC-pVTZ) (Hartree): -340.20455706
T1 diagnostic: 0.023620
E(MP2/Aug-CC-pVTZ) (Hartree): -340.18538150
E(MP3/Aug-CC-pVTZ) (Hartree): -340.19127061
E(PMP2/Aug-CC-pVTZ) (Hartree): -340.18859881
E(PMP3/Aug-CC-pVTZ) (Hartree): -340.19313505
E(PUHF/Aug-CC-pVTZ) (Hartree): -339.08588013
E(UHF/Aug-CC-pVTZ) (Hartree): -339.08063373
E(CCSD(T)/Aug-CC-pVQZ) (Hartree): -340.33984568
E(CCSD/Aug-CC-pVQZ) (Hartree): -340.28961900
T1 diagnostic: 0.023265
E(MP2/Aug-CC-pVQZ) (Hartree): -340.28042285
E(MP3/Aug-CC-pVQZ) (Hartree): -340.27890577
E(PMP2/Aug-CC-pVQZ) (Hartree): -340.28367396
E(PMP3/Aug-CC-pVQZ) (Hartree): -340.28077134

E(PUHF/Aug-CC-pVQZ) (Hartree): -339.10797868
E(UHF/Aug-CC-pVQZ) (Hartree): -339.10268508
E(UM062X/Aug-CC-pVQZ) (Hartree): -340.70570700
Electronic state : 2-A
Cartesian coordinates (Angs):
C 0.448882 0.018563 0.363884
O 1.561231 -0.636723 -0.023875
O 0.410221 1.284076 -0.144075
H 0.347404 -0.034181 1.447394
H 1.744389 -0.410024 -0.943091
H -0.481256 1.630721 -0.010914
O -0.709938 -0.740482 -0.168747
O -1.799493 -0.069108 0.002111
Rotational constants (GHz): 10.1307900 4.4817500 3.3270800
Vibrational harmonic frequencies (cm-1):
103.4401 311.1217 368.9512
434.2546 493.9248 595.7198
644.5591 890.4662 1079.8044
1217.0695 1250.2630 1302.3845
1360.5967 1407.7005 1482.6775
3120.1913 3808.6968 3831.1147
Zero-point correction (Hartree): 0.053999

CHOHOHOO.mpp

E(CCSD(T)/Aug-CC-pVDZ) (Hartree): -339.96219244
E(CCSD/Aug-CC-pVDZ) (Hartree): -339.93090026
T1 diagnostic: 0.024780
E(MP2/Aug-CC-pVDZ) (Hartree): -339.89976674
E(MP3/Aug-CC-pVDZ) (Hartree): -339.91263457
E(PMP2/Aug-CC-pVDZ) (Hartree): -339.90276520
E(PMP3/Aug-CC-pVDZ) (Hartree): -339.91441437
E(PUHF/Aug-CC-pVDZ) (Hartree): -339.00326224
E(UHF/Aug-CC-pVDZ) (Hartree): -338.99837394
E(CCSD(T)/Aug-CC-pVTZ) (Hartree): -340.25116399
E(CCSD/Aug-CC-pVTZ) (Hartree): -340.20455821
T1 diagnostic: 0.023627
E(MP2/Aug-CC-pVTZ) (Hartree): -340.18539069
E(MP3/Aug-CC-pVTZ) (Hartree): -340.19126565
E(PMP2/Aug-CC-pVTZ) (Hartree): -340.18860930
E(PMP3/Aug-CC-pVTZ) (Hartree): -340.19313088
E(PUHF/Aug-CC-pVTZ) (Hartree): -339.08583676
E(UHF/Aug-CC-pVTZ) (Hartree): -339.08058886
E(CCSD(T)/Aug-CC-pVQZ) (Hartree): -340.33985580
E(CCSD/Aug-CC-pVQZ) (Hartree): -340.28961665
T1 diagnostic: 0.023273
E(MP2/Aug-CC-pVQZ) (Hartree): -340.28042929
E(MP3/Aug-CC-pVQZ) (Hartree): -340.27889760
E(PMP2/Aug-CC-pVQZ) (Hartree): -340.28368171
E(PMP3/Aug-CC-pVQZ) (Hartree): -340.28076396
E(PUHF/Aug-CC-pVQZ) (Hartree): -339.10793390
E(UHF/Aug-CC-pVQZ) (Hartree): -339.10263879
E(UM062X/Aug-CC-pVQZ) (Hartree): -340.70570734
Electronic state : 2-A
Cartesian coordinates (Angs):
C -0.449594 0.018894 0.364308
O -0.408746 1.284398 -0.143598
O -1.561894 -0.635542 -0.025062
H -0.350197 -0.034023 1.448082
H 0.484375 1.628065 -0.012818
H -1.741346 -0.411364 -0.945742
O 0.709521 -0.741514 -0.166607
O 1.799211 -0.069347 0.000846
Rotational constants (GHz): 10.1298300 4.4822100 3.3271400
Vibrational harmonic frequencies (cm-1):
104.6869 312.8660 370.8655
437.1547 494.6044 595.9433
645.0314 889.2438 1080.0615
1218.2116 1250.4535 1301.9222
1360.3999 1407.9329 1482.8541
3119.5005 3806.9217 3829.6572

Zero-point correction (Hartree): 0.054012

CHOHOHOO.pph

E(UM062X/Aug-CC-pVQZ) (Hartree): -340.69997383
Electronic state : 2-A
Cartesian coordinates (Angs):
C 0.376467 0.006914 0.325553
O 1.356256 -0.876133 -0.069258
O 0.619277 1.299293 -0.045964
H 0.161199 0.003223 1.391970
H 2.194474 -0.586260 0.299981
H 0.786960 1.318584 -0.995723
O -0.784126 -0.515893 -0.355709
O -1.866587 -0.004395 0.139737
Rotational constants (GHz): 9.6476000 4.3983800 3.2841200
Vibrational harmonic frequencies (cm-1):
101.2126 231.4980 345.8540
399.5951 504.1487 580.2895
647.3805 946.6576 1098.4120
1192.4014 1267.4353 1295.9603
1317.1672 1403.9463 1448.0430
3140.4875 3823.5801 3884.7253
Zero-point correction (Hartree): 0.053830

TS.CHOHOHOO.HCOOH+HO2.c

E(CCSD(T)/Aug-CC-pVDZ) (Hartree): -339.94746491
E(CCSD/Aug-CC-pVDZ) (Hartree): -339.91077072
T1 diagnostic: 0.021872
E(MP2/Aug-CC-pVDZ) (Hartree): -339.89484191
E(MP3/Aug-CC-pVDZ) (Hartree): -339.89364179
E(PMP2/Aug-CC-pVDZ) (Hartree): -339.89978007
E(PMP3/Aug-CC-pVDZ) (Hartree): -339.89631656
E(PUHF/Aug-CC-pVDZ) (Hartree): -338.96378953
E(UHF/Aug-CC-pVDZ) (Hartree): -338.95629524
E(CCSD(T)/Aug-CC-pVTZ) (Hartree): -340.23687053
E(CCSD/Aug-CC-pVTZ) (Hartree): -340.18422956
T1 diagnostic: 0.020662
E(MP2/Aug-CC-pVTZ) (Hartree): -340.18115787
E(MP3/Aug-CC-pVTZ) (Hartree): -340.17205264
E(PMP2/Aug-CC-pVTZ) (Hartree): -340.18638718
E(PMP3/Aug-CC-pVTZ) (Hartree): -340.17480947
E(PUHF/Aug-CC-pVTZ) (Hartree): -339.04674697
E(UHF/Aug-CC-pVTZ) (Hartree): -339.03882511
E(CCSD(T)/Aug-CC-pVQZ) (Hartree): -340.32520740
E(CCSD/Aug-CC-pVQZ) (Hartree): -340.26885889
T1 diagnostic: 0.020211
E(MP2/Aug-CC-pVQZ) (Hartree): -340.27602725
E(MP3/Aug-CC-pVQZ) (Hartree): -340.25936075
E(PMP2/Aug-CC-pVQZ) (Hartree): -340.28131469
E(PMP3/Aug-CC-pVQZ) (Hartree): -340.26211920
E(PUHF/Aug-CC-pVQZ) (Hartree): -339.06861260
E(UHF/Aug-CC-pVQZ) (Hartree): -339.06061664
E(UM062X/Aug-CC-pVQZ) (Hartree): -340.69060898
Electronic state : 2-A
Cartesian coordinates (Angs):
C -0.699345 0.114722 0.432139
O -0.270200 1.229341 -0.002000
O 0.894111 -0.847531 0.201267
O 1.679536 0.038015 -0.264665
H 0.860787 0.951399 -0.240980
O -1.589797 -0.582610 -0.268872
H -1.597669 -0.242865 -1.172890
H -0.776246 -0.094579 1.495194
Rotational constants (GHz): 9.8927300 4.2772800 3.3354300
Vibrational harmonic frequencies (cm-1):
i933.7756 149.2304 308.2454
475.4034 597.5000 644.7516
713.5777 792.7260 1091.9987
1158.9682 1181.2203 1340.0787

1374.1807 1389.9305 1633.4853
1944.7937 3168.5668 3812.6327
Zero-point correction (Hartree): 0.049612

TS.CHOHOHOO.HCOOH+HO2.t

E(UM062X/Aug-CC-pVQZ) (Hartree): -340.68573513
Electronic state : 2-A
Cartesian coordinates (Angs):

C	-0.703584	0.166233	0.398026
O	-0.223690	1.261037	-0.003992
O	0.867946	-0.857204	0.235086
O	1.673337	-0.014390	-0.268685
H	0.882936	0.930005	-0.272734
O	-1.556140	-0.470735	-0.412592
H	-1.923650	-1.245173	0.023290
H	-0.829407	-0.031889	1.462749

Rotational constants (GHz): 9.6828500 4.3377100 3.3468000
Vibrational harmonic frequencies (cm-1):

i941.1577	139.1340	313.7652
435.5349	601.3521	620.2187
720.4793	772.0718	1063.5465
1149.7482	1172.1937	1286.0949
1394.0160	1396.9214	1670.1445
1943.0690	3111.8053	3868.3721

Zero-point correction (Hartree): 0.049342

TS.CHOHOH+O2.HCOOH+HO2

E(CCSD(T)/Aug-CC-pVDZ) (Hartree): -339.89241587
E(CCSD/Aug-CC-pVDZ) (Hartree): -339.84838753
T1 diagnostic: 0.153230
E(MP2/Aug-CC-pVDZ) (Hartree): -339.80656936
E(MP3/Aug-CC-pVDZ) (Hartree): -339.80936636
E(PMP2/Aug-CC-pVDZ) (Hartree): -339.81175382
E(PMP3/Aug-CC-pVDZ) (Hartree): -339.81352802
E(PUHF/Aug-CC-pVDZ) (Hartree): -338.89312548
E(UHF/Aug-CC-pVDZ) (Hartree): -338.88594776
E(CCSD(T)/Aug-CC-pVTZ) (Hartree): -340.18313507
E(CCSD/Aug-CC-pVTZ) (Hartree): -340.11727859
T1 diagnostic: 0.146442
E(MP2/Aug-CC-pVTZ) (Hartree): -340.09023033
E(MP3/Aug-CC-pVTZ) (Hartree): -340.08523210
E(PMP2/Aug-CC-pVTZ) (Hartree): -340.09569997
E(PMP3/Aug-CC-pVTZ) (Hartree): -340.08941265
E(PUHF/Aug-CC-pVTZ) (Hartree): -338.97474234
E(UHF/Aug-CC-pVTZ) (Hartree): -338.96712251
E(CCSD(T)/Aug-CC-pVQZ) (Hartree): -340.27169943
E(CCSD/Aug-CC-pVQZ) (Hartree): -340.20114387
T1 diagnostic: 0.141445
E(MP2/Aug-CC-pVQZ) (Hartree): -340.18548568
E(MP3/Aug-CC-pVQZ) (Hartree): -340.17296407
E(PMP2/Aug-CC-pVQZ) (Hartree): -340.19101530
E(PMP3/Aug-CC-pVQZ) (Hartree): -340.17713439
E(PUHF/Aug-CC-pVQZ) (Hartree): -338.99710052
E(UHF/Aug-CC-pVQZ) (Hartree): -338.98940316
E(UM062X/Aug-CC-pVQZ) (Hartree): -340.63322771
Electronic state : 2-A
Cartesian coordinates (Angs):

C	1.623149	0.011300	-0.061386
O	0.940741	1.127492	0.191345
O	0.950171	-1.128810	-0.198729
H	2.641120	-0.012089	0.293253
H	0.113445	1.144738	-0.318722
H	0.094343	-1.095427	0.274639
O	-1.703738	-0.386387	0.478652
O	-1.760650	0.374577	-0.456373

Rotational constants (GHz): 8.9610900 2.9436300 2.3944000
Vibrational harmonic frequencies (cm-1):

i1007.4162	92.5421	110.5515
131.6076	220.0498	251.1557

499.6269 565.5849 611.0244
1027.8861 1202.7844 1295.2749
1351.4031 1447.3075 1560.5144
3224.5730 3383.8209 3613.2892
Zero-point correction (Hartree): 0.046905

IRC information available.
IRCMax information available :
E(CCSD(T)/Aug-CC-pVDZ) (Hartree): -339.89228249
E(CCSD/Aug-CC-pVDZ) (Hartree): -339.84990823
T1 diagnostic: 0.075571
E(MP2/Aug-CC-pVDZ) (Hartree): -339.82185092
E(MP3/Aug-CC-pVDZ) (Hartree): -339.82476581
E(PMP2/Aug-CC-pVDZ) (Hartree): -339.82677351
E(PMP3/Aug-CC-pVDZ) (Hartree): -339.82798800
E(PUHF/Aug-CC-pVDZ) (Hartree): -338.90844553
E(UHF/Aug-CC-pVDZ) (Hartree): -338.90129608
E(CCSD(T)/Aug-CC-pVQZ) (Hartree): -340.26533199
E(CCSD/Aug-CC-pVQZ) (Hartree): -340.20409429
T1 diagnostic: 0.063823
E(MP2/Aug-CC-pVQZ) (Hartree): -340.20069321
E(MP3/Aug-CC-pVQZ) (Hartree): -340.18830545
E(PMP2/Aug-CC-pVQZ) (Hartree): -340.20597982
E(PMP3/Aug-CC-pVQZ) (Hartree): -340.19157411
E(PUHF/Aug-CC-pVQZ) (Hartree): -339.01239419
E(UHF/Aug-CC-pVQZ) (Hartree): -339.00471008
E(CCSD(T)/Aug-CC-pVTZ) (Hartree): -340.17774739
E(CCSD/Aug-CC-pVTZ) (Hartree): -340.11966121
T1 diagnostic: 0.067532
E(MP2/Aug-CC-pVTZ) (Hartree): -340.10553803
E(MP3/Aug-CC-pVTZ) (Hartree): -340.10066507
E(PMP2/Aug-CC-pVTZ) (Hartree): -340.11076548
E(PMP3/Aug-CC-pVTZ) (Hartree): -340.10394344
E(PUHF/Aug-CC-pVTZ) (Hartree): -338.99010513
E(UHF/Aug-CC-pVTZ) (Hartree): -338.98249795
Electronic state : 2-A
Cartesian coordinates (Angs):
C 1.612636 0.009479 -0.050767
O 0.944156 1.122595 0.190946
O 0.953138 -1.122562 -0.200539
H 2.648500 -0.009240 0.253006
H 0.086164 1.127205 -0.289601
H 0.065046 -1.079112 0.243550
O -1.699380 -0.393413 0.482014
O -1.757355 0.381413 -0.460216
Rotational constants (GHz): 9.0025261 2.9531894 2.4048309

TS.CHOHOH.HCOOH+H.hh

E(UM062X/Aug-CC-pVQZ) (Hartree): -190.25204184
Electronic state : 2-A
Cartesian coordinates (Angs):
C -0.022355 0.422047 0.027466
O 1.089959 -0.320797 -0.039470
O -1.115368 -0.085494 -0.156306
H 0.111106 1.457409 0.348207
H 1.863132 0.205387 0.182395
H -1.636841 -0.944748 0.870808
Rotational constants (GHz): 62.0873200 10.8649400 9.6949400
Vibrational harmonic frequencies (cm-1):
i1530.8625 254.5449 471.3077
615.1636 704.6263 961.7231
1153.6834 1285.0155 1404.4464
1734.9568 3093.6728 3874.5672
Zero-point correction (Hartree): 0.035434

TS.CHOHOH.HCOOH+H.ll

E(UM062X/Aug-CC-pVQZ) (Hartree): -190.25204184
Electronic state : 2-A
Cartesian coordinates (Angs):

C 0.022355 0.422047 0.027466
O -1.089959 -0.320797 -0.039470
O 1.115368 -0.085494 -0.156306
H -0.111106 1.457410 0.348207
H -1.863132 0.205387 0.182395
H 1.636843 -0.944747 0.870808
Rotational constants (GHz): 62.0873300 10.8649400 9.6949400
Vibrational harmonic frequencies (cm-1):
i1530.8623 254.5447 471.3076
615.1636 704.6261 961.7230
1153.6834 1285.0155 1404.4464
1734.9567 3093.6728 3874.5672
Zero-point correction (Hartree): 0.035434

TS.CHOHOH.HCOOH+H.ph

E(CCSD(T)/Aug-CC-pVDZ) (Hartree): -189.83107764
E(CCSD/Aug-CC-pVDZ) (Hartree): -189.81133971
T1 diagnostic: 0.025043
E(MP2/Aug-CC-pVDZ) (Hartree): -189.79196029
E(MP3/Aug-CC-pVDZ) (Hartree): -189.79836146
E(PMP2/Aug-CC-pVDZ) (Hartree): -189.79898930
E(PMP3/Aug-CC-pVDZ) (Hartree): -189.80316863
E(PUHF/Aug-CC-pVDZ) (Hartree): -189.26790112
E(UHF/Aug-CC-pVDZ) (Hartree): -189.25936189
E(CCSD(T)/Aug-CC-pVTZ) (Hartree): -189.99553317
E(CCSD/Aug-CC-pVTZ) (Hartree): -189.96683599
T1 diagnostic: 0.024355
E(MP2/Aug-CC-pVTZ) (Hartree): -189.95485298
E(MP3/Aug-CC-pVTZ) (Hartree): -189.95667792
E(PMP2/Aug-CC-pVTZ) (Hartree): -189.96197837
E(PMP3/Aug-CC-pVTZ) (Hartree): -189.96154332
E(PUHF/Aug-CC-pVTZ) (Hartree): -189.31575222
E(UHF/Aug-CC-pVTZ) (Hartree): -189.30710940
E(CCSD(T)/Aug-CC-pVQZ) (Hartree): -190.04575948
E(CCSD/Aug-CC-pVQZ) (Hartree): -190.01498243
T1 diagnostic: 0.024207
E(MP2/Aug-CC-pVQZ) (Hartree): -190.00885141
E(MP3/Aug-CC-pVQZ) (Hartree): -190.00617012
E(PMP2/Aug-CC-pVQZ) (Hartree): -190.01603191
E(PMP3/Aug-CC-pVQZ) (Hartree): -190.01105781
E(PUHF/Aug-CC-pVQZ) (Hartree): -189.32834138
E(UHF/Aug-CC-pVQZ) (Hartree): -189.31963614
E(UM062X/Aug-CC-pVQZ) (Hartree): -190.25805258
Electronic state : 2-A
Cartesian coordinates (Angs):
C -0.019730 0.464760 0.017745
O 1.156460 -0.159403 0.020205
O -1.084591 -0.121832 -0.156615
H 0.064812 1.510737 0.301392
H 1.008177 -1.087568 -0.204092
H -1.529563 -0.961847 0.887513
Rotational constants (GHz): 56.7711600 11.2548700 9.8494000
Vibrational harmonic frequencies (cm-1):
i1551.9148 224.4546 563.2240
612.0555 724.8637 982.0912
1166.5928 1329.3047 1385.5177
1697.9135 3161.5451 3806.9072
Zero-point correction (Hartree): 0.035664

TS.CHOHOH.HCOOH+H.pl

E(UM062X/Aug-CC-pVQZ) (Hartree): -190.25805258
Electronic state : 2-A
Cartesian coordinates (Angs):
C 0.019731 0.464759 0.017743
O -1.156461 -0.159402 0.020207
O 1.084590 -0.121836 -0.156616
H -0.064809 1.510737 0.301387
H -1.008180 -1.087567 -0.204092
H 1.529575 -0.961819 0.887525

Rotational constants (GHz): 56.7713800 11.2548600 9.8494000
Vibrational harmonic frequencies (cm-1):
i1551.9383 224.4709 563.2262
612.0567 724.8686 982.0903
1166.5936 1329.3053 1385.5185
1697.9139 3161.5461 3806.9080
Zero-point correction (Hartree): 0.035664

COHOH.singlet

E(CCSD(T)/Aug-CC-pVDZ) (Hartree): -189.28945263
E(CCSD/Aug-CC-pVDZ) (Hartree): -189.27117571
T1 diagnostic: 0.018492
E(MP2/Aug-CC-pVDZ) (Hartree): -189.25351896
E(MP3/Aug-CC-pVDZ) (Hartree): -189.26220354
E(RHF/Aug-CC-pVDZ) (Hartree): -188.73546297
E(CCSD(T)/Aug-CC-pVTZ) (Hartree): -189.45094207
E(CCSD/Aug-CC-pVTZ) (Hartree): -189.42401381
T1 diagnostic: 0.016940
E(MP2/Aug-CC-pVTZ) (Hartree): -189.41386277
E(MP3/Aug-CC-pVTZ) (Hartree): -189.41808470
E(RHF/Aug-CC-pVTZ) (Hartree): -188.78183765
E(CCSD(T)/Aug-CC-pVQZ) (Hartree): -189.50061595
E(CCSD/Aug-CC-pVQZ) (Hartree): -189.47168879
T1 diagnostic: 0.016379
E(MP2/Aug-CC-pVQZ) (Hartree): -189.46750983
E(MP3/Aug-CC-pVQZ) (Hartree): -189.46722402
E(RHF/Aug-CC-pVQZ) (Hartree): -188.79421239
E(UM062X/Aug-CC-pVQZ) (Hartree): -189.71341063
Electronic state : 1-A
Cartesian coordinates (Angs):
C -0.002363 0.601946 0.000038
O 1.003191 -0.266572 0.000053
O -1.111680 -0.083212 -0.000027
H 1.825177 0.231466 -0.000391
H -0.943093 -1.044870 -0.000048
Rotational constants (GHz): 75.6727600 12.5846400 10.7902000
Vibrational harmonic frequencies (cm-1):
650.3878 665.9853 775.8713
1151.7510 1178.9295 1355.1820
1431.7146 3616.4961 3878.5717
Zero-point correction (Hartree): 0.033500

COHOH.triplet

E(CCSD(T)/Aug-CC-pVDZ) (Hartree): -189.19661759
E(CCSD/Aug-CC-pVDZ) (Hartree): -189.18112851
T1 diagnostic: 0.019829
E(MP2/Aug-CC-pVDZ) (Hartree): -189.16207786
E(MP3/Aug-CC-pVDZ) (Hartree): -189.17218006
E(PMP2/Aug-CC-pVDZ) (Hartree): -189.16352946
E(PMP3/Aug-CC-pVDZ) (Hartree): -189.17310884
E(PUHF/Aug-CC-pVDZ) (Hartree): -188.67674718
E(UHF/Aug-CC-pVDZ) (Hartree): -188.67436776
E(CCSD(T)/Aug-CC-pVTZ) (Hartree): -189.35593138
E(CCSD/Aug-CC-pVTZ) (Hartree): -189.33206721
T1 diagnostic: 0.018623
E(MP2/Aug-CC-pVTZ) (Hartree): -189.31934667
E(MP3/Aug-CC-pVTZ) (Hartree): -189.32569785
E(PMP2/Aug-CC-pVTZ) (Hartree): -189.32084816
E(PMP3/Aug-CC-pVTZ) (Hartree): -189.32663436
E(PUHF/Aug-CC-pVTZ) (Hartree): -188.72325173
E(UHF/Aug-CC-pVTZ) (Hartree): -188.72078905
E(CCSD(T)/Aug-CC-pVQZ) (Hartree): -189.40481521
E(CCSD/Aug-CC-pVQZ) (Hartree): -189.37900079
T1 diagnostic: 0.018157
E(MP2/Aug-CC-pVQZ) (Hartree): -189.37166866
E(MP3/Aug-CC-pVQZ) (Hartree): -189.37397072
E(PMP2/Aug-CC-pVQZ) (Hartree): -189.37318633
E(PMP3/Aug-CC-pVQZ) (Hartree): -189.37490610
E(PUHF/Aug-CC-pVQZ) (Hartree): -188.73565681

E(UHF/Aug-CC-pVQZ) (Hartree): -188.73317045
E(UM062X/Aug-CC-pVQZ) (Hartree): -189.62181967
Electronic state : 3-A
Cartesian coordinates (Angs):
C 0.000114 0.448736 0.174187
O 1.182485 -0.097286 -0.141156
O -1.182530 -0.097409 -0.141246
H 1.570762 -0.567966 0.606785
H -1.571077 -0.566892 0.607313
Rotational constants (GHz): 99.9934600 9.8800400 9.4790600
Vibrational harmonic frequencies (cm-1):
184.0671 408.9968 554.5190
1110.6166 1138.5761 1235.1842
1373.2973 3744.6035 3750.7037
Zero-point correction (Hartree): 0.030757

CHOH.singlet

E(CCSD(T)/Aug-CC-pVDZ) (Hartree): -114.15593420
E(CCSD/Aug-CC-pVDZ) (Hartree): -114.14458897
T1 diagnostic: 0.019616
E(MP2/Aug-CC-pVDZ) (Hartree): -114.12235727
E(MP3/Aug-CC-pVDZ) (Hartree): -114.13655629
E(RHF/Aug-CC-pVDZ) (Hartree): -113.80217319
E(CCSD(T)/Aug-CC-pVTZ) (Hartree): -114.25328786
E(CCSD/Aug-CC-pVTZ) (Hartree): -114.23672036
T1 diagnostic: 0.018208
E(MP2/Aug-CC-pVTZ) (Hartree): -114.21983790
E(MP3/Aug-CC-pVTZ) (Hartree): -114.23094025
E(RHF/Aug-CC-pVTZ) (Hartree): -113.82991072
E(CCSD(T)/Aug-CC-pVQZ) (Hartree): -114.28235596
E(CCSD/Aug-CC-pVQZ) (Hartree): -114.26459721
T1 diagnostic: 0.017679
E(MP2/Aug-CC-pVQZ) (Hartree): -114.25188105
E(MP3/Aug-CC-pVQZ) (Hartree): -114.25972061
E(RHF/Aug-CC-pVQZ) (Hartree): -113.83720231
E(UM062X/Aug-CC-pVQZ) (Hartree): -114.41600941
Electronic state : 1-A
Cartesian coordinates (Angs):
C -0.730901 -0.153842 0.000074
O 0.567599 -0.096369 -0.000063
H -1.104700 0.898345 -0.000271
H 0.949315 0.795657 0.000331
Rotational constants (GHz): 283.1500200 36.8726700 32.6242400
Vibrational harmonic frequencies (cm-1):
1019.7665 1243.7754 1376.0688
1480.0959 2812.5402 3665.5825
Zero-point correction (Hartree): 0.026422

CHOH.triplet

E(CCSD(T)/Aug-CC-pVDZ) (Hartree): -114.12343907
E(CCSD/Aug-CC-pVDZ) (Hartree): -114.11446864
T1 diagnostic: 0.020153
E(MP2/Aug-CC-pVDZ) (Hartree): -114.09424972
E(MP3/Aug-CC-pVDZ) (Hartree): -114.10763667
E(PMP2/Aug-CC-pVDZ) (Hartree): -114.09575836
E(PMP3/Aug-CC-pVDZ) (Hartree): -114.10857258
E(PUHF/Aug-CC-pVDZ) (Hartree): -113.80309330
E(UHF/Aug-CC-pVDZ) (Hartree): -113.80065827
E(CCSD(T)/Aug-CC-pVTZ) (Hartree): -114.21933192
E(CCSD/Aug-CC-pVTZ) (Hartree): -114.20541381
T1 diagnostic: 0.019201
E(MP2/Aug-CC-pVTZ) (Hartree): -114.18926685
E(MP3/Aug-CC-pVTZ) (Hartree): -114.20029284
E(PMP2/Aug-CC-pVTZ) (Hartree): -114.19084011
E(PMP3/Aug-CC-pVTZ) (Hartree): -114.20124116
E(PUHF/Aug-CC-pVTZ) (Hartree): -113.83134977
E(UHF/Aug-CC-pVTZ) (Hartree): -113.82881021
E(CCSD(T)/Aug-CC-pVQZ) (Hartree): -114.24785130
E(CCSD/Aug-CC-pVQZ) (Hartree): -114.23278042

T1 diagnostic: 0.018848
E(MP2/Aug-CC-pVQZ) (Hartree): -114.22017502
E(MP3/Aug-CC-pVQZ) (Hartree): -114.22842105
E(PMP2/Aug-CC-pVQZ) (Hartree): -114.22176158
E(PMP3/Aug-CC-pVQZ) (Hartree): -114.22936647
E(PUHF/Aug-CC-pVQZ) (Hartree): -113.83866737
E(UHF/Aug-CC-pVQZ) (Hartree): -113.83610914
E(UM062X/Aug-CC-pVQZ) (Hartree): -114.38602331
Electronic state : 3-A
Cartesian coordinates (Angs):

C	-0.716558	0.102495	0.130956
O	0.588040	-0.127137	0.013225
H	-1.467075	-0.241838	-0.572406
H	1.062103	0.643966	-0.319134

Rotational constants (GHz): 366.3597900 32.2441300 31.8322000
Vibrational harmonic frequencies (cm-1):

377.7685	1098.6325	1174.0520
1328.4396	3152.4645	3777.5920

Zero-point correction (Hartree): 0.024852

complex.HCOOH.HO2.a

E(UM062X/Aug-CC-pVQZ) (Hartree): -340.71059958
Electronic state : 2-A
Cartesian coordinates (Angs):

C	1.088931	-0.205781	-0.000153
O	0.551712	0.871105	-0.000113
O	-1.918410	-0.828016	0.000163
O	-2.121923	0.459565	0.000023
H	-1.215086	0.846401	-0.000063
O	2.401896	-0.372091	0.000060
H	2.822965	0.499364	0.000217
H	0.552329	-1.155585	-0.000302

Rotational constants (GHz): 16.0321700 2.0052500 1.7823200
Vibrational harmonic frequencies (cm-1):

64.1761	79.0221	115.8381
171.1161	235.1245	619.6777
666.3843	691.3324	1102.2413
1187.3025	1293.4842	1340.1297
1407.8792	1557.9352	1815.8635
3148.5773	3423.7153	3786.6767

Zero-point correction (Hartree): 0.051729

complex.HCOOH.HO2.b

E(CCSD(T)/Aug-CC-pVDZ) (Hartree): -339.97928981
E(CCSD/Aug-CC-pVDZ) (Hartree): -339.94636800
T1 diagnostic: 0.025372
E(MP2/Aug-CC-pVDZ) (Hartree): -339.91932770
E(MP3/Aug-CC-pVDZ) (Hartree): -339.92693313
E(PMP2/Aug-CC-pVDZ) (Hartree): -339.92259461
E(PMP3/Aug-CC-pVDZ) (Hartree): -339.92878755
E(PUHF/Aug-CC-pVDZ) (Hartree): -339.01630415
E(UHF/Aug-CC-pVDZ) (Hartree): -339.01101948
E(CCSD(T)/Aug-CC-pVTZ) (Hartree): -340.26854710
E(CCSD/Aug-CC-pVTZ) (Hartree): -340.22008758
T1 diagnostic: 0.024424
E(MP2/Aug-CC-pVTZ) (Hartree): -340.20485554
E(MP3/Aug-CC-pVTZ) (Hartree): -340.20540734
E(PMP2/Aug-CC-pVTZ) (Hartree): -340.20836544
E(PMP3/Aug-CC-pVTZ) (Hartree): -340.20735659
E(PUHF/Aug-CC-pVTZ) (Hartree): -339.10012441
E(UHF/Aug-CC-pVTZ) (Hartree): -339.09446038
E(CCSD(T)/Aug-CC-pVQZ) (Hartree): -340.35730952
E(CCSD/Aug-CC-pVQZ) (Hartree): -340.30523094
T1 diagnostic: 0.024078
E(MP2/Aug-CC-pVQZ) (Hartree): -340.30003474
E(MP3/Aug-CC-pVQZ) (Hartree): -340.29313317
E(PMP2/Aug-CC-pVQZ) (Hartree): -340.30358382
E(PMP3/Aug-CC-pVQZ) (Hartree): -340.29508510
E(PUHF/Aug-CC-pVQZ) (Hartree): -339.12247625

E(UHF/Aug-CC-pVQZ) (Hartree): -339.11676187
E(UM062X/Aug-CC-pVQZ) (Hartree): -340.72033767
Point group : CS
Electronic state : 2-A"
Cartesian coordinates (Angs):

C	-1.526800	-0.010221	0.000000
H	-2.618015	0.019379	0.000000
O	-0.911602	-1.055671	0.000000
O	-0.993743	1.179356	0.000000
H	-0.000000	1.093560	-0.000000
H	0.702310	-1.006917	-0.000000
O	1.667141	-0.713157	-0.000000
O	1.622768	0.583886	-0.000000

Rotational constants (GHz): 9.1462600 3.3329700 2.4428000
Vibrational harmonic frequencies (cm-1):

105.8469 (A")	181.7186 (A')	227.1010 (A")
243.7568 (A')	339.1231 (A')	722.4485 (A')
844.2478 (A")	973.4398 (A")	1101.9430 (A")
1290.5418 (A')	1330.0814 (A')	1417.8565 (A')
1475.3874 (A')	1631.5078 (A')	1781.3481 (A')
2957.9731 (A')	3125.9116 (A')	3204.4428 (A')

Zero-point correction (Hartree): 0.052295
